# Genome of *Tripterygium wilfordii* and identification of cytochrome P450 involved in triptolide biosynthesis

**DOI:** 10.1038/s41467-020-14776-1

**Published:** 2020-02-20

**Authors:** Lichan Tu, Ping Su, Zhongren Zhang, Linhui Gao, Jiadian Wang, Tianyuan Hu, Jiawei Zhou, Yifeng Zhang, Yujun Zhao, Yuan Liu, Yadi Song, Yuru Tong, Yun Lu, Jian Yang, Cao Xu, Meirong Jia, Reuben J. Peters, Luqi Huang, Wei Gao

**Affiliations:** 1 School of Traditional Chinese Medicine, Capital Medical University, Beijing, China; 2 School of Pharmaceutical Sciences, Capital Medical University, Beijing, China; 3 State Key Laboratory Breeding Base of Dao-di Herbs, National Resource Center for Chinese Materia Medica, China Academy of Chinese Medical Sciences, Beijing, China; 4 Novogene Bioinformatics Institute, Beijing, China; 5 University of Chinese Academy of Sciences, Beijing, China; 6 Roy J. Carver Department of Biochemistry, Biophysics & Molecular Biology, Iowa State University, Ames, IA USA; 7 Advanced Innovation Center for Human Brain Protection, Capital Medical University, Beijing, China; 8 Present Address: Department of Chemistry, The Scripps Research Institute, Jupiter, Florida USA

**Keywords:** Metabolic engineering, Genome, Secondary metabolism

## Abstract

Triptolide is a trace natural product of *Tripterygium wilfordii*. It has antitumor activities, particularly against pancreatic cancer cells. Identification of genes and elucidation of the biosynthetic pathway leading to triptolide are the prerequisite for heterologous bioproduction. Here, we report a reference-grade genome of *T. wilfordii* with a contig N50 of 4.36 Mb. We show that copy numbers of triptolide biosynthetic pathway genes are impacted by a recent whole-genome triplication event. We further integrate genomic, transcriptomic, and metabolomic data to map a gene-to-metabolite network. This leads to the identification of a cytochrome P450 (CYP728B70) that can catalyze oxidation of a methyl to the acid moiety of dehydroabietic acid in triptolide biosynthesis. We think the genomic resource and the candidate genes reported here set the foundation to fully reveal triptolide biosynthetic pathway and consequently the heterologous bioproduction.

## Abstract (web summary)

*Tripterygium wilfordii* is a medical plant that can produce antitumor activity compound triptolide. Here, the authors assemble its genome and identify a cytochrome P450 that can catalyze oxidation of a methyl to the acid moiety of dehydroabietic acid in triptolide biosynthesis by integrating multi-omics data.

## Introduction

*Tripterygium wilfordii*, a perennial twining shrub of the Celastrales, has been used medicinally for centuries, mainly to treat rheumatoid arthritis^1^. It has been known to be a rich source of specialized metabolites. Two of these (triptolide and celastrol) are among five natural products highlighted for their great potential to be developed into pharmaceuticals^2^. Indeed, triptolide has been demonstrated to possess important therapeutic potential with anti-inflammatory, immunosuppressive, and antitumor activities, as well as to exhibit potentially medically relevant activity for central nervous system diseases (e.g. Parkinson’s and Alzheimer’s diseases)^2–8^. Moreover, several derivatives of triptolide have undergone clinical trials^9–11^.

Currently, triptolide only can be extracted from *T. wilfordii* with extremely low yields, ranging from 0.0001% to 0.002% of dry weight biomass^12,13^, and the plant cannot be cultivated at a large scale as contamination with its pollen renders honey poisonous, which has frequently causes poisoning events in areas where this medicinal plant is cultivated. Although significant efforts have been devoted to improving chemical synthesis, current routes are limited to yields of less than 1.64% due to the structural and stereochemical complexity of triptolide^14–17^. Accordingly, further investigation of its pharmaceutical utility is severely limited by a shortage of supply. While suspension cultures, tissue cultures, and adventitious root cultures have been investigated as alternative sources of this bioactive diterpenoid^18–22^, a more promising approach to obtaining such structurally complex natural products is metabolic engineering. This can be attempted in the native plant or accomplished via a synthetic biology strategy, involving reconstitution in a suitably engineered microbial chassis organism, which can establish a sustainable and reliable means of production^23–26^. However, this latter approach requires elucidation of the relevant biosynthetic pathway.

Triptolide is an abietane-type diterpenoid, produced via initial cyclization of (*E*,*E*,*E*)-geranylgeranyl diphosphate (GGPP) to copalyl diphosphate (CPP), with subsequent cyclization to the abietane-type diene olefin miltiradiene^27,28^. The 1,4-diene arrangement of the miltiradiene C ring leaves this poised for aromatization^29^, which most likely occurs spontaneously^30^. Along with conversion of carbon-18 (C-18) from a methyl to carboxylic acid, this forms dehydroabietic acid, followed by oxidative 1,2-migration of C-18 (from C-4 → C-3) and further transformation to the phenolic triepoxide triptolide^27,28^. At present, elucidation of the triptolide pathway relies on transcriptomes, which has only led to identification of the relevant diterpene synthases, namely the relevant CPP synthase (CPS1) and miltiradiene synthase (MS)^28,31^, with subsequently acting enzymes, such as cytochrome P450s (CYPs), involved in further biosynthesis of the highly functionalized triptolide remaining enigmatic. CYPs form the largest family of enzymes in plants, playing manifold roles in their complex metabolism^32^. Indeed, there are still CYP families, defined as phylogenetic clades with <40% amino acid sequence identity between them^32^, whose function remains unknown, with no biochemical activity yet assigned to any member^32^. Recently, whole-genome sequencing provides a comprehensive genetic resource and has become a practical approach to not only elucidation of natural product biosynthetic pathways, but also insight into their evolution, as well as improvement of their production^33,34^. Nevertheless, this must be coupled to other information directed more specifically at the natural product of interest.

Here we first present a high-quality reference-grade genome of *T. wilfordii* and show that duplications of triptolide biosynthetic pathway genes are almost all generated by a recent whole-genome triplication event. We then map a gene-to-metabolite network by integrating genomic, transcriptomic, and metabolomic data. Next, we combine the synthetic biology tools, RNAi knock-down, and overexpression to identify a cytochrome P450 (CYP728B70) that can catalyze oxidation of C-18 from a methyl to the acid moiety of dehydroabietic acid in triptolide biosynthesis. This work provides the genomic resource and the candidate genes that may contribute to fully elucidation of the triptolide biosynthetic pathway and consequently lead to heterologous bioproduction.

## Results

### Genome assembly and annotation

Based on the k-mer distribution analysis, we estimated the genome size of *T. wilfordii* to be ~365.95 Mb with a high level of heterozygosity (1.95%) and repetition (48.87%), indicating the genome assembly was complicated (Supplementary Fig. 1 and Supplementary Table 1). The genome of *T. wilfordii* was sequenced using PacBio (read length of 60 kb, ~207.10× coverage) and 10X Genomics (~327.23× coverage) (Supplementary Table 2). The total length of the final assembly was 348.38 Mb with 467 contigs and a contig N50 of 4.36 Mb (Supplementary Table 3). Assessment of the completeness of the genome assembly with CEGMA^35^ indicated 96.77% coverage of the conserved core eukaryotic genes (Supplementary Table 5), and BUSCO^36^ results indicated that the genome was 95.10% complete (Supplementary Table 6). Additionally, 97.06% of the transcriptome can be mapped back to the assembly, further supporting a high level of genome coverage (Supplementary Table 7 and Supplementary Note 1).

**Fig. 1:**
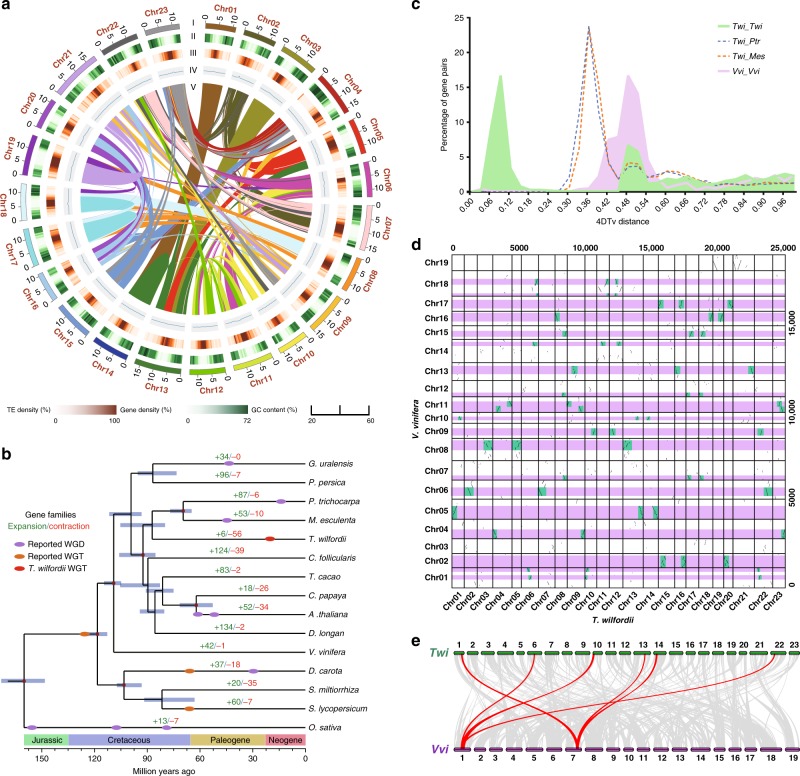
Genome evolution of *T. wilfordii*. **a** Distribution of *T. wilfordii* genomic features. (I) Circular representation of the pseudomolecule. (II–IV) gene density (500 kb window), percentage of repeats (500 kb window), and GC content (500 kb window). (V) Each linking line in the center of the circle connects a pair of homologous genes. **b** Inferred phylogenetic tree with 514 single-copy genes of 15 plant species. Gene family expansions are indicated in green, and gene family contractions are indicated in red. The timing of whole-genome duplication (WGD) and the timing of whole-genome triplication (WGT) are superimposed on the tree. Divergence times are estimated by Maximum Likelihood (PAML). **c** Distribution of 4DTv shown in colored lines as indicated. **d** Syntenic dot plots show a 3–1 chromosomal relationship between *T. wilfordii* genome and *V. vinifera* genome. **e** Macrosynteny between *T. wilfordii* and *V. vinifera* karyotypes. Source data are provided as a Source Data file.

The *T. wilfordii* assembly was further refined using high-throughput chromosome conformation capture (Hi-C) data, comprised of 321 scaffolds with a scaffold N50 of 13.52 Mb (Table 1). As a result, 315.08 Mb of the assembly and 99.92% of the genes were distributed across 23 chromosome-level pseudomolecules (Fig. 1a, Table 1 and Supplementary Data 1).

**Table 1:** Summary of *T. wilfordii* genome assembly and annotation.

	Number	Size
Genome assembly		
Total contigs	467	348.38 Mb
Contig N50	15	4.36 Mb
Contig N90	137	265 kb
Total scaffolds	321	348.53 Mb
Scaffold N50	12	13.52 Mb
Scaffold N90	23	10.83 Mb
Pseudochromosomes	23	315.08 Mb
Genome annotation		
Repetitive sequences	52.36%	182.52 Mb
Noncoding RNAs	4235	1.12 Mb
Protein-coding genes	28,321	109.30 Mb
Genes in pseudochromosomes	28,297 (99.92%)	109.24 Mb

We were able to annotate 28,321 protein-coding genes, with an average sequence length of 3338 bp, similar to those of other reported plants (Supplementary Tables 8 and 9). On average, each predicted gene contains 5.44 exons with an average sequence length of 228 bp. A total of 182.52 Mb of repetitive elements occupying 52.36% of the *T. wilfordii* genome were annotated (Supplementary Fig. 2 and Supplementary Note 2). The majority of the repeats are long terminal repeats (LTRs) (34.26% of the genome; Supplementary Table 10). Approximately 99.6% of the genes were functionally annotated by similarity searches against homologous sequences and protein domains (Supplementary Table 11). In addition, we identified noncoding RNA (ncRNA) genes, including 2,563 rRNA, 407 tRNA, 373 miRNA, and 892 snRNA genes (Supplementary Table 12). These results further support the completeness of our *T. wilfordii* genome sequence and a schematic representation of the genome is given in Fig. 1a.

### Genome evolution contributed to formation of triptolide

To investigate the evolution of *T. wilfordii*, we constructed a phylogenetic tree and estimated the divergence times of 15 plant species using 514 single-copy genes (Fig. 1b, Supplementary Fig. 4 and Supplementary Note 3). Phylogenomic analysis showed that *T. wilfordii* was most related to the ancestor of *Manihot esculenta* and *Populus trichocarpa*, with an estimated divergence time of 87.1 million years ago (MYA). Gene family expansion and contraction was examined using CAFÉ (Fig. 1b and Supplementary Note 3). Among the *T. wilfordii, P. trichocarpa, M. esculenta*, and *Cephalotus follicularis* gene families, a total of 951 genes appeared unique to *T. wilfordii* (Supplementary Fig. 5). Interestingly, Gene Ontology (GO) and Kyoto Encyclopedia of Genes and Genomes (KEGG) analyses found these *T. wilfordii*-specific genes were particularly enriched in the terms terpene synthase, oxidation-reduction process, and plant-pathogen interaction (Supplementary Tables 13 and 14).

The distribution of 4DTv (fourfold degenerate synonymous sites of the third codons) of all gene pairs found in each segment showed two peaks at approximately 0.09 and 0.48 in the *T. wilfordii* genome. The first peak at approximately 0.48 revealed the core eudicot γ triplication event, and the second peak at approximately 0.09 indicated that *T. wilfordii* underwent another whole-genome multiplication event after diverging from *P. trichocarpa* and *M. esculenta* (Fig. 1c). We further compared *T. wilfordii* genome and *Vitis vinifera* genome. 77% of *T. wilfordii* gene models are in syntenic blocks corresponding to one *V. vinifera* region, covering 83% of the *V. vinifera* gene space, among which 35% have three orthologous regions in *T. wilfordii*, 31% have two, and 15% have one (Supplementary Fig. 6). Intergenomic co-linearity analysis was consistent with both the γ-event and another, more specific WGT event for *T. wilfordii*, as indicated by a 1:3 syntenic relationship between *T. wilfordii* and *V. vinifera* (Fig. 1d, e). The recent WGT event was dated to approximately 21 ± 6 MYA, as indicated by the distribution of synonymous substitutions per synonymous site (*K*S) of syntenic genes in *T. wilfordii* (Supplementary Fig. 7). The recent WGT event occurred in the Paleogene (65–23.3 MYA) to Neogene (23.3-1.64 MYA) period, which may have enabled *T. wilfordii* to cope better with the markedly changed environment by functional redundancy, mutational robustness, increased evolution rate, and adaptation^37^.

Gene families that may be involved in terpenoid (e.g., triptolide in *T. wilfordii*) biosynthesis were identified in the fifteen reported plant species. The results showed that the copy number of these gene families varied among all examined plant species, with those encoding *DXS*, *GGPPS*, *TPS* and *CYP* exhibiting particularly strong variation (Supplementary Table 18). To further investigate the role of WGT events on triptolide biosynthesis, we carried out phylogenetic analysis of the gene families potentially involved in this pathway, specifically *Ks* calculations for each duplicated gene pair, including those from upstream isoprenoid metabolism (i.e., *ACAT*, *CMK*, *DXS*, *FPS*, *GPS*, *GGPPS*, *HDR*, *HDS*, *HMGR*, *HMGS*, *IDI*, *MCT*, *MVK*, *MVD*, *PMK*) and those more specific to triptolide (i.e., *CPS* and *MS*) (Supplementary Fig. 8 and Supplementary Table 19). We found that duplications of these genes were almost all generated by the recent WGT event (i.e., *ACAT*, *CMK*, *GPS*, *HDR*, *HMGS*, *IDI*, *MVK*, *MVD*, *PMK*, *CPS* and *MS*) (Supplementary Fig. 9), suggesting that the recent WGT event were important to the evolution of triptolide biosynthesis in *T. wilfordii*.

Notably it was found that the *TwCPS1* and *TwMS* genes already known to be involved in triptolide biosynthesis are adjacent to each other in the *T. wilfordii* genome (Supplementary Fig. 10), which is similar to previously identified biosynthetic gene clusters in other plant genomes^38–40^. However, while the relevant region on chromosome 21 also contains a *CYP* (TW023804.1) that exhibits a similar expression pattern, this is sufficiently distant, including a number of intervening genes clearly unrelated to triptolide biosynthesis, to leave its relevance unclear. Similarly, while there are several nearby transcription factors (TFs), as well as another CPS, these are unlikely to play a role in the production of triptolide.

### Integrated transcriptome and metabolome analysis

**Fig. 2:**
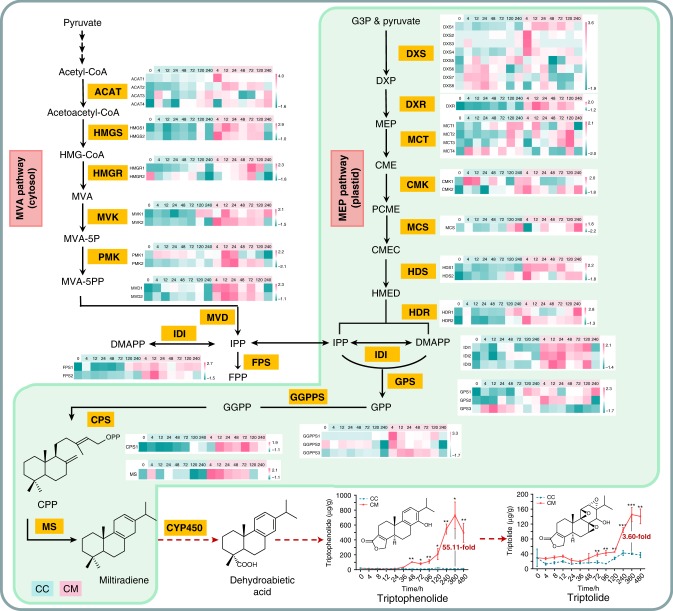
Comparative transcriptomic analysis of genes involved in the triptolide biosynthetic pathway. Suspension cells of *T. wilfordii* were treated with methyl jasmonate and DMSO. 0, 4, 12, 24, 48, 72, 120, 240 represent the time points of each sample, CC means the control group of cells, and CM means the group of MJ-treated cells. Heat maps of these genes were plotted using MeV software (version 4.9.0). Error bars, mean ± SD (*n* = 3 biologically independent samples; * *P* < 0.05, **P < 0.01, ***P < 0.001 by 2-sided Student’s *t* test). Source data are provided as a Source Data file.

To gain further insights into triptolide biosynthesis, as well as the organization and regulation of the triptolide pathway, suspension cells induced by methyl jasmonate (MeJA) and seven different tissues were used as the source of transcripts and metabolites (Supplementary Notes 4 and 5). The results showed that triptolide levels in MeJA-induced cells were 3.6-fold higher (relative to control cultures) after 360 h, while the levels of triptophenolide were even higher, 55-fold (Fig. 2). More detailed analysis was focused on a final set of 142 peaks with a mass ratio of 295–400, the expected range for diterpenoids (Supplementary Figs. 11-14 and Supplementary Note 7). For example, the content of these potentially triptolide-related metabolites was highest in the root bark (Supplementary Figs. 15 and 16). Data sets for these accumulated metabolites and gene expression (including all *CYPs*, *TwCPS1* and *TwMS*) were separately normalized, and correlation network analysis was used to establish gene-to-metabolite coregulation patterns^41^ in suspension cells and the various tissues, respectively. The Pearson correlation coefficient between each set of variables (either metabolite or gene) was calculated, including all conditions and time points (Supplementary Data 3 and 4). The correlation network was analyzed by Cytoscape (version 3.6.1), using a correlation coefficient >0.7 as the cutoff (Supplementary Figs. 19 and 20). The utility of the gene-to-metabolite network was verified by the observation that the key genes *TwCPS1* and *TwMS* were both strongly associated with triptolide and triptophenolide. Accordingly, we proceeded under the assumption that the CYP catalyzing the next step to form dehydroabietic acid should similarly be present among such strongly related genes. Indeed, a total of 97 *CYP* genes, of which 57 genes were reasonably well-expressed—i.e., with RPKM > 1 (Supplementary Fig. 21 and Supplementary Data 6)—were strongly associated with triptolide in the network. These then are potentially involved in triptolide biosynthesis.

### Identification of specific *CYP* genes in *T. wilfordii*

So far, triptolide is found only in *Tripterygium*, indicating that some of the CYPs involved in the biosynthesis of triptolide may be specific to *T. wilfordii*. In order to identify such species-specific CYPs, we annotated a total of 2335 *CYP* genes in *Arabidopsis thaliana, Daucus carota, Glycyrrhiza uralensis, Prunus persica, P. trichocarpa, Solanum lycopersicum, Salvia miltiorrhiza* and *T. wilfordii* (Supplementary Table 18). We constructed a phylogenetic tree from amino acid sequence alignment of all these CYPs and identified *T. wilfordii* specific CYPs using a cutoff of 55% identity, which indicates separate sub-family assignment^42^. This revealed 22 *T. wilfordii*-specific *CYP* genes (Supplementary Data 5). Interestingly, expression levels of six of these *CYPs* were significantly increased by MeJA induction, and most of these were highly expressed in root bark in which the terpene synthase genes were enriched and most of them were highly expressed (Supplementary Figs. 17, 18 and 22), leading to strong correlation with triptolide and/or triptophenolide.

### Functional identification of CYP728B70

**Fig. 3:**
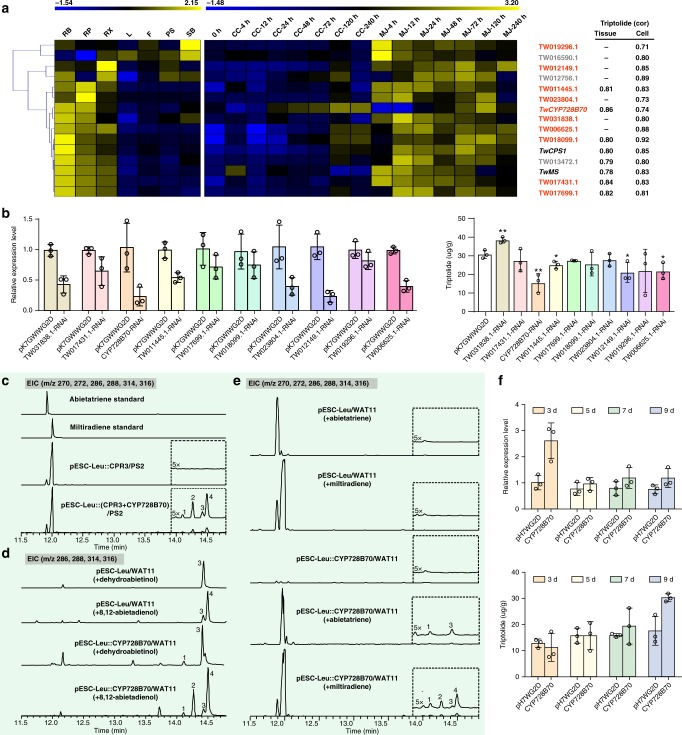
*CYP* gene screening and functional identification of CYP728B70. **a** Hierarchical clustering of RNA-Seq expression data after filtering by expression level. **b** Relative expression of 10 candidate CYPs and triptolide concentration in RNAi suspension cells. **c** Co-expression of CYP728B70 and TwCPR3 in miltiradiene engineering yeast. **d** In vivo transformation of authentic diterpene alcohol substrates to the corresponding alcohols and acids in cultures of yeast cells expressing CYP728B70. **e** In vivo transformation of authentic diterpene substrates to the corresponding alcohols and acids in cultures of yeast cells expressing CYP728B70. **f** Relative expression of *CYP728B70*, as well as triptolide concentration in CYP728B70-overexpressing suspension cells on the 3rd, 5th, 7th, 9th day. Error bars, mean ± SD (*n* = 3 biologically independent samples; *P < 0.05, **P < 0.01 by 2-sided Student’s *t* test). Source data underlying Figs. 3a, b, f are provided as a Source Data file.

Among *CYP* genes correlated with triptolide in the gene-to-metabolite network, as well as those specific to *T. wilfordii*, there were 13 found to express differentially between MeJA-induced and control cells at 4, 12, 24, 48, and 72 h, and/or express differentially between root bark and other tissues (e.g., flower, stem bark, peeled stem, and leaf), and also exhibit highly similar expression patterns as *TwCPS1* and *TwMS*, including high expression levels in root bark (Fig. 3a, Supplementary Fig. 18 and Supplementary Data 6). Among these candidates, TW016590.1 was already previously identified as *ent*-kaurene oxidase^43^, TW012756.1 was annotated as trans-cinnamate 4-monooxygenase, which is not likely to be involved in terpenoid biosynthesis, while TW017699.1 and TW013472.1 appeared to arise from a relatively recent tandem gene duplication event as their amino acid sequences are 97% identical (Supplementary Data 2), and we chose to only target TW017699.1 directly. Accordingly, we settled on a total of 10 candidates for the follow-up RNAi studies, which were carried out with suspension cell cultures to provide more direct evidence for a role in triptolide biosynthesis for these 10 candidates (Supplementary Table 20). In 4 RNAi-lines (CYP728B70, TW011445.1, TW012149.1, TW006625.1), the transcript levels of the targeted gene and triptolide accumulation were decreased compared to those in control cultures transformed with an empty vector (Fig. 3b). These four CYPs were co-expressed with the cytochrome P450 reductase 3 (CPR3)^43^ in yeast also engineered to produce miltiradiene, as previously described^44^. Gratifyingly, with CYP728B70, which was strongly associated with triptolide in the gene-metabolite network, this led to the formation of four compounds, dehydroabietic acid (abieta-8,11,13-trien-18-oic acid, **1**), miltiradienoic acid (abieta-8,12-dien-18-oic acid, **2**), dehydroabietinol (abieta-8,11,13-trien-18-ol, **3**) and miltiradienol (abieta-8,12-dien-18-ol, **4**), whose structures were identified after purification by ^1^H NMR and ^13^C NMR (Fig. 3c, Supplementary Figs. 24–32 and Supplementary Note 6). To further verify such activity, in vivo assays also were performed, that is, substrate feeding of cultures expressing CYP728B70 in the yeast WAT11 strain that also expresses an Arabidopsis CPR^45^. Compounds **1**-**4** were detected when feeding miltiradiene (abieta-8,12-diene), while only the derived compounds **1** and **3** were detected when feeding abieta-8,11,13-triene. Similarly, compounds **1** and **2** were detected when feeding compound **4** (miltiradienol), while only compound **1** was detected when feeding compound **3** (dehydroabietinol) (Fig. 3d, e). Given that aromatization of the miltiradiene C ring to form abietatriene most likely occurs spontaneously, these results indicate that TwCYP728B70 catalyzes consecutive oxidations at C-18 of miltiradiene or abietatriene to form the corresponding alcohol and acid derivatives. Although the expected aldehyde intermediate is not observed, this almost certainly also is formed between the alcohol and acid final products.

To further explore the role of CYP728B70 in triptolide biosynthesis, overexpression was employed. This led to a significant increase in transcript level, albeit only by ~1.6-fold compared with the control cultures transformed with the empty vector, on the 3rd day, and it then decreased but was still slightly higher than in the control cultures at later time points. Nevertheless, elevated levels of triptolide in the overexpression line relative to those in the control cultures were evident on the 9th day, reaching just over 30.5 μg g^−1^ in the overexpression line, representing an ~70% increase (Fig. 3f). This not only further supports a role for CYP720B70 in triptolide biosynthesis, but also showcases its potential utility for improving production of this valuable diterpenoid in *T. wilfordii*.

To explore the potential functions of the other 9 candidate CYPs, in vitro enzymatic activity assays were performed using the available dehydroabietic acid, triptinin B, triptophenolide and triptoquinonide, all of which are likely intermediates in triptolide biosynthesis (Supplementary Note 7). However, no new compounds were observed, indicating that these CYPs could not catalyze transformation of these intermediates, but does not rule out a role for them in triptolide biosynthesis (i.e., in mediating reactions involving other intermediates) (Supplementary Fig. 33).

## Discussion

Triptolide, a structurally complex phenolic diterpene triepoxide of *T. wilfordii*, has potent antitumor and immunosuppressive activities. Research focusing on its biosynthetic pathway has been stymied, in part, by the absence of a comprehensive genetic accounting. The high-quality reference-grade genome of *T. wilfordii* reported here thus provides a valuable genomic resource for investigation of triptolide biosynthesis, and is, to the best of our knowledge, the first genomic sequence for a plant of the order Celastrales. Accordingly, it further represents a cornerstone for evolutionary phylogenomic studies of not only *T. wilfordii*, but the Celastrales order more generally.

Previous investigations of triptolide biosynthesis relied on transcriptome data, and only identified the relevant diterpene synthases. Herein, we integrated genomic, transcriptomic, and metabolomic data to map a gene-to-metabolite network, screening out 57 *CYP* genes which were potentially involved in triptolide biosynthesis. Then we combined the co-expression patterns, tissue-specificity and inducibility of these candidate genes as well as *T. wilfordii-*specific *CYP* genes for further investigations, resulting in a total of 10 candidates. Through the above analysis, we identified 4 *CYP* genes involved in triptolide biosynthesis, as RNAi knock-down of these led to decreased accumulation of this natural product. Moreover, we successfully characterized the role of CYP728B70, which is responsible for oxidation of C-18 from a methyl to the acid moiety of dehydroabietic acid, in triptolide biosynthesis. As demonstrated here by substrate feeding studies, CYP728B70 exhibits multiple/sequential reactivity in carrying out this transformation in triptolide biosynthesis. Consistent with the limited accumulation of triptolide in *T. wilfordii*, *CYP728B70* exhibits low transcript levels, although it is highest in the root bark where this diterpenoid is primarily found. Notably, overexpression of *CYP728B70* in plant cell cultures led to a significant (~70%) increase in triptolide levels relative to control cultures, indicating that CYP728B70 activity limits triptolide biosynthesis, and demonstrating the utility of the results reported here for improving the yield of this potential pharmaceutical agent.

Perhaps not surprisingly given that CYPs form the largest family of enzymes, there are still CYP families that are completely uncharacterized. Indeed, this included the CYP728 family, which was first observed in *Amborella*, although it has been lost in *Arabidopsis* (Supplementary Fig. 35), and no function has been previously reported for any member of this CYP family^32^. Hence, CYP728B70 appears to be the only one member of this CYP family whose catalytic activity has been identified, and its role in diterpenoid metabolism immediately suggests similar function for other family members. Also, the stepwise carboxylation reaction observed for CYP728B70 is catalytically highly similar to the CYP720B family in conifers^46,47^ that even forms some common products, thus showcasing an intriguing case of independent evolution of these functions in distant plant species. In addition, the other 3 *CYP* genes that affect the biosynthesis of triptolide in the results of RNAi experiment are from *T. wilfordii*-specific CYP (sub-)families. Although we have not identified their catalytic functions, such biochemical characterization will then provide similar insight.

Although some steps in the triptolide biosynthetic pathway still remain unknown, we have provided many promising candidates, including *CYP* genes and metabolites that might constitute yet-unknown intermediates or side products from triptolide biosynthesis. We have also provided many potential TFs that are most likely involved in the regulation of the biosynthesis of triptolide (Supplementary Fig. 34, Supplementary Data 8–13 and Supplementary Note 8). Of particular note here is the identification of roles for CYPs from (sub-)families that are specific to *T. wilfordii*, as much of the reported work in such characterization of CYP function in plant natural products biosynthesis has to some extent relied on homology at least at the family, if not sub-family, level.

**Fig. 4:**
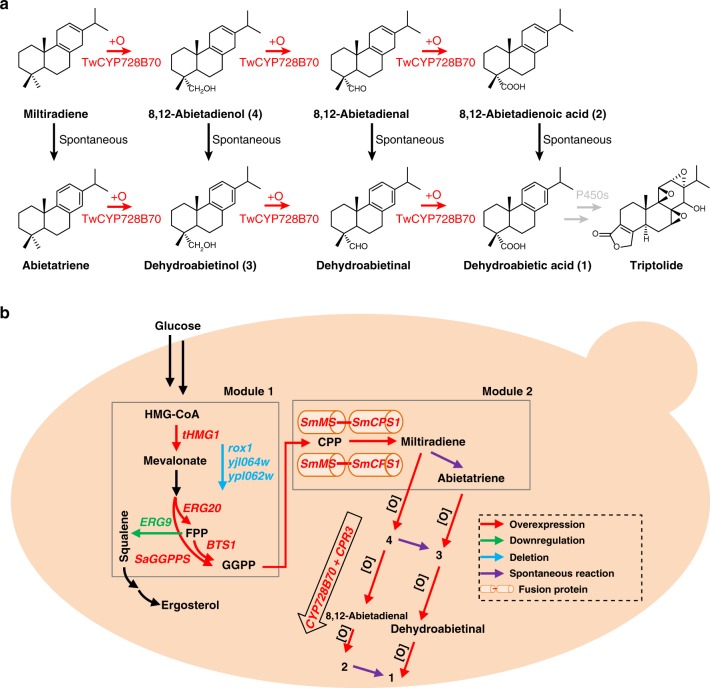
Analysis of triptolide pathway and metabolic engineering. **a** Three reactions presumably catalyzed by CYP728B70 to convert miltiradiene to dehydroabietic acid. **b** Establishing the metabolic pathways in yeast for production of diterpene alcohols and acids. In module 1, *rox1*, *ypl062w*, *yjl06w4* were knocked out, and *ERG9* was down-regulated, and *tHMG1*, *ERG20*, *BTS1*, *SaGGPPS* were overexpressed to improve the production of GGPP. In module 2, double SmMS-SmCPS1 fusion modules were introduced into the yeast chromosome for the production of miltiradiene. Co-expression of TwCYP728B70 and TwCPR3 leads to the observed production of the derived alcohols and acids.

In addition to our identification of the role of CYP728B70, we used this to engineer yeast for the production of dehydroabietic acid (Fig. 4). This lays the foundation for identification of genes encoding subsequently acting enzymes, and will be invaluable in future investigations of triptolide biosynthesis.

In conclusion, while it is difficult to resolve the biosynthetic pathways of the complex natural products in non-model systems, we have demonstrated here the utility of the multi-omics data, as well as co-expression patterns, tissue-specificity, and inducibility of candidate genes will contribute for such investigations. Interestingly, while genome sequencing found pairing of the genes for the consecutively acting *TwCPS1* and *TwMS* can initiate triptolide biosynthesis, these do not appear to have be co-clustered with genes for later acting enzymes. Nevertheless, the genome sequence reported here provides a comprehensive genetic inventory, which was coupled with the observation that triptolide production is tissue-specific and affected by elicitors, much as found with other plant natural products^48^, to generate gene-to-metabolite networks that can be productively mined. Notably, this approach has led to identification of four relevant CYPs, with the characterized function of CYP728B70 further providing insight into this previously enigmatic CYP family, as well as demonstrating the utility of this approach to increasing the yield of this promising pharmaceutical agent.

## Methods

### Genome sequencing and assembly

A *T. wilfordii* cultivar was used for sequencing (Supplementary Note 1). Genomic DNA was extracted from leaves of *T. wilfordii* using the DNAsecure Plant Kit (TIANGEN) and broken into random fragments. Short-reads libraries were constructed according to the manufacturer’s instructions (Illumina, San Diego, CA) and then sequenced on Illumina Hiseq X-ten. For long-read DNA sequencing, 60 kb Single Molecule Real Time (SMRT) long-read library were sequenced on the PacBio Sequel platform (75.79 Gb data, 207-fold coverage of the genome). For 10X Genomics sequencing, a total of 119.75 Gb (327-fold coverage of the genome) data were sequenced on the Illumina Hiseq X-Ten (Supplementary Table 2).

De novo assembly of the long reads from the PacBio SMRT Sequencer was performed using FALCON (https://github.com/PacificBiosciences/FALCON/)^49^. To obtain enough corrected reads, the longest coverage of subreads were firstly selected as seed reads to correct sequence errors. Then, error-corrected reads were aligned to each other and assembled into genomic contigs using FALCON with the following parameters: length_cutoff_pr = 10,000, max_diff = 95, and max_cov = 105. Then, genomic contigs were polished using Quiver^50^, which yielded an assembly with a contig N50 size of 4.36 Mb. The total length of this assembly version was 348.38 Mb. Then, we used BWA-MEM to align the 10X Genomics data to the assembly using default settings^51^. Scaffolding was performed by FragScaff with the barcoded sequencing reads^52^. Last, Pilon^53^ was used to perform error correction based on the Illumina sequences, generating a genome with a scaffold N50 size of 6.48 Mb. The total length of this assembly version was 349.91 Mb. Subsequently, the Hi-C sequencing data were aligned to the assembled scaffolds by BWA-mem^50^ and the scaffolds were clustered onto chromosomes with LACHESIS (http://shendurelab.github.io/LACHESIS/), the final genome was 348.53 Mb and the contig and scaffold N50 were 4.36 Mb and 13.52 Mb, respectively (Supplementary Tables 3–7 and Supplementary Note 1).

### Genome annotation

A total of 52.36% repeat sequences in the genome were annotated. Among them, TEs were searched by combining de novo-based and homology-based approaches using RepeatModeler (http://www.repeatmasker.org/RepeatModeler/), LTR_FINDER (http://tlife.fudan.edu.cn/ltr_finder/), RepeatScout (http://www.repeatmasker.org/), RepeatMasker (version 3.3.0) (http://www.repeatmasker.org/), and RepeatProteinMask (http://www.repeatmasker.org/). Tandem repeats were detected using Tandem Repeats Finder (TRF)^54^ (Supplementary Fig. 2 and Supplementary Table 10). Gene structures were predicted with a combination of homology-based prediction, de novo prediction and transcriptome-based prediction in the genome (Supplementary Fig. 3, Supplementary Tables 8 and 9). We then generated functional assignments of the *T. wilfordii* genes with BLAST in public protein databases, including SwissProt (https://web.expasy.org/docs/swiss-prot_guideline.html), NR, InterPro (V32.0)^55^, Pfam (V27.0)^56^ and KEGG (https://www.kegg.jp/). Finally, 99.6% of all genes in the genome were predicted to be functional (Supplementary Table 11). Noncoding RNA was predicted using de novo and homology search methods (Supplementary Table 12 and Supplementary Note 2).

### Genome evolution

We conducted expansion and contraction analysis using the CAFÉ program^57^ (Supplementary Table 15) and identified the positively selected genes in *T. wilfordii* using MUSCLE^58^ (Supplementary Tables 16 and 17). To identify the WGD events in the *T. wilfordii* genome, we used McscanX^59^ to determine syntenic blocks (regions with at least five genes) and calculated 4DTv for all gene pairs found in each syntenic segment. The synonymous substitution rate (*Ks*) values of *T. wilfordii* syntenic block genes were calculated with the codeml program of the PAML^60^ package. We performed synteny analysis on *T. wilfordii* and *V. vinifera* to confirm that *T. wilfordii* had undergone another WGT event.

### Evolution of triptolide biosynthesis genes

To investigate the genes involved in triptolide biosynthesis, we first retrieved protein sequences from *Arabidopsis thaliana* genes, including *ACAT*, *CMK, DXR*, *DXS*, *FPS*, *GGPPS*, *GPS*, *HDR*, *HDS*, *HMGR*, *HMGS*, *IDI*, *MCT*, *MCS*, *MVK*, *MVD,* and *PMK* from the NCBI database. The CPS and MS protein sequences in *T. wilfordii* were previously cloned^28,31^. Then, using these homologs as queries, we identified the candidates in *T. wilfordii* and 13 other plant species, including *Carica papaya, Daucus carota, Dimocarpus longan, Glycine max, Gossypium raimondii, Glycyrrhiza uralensis, Oryza sativa, Prunus persica, Populus trichocarpa, Solanum lycopersicum, Salvia miltiorrhiza, Theobroma cacao, Vitis vinifera* using BLASTP with an E-value cutoff of 1e^−5^. The aligned hits with at least 50% coverage of seed protein sequences and >50% protein sequence identity were selected as homologs. Then, the domains of these homologs were predicted by PFAM (http://pfam.xfam.org/). Only genes that had the same protein domain were considered to be homologs. The TPS and CYP450 genes were predicted using hmmsearch^61^ in conjunction with the TPS hmm model (PF01397 and PF03936) and CYP450 hmm model (PF00067) from Pfam, respectively. We constructed phylogenetic trees of each identified triptolide biosynthetic gene family from *T. wilfordii* and *Arabidopsis thaliana*. Amino acid sequence alignments of the identified triptolide biosynthetic genes from *T. wilfordii* and *Arabidopsis thaliana* were performed using MUSCLE^58^, and the alignment data were used to construct phylogenetic trees using RAxML^62^ with the maximum likelihood method. The timing of the divergence of duplicates in each triptolide biosynthesis gene was estimated based on the calculation of the synonymous substitution rate (Ks) using the codeml program of the PAML^60^ package. The calculated *Ks* value was then converted to the divergence time according to *T* = *Ks*/2r, where r represents a substitution rate of 6.5 × 10^−9^ mutations per site per year for eudicots (Supplementary Table 19).These gene pairs were not adjacent in the distribution of the genome (Supplementary Data 2).

### Suspension cell elicitation and sample preparation

It has previously been shown that the application of the plant defence signaling molecule MeJA could increase terpenoid production and affect the transcript levels of the genes involved in the related pathways^28,63^. Cell samples were harvested at 0, 4, 8, 12, 24, 36, 48, 72, 96, 120, 240, 360, and 480 h after the addition of MeJA and DMSO. Solid samples were homogenized by Mixer Mill MM 400 of Retsch (Retsch GmbH, 42781 Haan, Germany) and stored at −80 °C for at least 4 h prior to freeze drying for 48 h (Alpha 1-2 LDplus, Germany). For each sample, 60 mg aliquots were soaked in 1.5 mL of 80% (v/v) methanol overnight at room temperature and then dissolved in an ultrasonic water bath for 60 min. The supernatant was filtered through a 0.22-μm membrane filter (FitMax Syringe Filter, 13 mm 0.22 μm) for further analysis.

### UPLC/Q-TOF MS analysis

All analyses were performed on a UPLC/Q-TOF MS system (Waters Corp., Milford, MA, USA). The UPLC separation was performed using a Waters ACQUITY UPLC HSS T3 analytical column (2.1 mm × 100 mm, 1.8 μm) kept at 40 °C and a Waters Acquity UPLC^TM^ I-Class system. The mobile phase was pumped with a mixture of 0.1% (v/v) aqueous acetic acid (A) and acetonitrile (B) at a flow rate of 0.5 mL min^−1^. Gradient elution was performed as follows: 0 min at 30% B, 6 min at 45% B, 18 min at 60% B, and 23 min at 90% B. The TOF MS experiments were performed on the Xevo G2-S QTOF MS system. The experiment was performed in ESI (+) ionization modes, and the data acquisition modes were MSE continuum. The capillary voltage was 0.5 KV, and the cone voltage was 40 V. The source and desolvation temperatures were 100 °C and 450 °C, respectively. The desolvation gas flow was 900 L h^−1^. The ramp collision energy was set as 20–40 eV for the high-energy scans. The MS range of data acquisition was 50–1500 Da. A lock spray with leucine enkephalin (200 pg μL^−1^, 10 μL min^−1^) was used as the reference (m/z 556.2771 ESI (+)) to maintain the mass accuracy.

### Data analysis of metabolic and transcriptional profiles

All mass spectral data were imported into Progenesis QI for data processing, then were grouped and exported to Ezinfo for principal component analysis (PCA)^64^. PCA is a very useful statistical method for defining the dimensions of large data sets and identifying significant signals^65^. The data set was log normalized, and PCA and OPLS-DA analyses were performed to determine overall differences in metabolites. An ANOVA with a significance level of *P* < 0.01 and max-fold > 2 was subsequently performed on the doubly filtered peaks to identify metabolites that did not change significantly in response to MeJA treatment. Furthermore, peaks with no fragments were removed.

We selected metabolite peaks and genes including all CYP, CPS1 and MS for network regulation analysis. Both data sets of accumulation of metabolic and gene expression were normalized separately, and correlation network analysis was used to establish gene-to-metabolite coregulation patterns^41^. The Pearson correlation coefficient was calculated by the PCC method in the R platform between each set of variables (either metabolite or gene) across the profiles, and significant positive correlations with *p*-value < 0.05 were detected between genes and metabolites. The correlation network was analyzed by the Cytoscape software (version 3.6.1)^66^.

### Construction of miltiradiene-producing yeast strain

To construct a miltiradiene-producing strain, the corresponding biosynthetic pathway was introduced into yeast. First, the CRISPR/Cas9 system was applied to improve the MVA pathway flux by knocking out three transcriptional regulators (*rox1*, *ypl062w* and *yjl06w4*) and knocking down *erg9* in the yeast BY-T20 strain (BY4742, *ΔTrp1, Trp1::His3-P*_*PGK1*_*-BTS1/ERG20-T*_*ADH1*_*-P*_*TDH3*_*-SaGGPPS-T*_*TPI1*_*-P*_*TEF1*_*-tHMG1-T*_*CYC1*_), generating a GGPP-producing yeast strain named BY-HZ16^44,67–69^.

The fusion of *SmCPS1* and *SmMS* from *Salvia miltiorrhiza* was reported to possess great potential to synthesize miltiradiene via GGPP^70^. The single or double *SmMS-SmCPS1* fusion module was cloned and integrated into the yeast chromosome by the M2S integration method^71^. Briefly, *SmMS-SmCPS1* was amplified with the addition of a *Bsa*I digestion site and ligated with head-to-head promoters (*pTDH3-pADH1*) into the bi-terminator vector T1-(*tTPI1-tPGI1*), resulting in the plasmids T1-(*SmMS-SmCPS1*) and T1-(*SmMS-SmCPS1*)-(*SmMS-SmCPS1*). Two terminators were inserted into the scaffold plasmid, with dedicated homologous arms (L1 and L2) lying on both sides. The integration site *YPRCΔ15* was chosen as the target locus, and *Ura3* was chosen as the selection marker. Each expression cassette with designed to have homologous arms (primers: L1-F/L2-R) was amplified individually. The selection marker module and integration homologous arm module (15Site1-Ura3-L1 and L2-15site2) were also amplified. All the amplified fragments were used to co-transform BY-HZ16 for assembly and integration, and transformants were selected on synthetic dropin medium-Ura-His (SD-Ura-His) containing 20 g L^−1^ glucose and 18 g L^−1^ agar. Positive transformants were verified by sequencing, yielding the strains PS1 and PS2 (Supplementary Fig. 23). All strains are listed in Supplementary Table 21. All primers used in vector construction are listed in Supplementary Data 7.

### *CYP* screening based on the miltiradiene-producing strain

The gene-to-metabolite network analysis and specific-gene analysis in *T. wilfordii* were performed, and 10 highly expressed *CYP* genes were chosen as candidates for investigation. The RNAi results showed that 4 CYPs were most likely to regulate triptolide biosynthesis and were then selected to react with abietane-type diene olefin miltiradiene. First, to simplify the fermentation procedure, the constitutive promoters *pPGK1-pTEF2* were cloned into the *BamH*I and *Not*I sites of the pESC-Leu vector to replace the inducible promoters *pGAL1-pGAL10*, yielding the plasmid Leu-PT. Second, cytochrome P450 reductase 3 (CPR3) was inserted into the *Not*I site of the plasmid Leu-PT according to the pEASY-Uni Seamless Cloning and Assembly Kit (TransGen Biotech, Beijing, China), resulting in the plasmid Leu-PT-CPR3. Then, each *CYP* gene was introduced into the *BamH*I site of the plasmid Leu-PT-CPR3. All the resulting CYP expression plasmids were individually introduced into strain PS2 following the user manual of the Frozen-EZ Yeast Transformation II KitTM (Zymo Research, USA) for product identification. All primers used for *CYP* gene screening are listed in Supplementary Data 7.

Three colonies were picked for each genotype and used to inoculate 5 mL of synthetic dropin medium -Ura-His-Leu (SD-Ura-His-Leu) containing 20 g L^−1^ glucose. The cells were grown in a shaker at 30 °C and 230 rpm for 48 h, after which the resulting seed cultures were transferred into fresh medium at a ratio of 1:50 and fermented under the same conditions for 3 days. Yeast cells were lysed using a nano homogenizer (AH-1500, ATS Engineering Limited, Canada) and then extracted twice with an equal volume of ethyl acetate. Anhydrous sodium sulfate was added to remove residual water, and the combined organic phases were dried and methylated with (trimethylsilyl)diazomethane (Aladdin Industrial Inc., Shanghai, China)^43,72^. The methylated samples were re-dried and then dissolved in 100 μL of ethyl acetate for gas chromatography-mass spectrometry (GC-MS) using a Thermo TRACE 1310/TSQ 8000 gas chromatograph equipped with a TG-5 MS (30 m × 0.25 mm × 0.25 μm) capillary column. The GC conditions were as follows: the sample (1 μL) was injected in split mode (20:1) at 250 °C under a He flow rate of 1 mL min^−1^, the GC oven temperature was programmed to rise from an initial 40 °C at 20 °C min^−1^ to 200 °C and at 15 °C min^−1^ to 250 °C, then to 270 °C at 1.5 °C min^−1^. The ion trap heating temperature was 250 °C. The electron energy was 70 eV. Spectra were recorded in the range of 40–500 m/z.

### In vivo assays for TwCYP728B70 activity

The pESC-Leu::(CPR3 + TwCYP728B70) construct was transformed into the yeast strain WAT11, which enables the catalytic activity of plant CYPs by expressing an Arabidopsis CPR^45^. Transformants were selected on synthetic dropin medium SD-Leu plates containing 20 g L^−1^ glucose. The cells were grown in a shaker at 30 °C and 230 rpm for 48 h, then transferred into 50 mL of fresh medium at a ratio of 1:50 and fermented under the same conditions for 12 h. To confirm engineered yeast strains for oxidative transformation of diterpenoid substrates, miltiradiene, abietatriene, 8,12-abietadienol, or dehydroabietinol (in methanol) was added to the yeast cultures to final concentrations of 100 μM and fermented for another 48 h. Yeast cells were lysed, extracted twice with an equal volume of ethyl acetate, and methylated before GC-MS analysis, as described above.

### RNAi of the candidate *CYP* genes in *T. wilfordii*

The fragments of these 10 candidate *CYP* genes were amplified using Phusion® High-Fidelity DNA Polymerase (New England Biolabs, USA) and inserted into the RNAi vector pK7GWIWG2D according to the Gateway procedure (Invitrogen, USA), and the resulting vectors were verified by complete sequencing.

Suspension cells in the logarithmic growth phase were chose and precultured on Murashige and Skoog (MS) solid medium containing 0.5 mg L^−1^ 2,4-D, 0.1 mg L^−1^ KT, 0.5 mg L^−1^ IBA and 30 g L^−1^ sucrose (pH = 5.8) for 7 days. Then, the recombinant plasmid DNA mixed with Au microparticles were transformed into the suspension cells through bombardment using a biolistic gene gun (PDS 1000/He, Bio-Rad). Each transformation was carried out two times. The bombarded suspension cells were cultured for another 7 days before harvesting for qRT-PCR and UPLC analysis^73^.

### Overexpression of *TwCYP728B70*

Vector pH7WG2D (Invitrogen) was used to overexpress *TwCYP728B70* in suspension cells following the protocol mentioned above. The resulting recombinant cells were harvested for qRT-PCR and UPLC analysis after being cultured for 3, 5, 7, and 9 days^73^.

### Heterologous expression of CYP in yeast and in vitro assays

Each *CYP* gene was inserted into the *BamH*I site of the pESC-Leu vector according to the pEASY-Uni Seamless Cloning and Assembly Kit. The pESC-Leu::CYP construct was verified by complete gene sequencing, which was transformed into the yeast strain WAT11. The cells were grown first in 100 mL of SD-Leu liquid medium with 20 g L^−1^ glucose in a shaker at 30 °C and 230 rpm to an OD_600_ of 2–3. Cells were centrifuged and resuspended in 200 mL of yeast peptone galactose (YPL) induction medium (10 g L^−1^ yeast extract, 20 g L^−1^ bactopeptone, and 20 g L^−1^ galactose) and grown for 12 h at 30 °C to induce recombinant protein expression. Microsomes were prepared based on the reported method with some modifications^74,75^. Briefly, the induced cells were centrifuged (1914 × *g*, 4 °C, 5 min) and resuspended in 20 mL of TEK buffer (50 mM Tris-HCl, pH 7.4, 1 mM EDTA, 0.1 M KCl), then left at room temperature for 5 min. Cells were centrifuged again (1,914 × *g*, 4 °C, 5 min) and resuspended in 50 mL of TESB buffer (50 mM Tris-HCl, pH 7.4, 1 mM EDTA, 0.6 M sorbitol), then left on ice for 10 min. The cell suspension was lysed for 7 min at 4 °C and 12,000 psi using a nano homogenize machine (AH-1500, ATS Engineering Limited, Canada), then centrifuged (17,266 × *g*, 4 °C, 15 min) to collect the supernatant. NaCl (final concentration of 0.15 M) and polyethylene glycol (PEG)−4000 (final concentration of 0.1 g mL^−1^) were added to the supernatant, and left on ice for 15 min. The microsomal fractions were collected by centrifugation (17,266 × *g*, 4 °C, 15 min), and resuspended in TEG buffer (50 mM Tris-HCl, pH 7.4, 1 mM EDTA, 20% (v/v) glycerol), which can be kept frozen at −80 °C for months.

In vitro enzymatic activity assays were carried out on a shaking incubator (150 rpm) at 30 °C for 4 h in 500 μL of 100 mM Tris-HCl, pH 7.5, containing 0.5 mg of total microsomal proteins and 500 μM NADPH, along with a regenerating system (consisting of 5 μM FAD, 5 μM FMN, 5 mM glucose-6-phosphate, 1 unit mL^−1^ glucose-6-phosphate dehydrogenase), and 100 μM substrates. Reactions were stopped by the addition of 500 μL of methanol and used for UPLC analysis. Negative control reactions were carried out with microsomal preparations from recombinant yeast transformed with empty pESC-Leu.

### Fermentation

To engineer yeast for the production of intermediates involved in triptolide biosynthesis and obtain enough compound for structural characterization, the strain PS2 containing the plasmid Leu-PT-CPR3::TwCYP728B70 was used to inoculate 50 mL of SD-Ura-His-Leu medium in a 250 mL shake flask at 30 °C and 230 rpm for 24 h. The entire culture volume was transferred into 500 mL of fresh seed medium and incubated for 24 h, then transferred into 2 mL of fresh seed medium and incubated for another 24 h. The seed medium was then used to inoculate 8 L of fermentation medium in a New Brunswick BioFlo/CelliGen 115 bioreactor (Eppendorf, Germany) with a maximal working volume of 14 L.

The fermentation was performed at 30 °C. During fermentation, the pH was maintained at 5.0 with the automatic addition of ammonium hydroxide, the agitation rate was kept between 200 and 600 rpm, and the dissolved oxygen was kept above 40%. Concentrated glucose solution (40%, wt/vol) was fed periodically to keep the glucose concentration above 1.0 g L^−1^. Additionally SD-Ura-His-Leu medium was fed after the initial 30 h of fermentation. The culture was then harvested by extraction after 90 h of total fermentation time.

Yeast cells were concentrated to 1 L, then lysed using the nano homogenizer and then extracted ten times with an equal volume of ethyl acetate. Anhydrous sodium sulfate was added to remove residual water, and the organic fractions were pooled and dried using a Nitrogen Evaporator (Baojingkeji, Henan, China). The yellow oily liquid (15.5 g) was chromatographed on LiChroprep® Si60 (40–63 μm, Merck, MA, USA) with a stepwise gradient of petroleum ether-hexane (v/v, 10:1) to obtain the fraction (~500 mL). The fraction was re-dried and then dissolved in 500 μL of acetonitrile. Preparative HPLC was performed on Agilent 1260 Infinity High-Performance Liquid Chromatography System with a Shim-pack GIST C18 (250 × 4.6 mml.D., 5 μm, SHIMADZU, Kyoto, Japan). The mobile phase, consisting of a mixture of water (A) and acetonitrile (B), was pumped at a flow rate of 1 ml min^−1^ and the eluate was monitored at 200 nm. The gradient elution was programmed as follows: 0 min at 65% B, 50 min at 65% B. The injection volume was 20 μL.

### Reporting summary

Further information on research design is available in the Nature Research Reporting Summary linked to this article.

## Supplementary information

Supplementary Information

Peer Review File

Reporting Summary

Description of Additional Supplementary Files

Supplementary Data 1-13

## Data Availability

The data supporting the findings of this work are available within the paper and its Supplementary Information files. A reporting summary for this Article is available as a Supplementary Information file. The data sets generated and analyzed during this study are available from the corresponding author upon request. The genome sequence data and transcriptome sequence data for *T. wilfordii* have been deposited under NCBI BioProject number PRJNA542587. The source data underlying Figs. 1, 2, 3a, 3b, and 3f are provided as a Source Data file.

## Source data

Source Data
