# Author Correction: Genome of *Tripterygium wilfordii* and identification of cytochrome P450 involved in triptolide biosynthesis

**DOI:** 10.1038/s41467-020-19253-3

**Published:** 2020-10-15

**Authors:** Lichan Tu, Ping Su, Zhongren Zhang, Linhui Gao, Jiadian Wang, Tianyuan Hu, Jiawei Zhou, Yifeng Zhang, Yujun Zhao, Yuan Liu, Yadi Song, Yuru Tong, Yun Lu, Jian Yang, Cao Xu, Meirong Jia, Reuben J. Peters, Luqi Huang, Wei Gao

**Affiliations:** 1 School of Traditional Chinese Medicine, Capital Medical University, Beijing, China; 2 School of Pharmaceutical Sciences, Capital Medical University, Beijing, China; 3 State Key Laboratory Breeding Base of Dao-di Herbs, National Resource Center for Chinese Materia Medica, China Academy of Chinese Medical Sciences, Beijing, China; 4 Novogene Bioinformatics Institute, Beijing, China; 5 University of Chinese Academy of Sciences, Beijing, China; 6 Roy J. Carver Department of Biochemistry, Biophysics & Molecular Biology, Iowa State University, Ames, IA USA; 7 Advanced Innovation Center for Human Brain Protection, Capital Medical University, Beijing, China; 8 Present Address: Department of Chemistry, The Scripps Research Institute, Jupiter, Florida USA

Correction to: *Nature Communications* 10.1038/s41467-020-14776-1, published online 20 February 2020.

In the original version of this Article, the accession number of *Tripterygium wilfordii* genome assembly was not included in the “Data availability” statement. The “Data availability” statement has been amended from “The genome sequence data and transcriptome sequence data for *T. wilfordii* have been deposited under NCBI BioProject number PRJNA542587” to “The genome sequence data and transcriptome sequence data for *T. wilfordii* have been deposited under NCBI BioProject number PRJNA542587 and the final assembly is available at GenBank under the accession number JAAARO000000000.”

This has been corrected in both the PDF and HTML versions of the Article.

