# Supplementary material for: Genome of Tripterygium wilfordii and identification of cytochrome P450 involved in triptolide biosynthesis
Source: Nat Commun. 2020 Feb 20;11:971. doi: 10.1038/s41467-020-14776-1 (PMC7033203; doi:10.1038/s41467-020-14776-1)
Supplement: Supplementary file 1 — Supplementary Information [file 41467_2020_14776_MOESM1_ESM.pdf]

Genome of *Tripterygium wilfordii* and identification of cytochrome P450 involved in triptolide biosynthesis

Tu *et al*.

Supplementary Note 1

Sample information

The *Tripterygium wilfordii* cultivar used for sequencing was collected from Taining County, Fujian Province, China, which is considered to be the highest quality production site of *T. wilfordii*. Fresh and healthy leaves were harvested, and external contaminants were removed by washing with ultrapure water three times. Then, the leaves were immediately frozen in liquid nitrogen and stored at -80 °C prior to DNA extraction.

Genome sequencing

Genomic DNA was extracted from leaves of *T. wilfordii* using the DNAsecure Plant Kit (TIANGEN) and broken into random fragments. DNA sequencing libraries were constructed according to the standard Illumina library preparation protocols. Paired-end library with insert size of 350 bp was constructed according to the manufacturer's instructions (Illumina, San Diego, CA). The constructed library was sequenced on Illumina Hiseq X-ten. After filtering out the adapter sequences and the low-quality and duplicated reads, a total of 39.83 Gb of data were retained for the assembly.

For 10X Genomics libraries, approximately 1 ng of input DNA with 50 kb length was used for the GEM reaction procedure during PCR, and 16-bp barcodes were introduced into droplets. Then, the droplets were fractured following the purification of the intermediate DNA library. The libraries were finally sequenced on the Illumina Hiseq X-Ten. Finally, a total of 119.75 Gb (327-fold coverage of the genome) data were retained (Supplementary Table 2).

For pacbio libraries, at least 10 μg of sheared DNA was required. The SMRTbell

template preparation involved DNA concentration, damage repair, end repair, ligation of hairpin adapters, and template purification. SMRT Bell libraries with an insert size of 60 kb were constructed and then sequenced on the PacBio Sequel platform (Pacific Biosciences, USA) using the P6 polymerase/C4 chemistry combination, based on the manufacturer's procedure (Pacific Biosciences, CA, USA). A total of 75.79 Gb of (207-fold coverage of whole genome) data were retained (Supplementary Table 2).

DNA from young leaves of the same *T. wilfordii* plant was used as starting material for the Hi-C library. Formaldehyde was used for fixing chromatin. The leaf cells were lysed and *Hind*III endonuclease was used for digesting the fixed chromatin. The 5' overhangs of the DNA were recovered with biotin-labeled nucleotides and the resulting blunt ends were ligated to each other using DNA ligase. Proteins were removed with protease to release the DNA molecules from the crosslinks. The purified DNA was sheared into 350-bp fragments and ligated to adaptors[1]. The fragments labeled with biotin were extracted using streptavidin beads and after PCR enrichment, the libraries were sequenced on Illumina HiSeq X instrument.

Estimation of genome size using k-mer analysis

Genome size can be estimated by analysing the *K*-mer frequency. We selected 39.83 Gb pair-end reads (350 bp) and generated 17-mer frequency information based on the k-mer analysis. The distribution of the 17-mer depends on the characteristics of the genome and follows a Poisson's distribution. We estimated that the genome size is 365.95 Mb (Supplementary Table 1, Supplementary Fig. 1).

Genome assembly

De novo assembly of the long reads from the PacBio SMRT Sequencer was performed using FALCON (https://github.com/PacificBiosciences/FALCON/)[2]. To obtain enough corrected reads, the longest coverage of subreads were firstly selected

as seed reads to correct sequence errors. Then, error-corrected reads were aligned to each other and assembled into genomic contigs using FALCON with the following parameters: length_cutoff_pr = 10,000, max_diff = 95, and max_cov = 105. Then, genomic contigs were polished using Quiver[3], which yielded an assembly with a contig N50 size of 4.36 Mb. The total length of this assembly version was 348.38 Mb. Then, we used BWA-MEM to align the 10X Genomics data to the assembly using default settings[4]. Scaffolding was performed by FragScaff with the barcoded sequencing reads[5]. Last, Pilon[6] was used to perform error correction based on the Illumina sequences, generating a genome with a scaffold N50 size of 6.48 Mb. The total length of this assembly version was 349.91 Mb. Subsequently, the Hi-C sequencing data were aligned to the assembled scaffolds by BWA-mem[4] and the scaffolds were clustered onto chromosomes with LACHESIS (http://shendurelab.github.io/LACHESIS/), the final genome was 348.53 Mb and the contig and scaffold N50 were 4.36 Mb and 13.52 Mb, respectively.

Quality evaluation for genome assembly

The draft assembly was evaluated by mapping the high-quality reads from short insert-size PE libraries to the scaffolds using BWA[7] mem. The distribution of the sequencing depth at each position was calculated using SAMtools[8] to assess the completeness of the genome assembly. Approximately 94.40% of the reads could be mapped to the assembly, which covered 98.86% of the assembled sequence (Supplementary Table 4).

To assess the quality of the genome assembly, the transcriptome data from *T. wilfordii* were assembled using Trinity[9], and we generated 78,912 unigenes. These unigenes were then mapped to the scaffolds using BLAT[10]. More than 97.06% of these unigenes could be identified in the assembly, indicating that the assembly has good coverage of the gene regions (Supplementary Table 7).

Additionally, the CEGMA[11] (Core Eukaryotic Genes Mapping Approach) pipeline can be used to assess the completeness of the genome assembly or annotations. Analysis of the genome assembly for core eukaryotic genes revealed homologs for 96.77% of conserved genes in the assembly (Supplementary Table 5).

BUSCO[12] (Benchmarking Universal Single-Copy Orthologs) provides quantitative measures for the assessment of genome assembly, gene set, and transcriptome completeness based on evolutionarily informed expectations of gene content from near-universal single-copy orthologs selected from OrthoDB v9. To assess the completeness of the genome assembly, we used BUSCO to find 95.10% conserved genes in the *T. wilfordii* genome (Supplementary Table 6).

Supplementary Note 2

Annotation of repetitive sequences

Transposable elements (TEs) in the *T. wilfordii* genome were searched by combining *de novo*-based and homology-based approaches. For the *de novo* approach, we used RepeatModeler (http://www.repeatmasker.org/RepeatModeler/), LTR_FINDER (http://tlife.fudan.edu.cn/ltr_finder/) and RepeatScout (http://www.repeatmasker.org/) to build a *de novo* repeat library. For the homology-based approach, we used RepeatMasker (version 3.3.0) (http://www.repeatmasker.org/) against the Repbase TE library and RepeatProteinMask (http://www.repeatmasker.org/) against the TE protein database. Tandem repeats were detected in the genome using the software Tandem Repeats Finder (TRF)[13]. Finally, the above results from repetitive sequence annotation were combined, and the repetitive sequence content accounted for 52.36% of the *T. wilfordii* genome (Supplementary Table 10). The most abundant repetitive element repeat type is LTR, which accounts for 34.26% of the genome, including 32.12%

Gypsy LTRs, 32.60% Copia LTRs and 35.28% other types of LTRs (Supplementary Fig. 2).

Annotation of protein-coding genes

To predict protein-coding genes in the *T. wilfordii* genome, we used homology-based prediction, *de novo* prediction and transcriptome-based prediction. Homolog proteins from four plant genomes (*Arabidopsis thaliana*, *Populus trichocarpa, Ricinus communis,* and *Vitis vinifera*) were downloaded from Ensemble Plants (http://plants.ensembl.org/index.html). Protein sequences from these genomes were aligned to the *T. wilfordii* genome assembly using TblastN[14] with an E-value cutoff of $1e^{-5}$. The BLAST hits were conjoined by Solar software[15]. GeneWise[16] was used to predict the exact gene structure of the corresponding genomic regions for each BLAST hit (Homo-set). For transcriptome-based prediction methods, RNA-seq data were mapped to the assembly using Tophat (version 2.0.8)[17] and Cufflinks (version 2.1.1)[18], and then the transcripts were assembled into gene models (Cufflinks-set). In addition, RNA-seq data were assembled by Trinity[9], creating several pseudo-ESTs. These pseudo-ESTs were also mapped to the assembly, and gene models were predicted by PASA[19]. This gene set was denoted PASA-T-set (PASA Trinity set) and was used to train ab initio gene prediction programs. Five ab initio gene prediction programs, Augustus (version 2.5.5)[20], Genscan (version 1.0)[21], GlimmerHMM (version 3.0.1)[22], Geneid[23], and SNAP[24], were used to predict coding regions in the repeat-masked genome. Gene model evidence from the Homo-set, Cufflinks-set, PASA-T-set and ab initio programs was combined by EvidenceModeler (EVM)[25] into a non-redundant set of gene structures. Finally, a total of 28,321 genes were predicted from the *T. wilfordii* genome (Supplementary Tables 8-9).

Functional annotation protein-coding genes

The functional annotation of the protein-coding genes was achieved using BLASTP[26] (with an E-value cutoff of $1e^{-5}$) against two integrated protein sequence databases: SwissProt (https://web.expasy.org/docs/swiss-prot_guideline.html) and NR. Protein domains were annotated by searching against the InterPro (V32.0)[27] and Pfam (V27.0)[28] databases using InterProScan (V4.8)[29] and HMMER (V3.1)[30], respectively. The Gene Ontology (GO) terms for each gene were obtained from the corresponding InterPro or Pfam entry. The pathways in which the genes might be involved were assigned by BLAST against the KEGG databases (release 53) with an E-value cutoff of $1e^{-5}$. A total of 28,201 genes were predicted to be functional, accounting for 99.6% of all genes in the *T. wilfordii* genome (Supplementary Table 11). Annotation features such as the distributions of mRNA length, exon length, exon number, intron length and CDS length are shown in Supplementary Fig. 3.

Non-coding RNA annotation

The tRNA genes were identified by tRNAscan-SE software[31]. The rRNA fragments were predicted by aligning to the rRNA sequences using BlastN with an E-value cutoff of $1e^{-10}$. The miRNA and snRNA genes were predicted by INFERNAL software[32] against the Rfam database (release 9.1)[33]. Finally, we predicted 407 transfer RNA genes, 373 miRNA genes, 892 small nuclear RNA genes, and 2,563 ribosomal RNA genes in the *T. wilfordii* genome (Supplementary Table 12).

Supplementary Note 3

Gene family construction

Protein sequences from *T. wilfordii* and 14 other sequenced plant genomes with

representatives from *Arabidopsis thaliana, Cephalotus follicularis, Carica papaya, Daucus carota, Dimocarpus longan, Glycyrrhiza uralensis, Manihot esculenta, Oryza sativa, Prunus persica, Populus trichocarpa, Solanum lycopersicum, Salvia miltiorrhiza, Theobroma cacao* and *Vitis vinifera* were used for gene family clustering. Then, the gene set of each species was filtered as follows. First, when multiple transcripts were present in one gene, only the longest transcript in the coding region was taken for further analysis; second, the genes encoding proteins less than 30 amino acids long were filtered out. Then, we obtained the similarity relation between all species protein sequences through blastp with an E-value cutoff of $1e^{-5}$. All 15 species protein datasets were clustered into paralogous and orthologous using the program OrthoMCL[34] (http://orthomcl.org/orthomcl/) with the inflation parameter 1.5. All protein-coding genes from 15 sequenced genomes were clustered into 29,872 gene families (two or more members), including 514 single-copy orthologs (Supplementary Fig. 4). Among the *T. wilfordii, Populus trichocarpa, Manihot esculenta,* and *Cephalotus follicularis* gene families, 951 were unique to *T. wilfordii* (Supplementary Fig. 5). These Twi-specific gene families were enriched in the GO terms terpene synthase activity, oxidation-reduction process, and response to stress (Supplementary Table 13) and in KEGG terms including plant-pathogen interaction and monoterpenoid biosynthesis (Supplementary Table 14).

Phylogenetic tree and divergence estimation

After gene family clustering, we aligned all 514 single-copy gene protein sequences by MUSCLE[35] (http://www.drive5.com/muscle/) and combined all the alignment results to creat a super alignment matrix. Then, the 15 species phylogenetic tree was constructed using RAxML[36] (http://sco.h-its.org/exelixis/web/software/raxml/index.html) with the maximum likelihood method and a bootstrap of 100. *Oryza sativa* was designated as an outgroup of the phylogenetic tree. Finally, the MCMCtree program

(http://abacus.gene.ucl.ac.uk/software/paml.html) implemented in phylogenetic analysis by maximum likelihood (PAML)[37] was applied to infer the divergence time based on the phylogenetic tree constructed. The mcmctree running parameters were as follows: burn-in:10,000, sample-number:100,000, and sample-frequency:2. The calibration times of the divergence between *Oryza sativa* and *Arabidopsis thaliana* (148-173 Mya), *Arabidopsis thaliana* and *Solanum lycopersicum* (110-124 Mya), *Arabidopsis thaliana* and *Vitis vinifera* (105-115 Mya), *Carica papaya* and *Populus trichocarpa* (67.5-112.5 Mya), *Arabidopsis thaliana* and *Carica papaya* (54-90 Mya), *Manihot esculenta* and *Populus trichocarpa* (65-86 Mya), *Solanum lycopersicum* and *Daucus carota* (93-107 Mya) were obtained from the TimeTree database[38] (http://www.time.org/). The divergence time between *T. wilfordii* and the ancestor of *Manihot esculenta* and *Populus trichocarpa* was estimated to be approximately 87.1 MYA **(Fig. 1b)**.

Expansion and contraction of gene families

We determined the expansion and contraction of the gene families by comparing the cluster size differences between the ancestor and each species using the CAFÉ program[39]. A random birth and death model was used to study changes of gene families along each lineage of phylogenetic tree. A probabilistic graphical model (PGM) was introduced to calculate the probability of transitions in gene family size from parent to child nodes in the phylogeny. Using conditional likelihoods as the test statistics, we calculated the corresponding p-values in each lineage, and a p-value of 0.05 was used to identify families that were significantly expanded and contracted. Finally, in *T. wilfordii*, 6 gene families were substantially expanded, and 56 gene families were contracted. Functional categories that were enriched for significant gene family expansions mainly included sequence-specific DNA binding, protein binding, protein dimerization activity and so on (Supplementary Table 15).

Positive selected genes

To identify the positively selected genes in *T. wilfordii,* comparing 3 relative species (*Populus trichocarpa, Manihot esculenta,* and *Cephalotus follicularis*), the single-copy genes were aligned using MUSCLE[35]. We used likelihood ratio tests (LRTs) based on branch-site models of PAML[37] to detect positive selection, with *T. wilfordii* as the foreground branch. P-values were computed using the $\chi 2$ statistic and corrected for multiple testing by the false discovery rate (FDR) method. A total of 324 candidate genes in *T. wilfordii* underwent positive selection ($P < 0.05$). Most of them were enriched in the GO terms of nucleoside-triphosphatase activity, and ATPase activity etc., (Supplementary Table 16) and in KEGG terms, including RNA polymerase, nucleotide excision repair and so on (Supplementary Table 17).

Whole genome triplication

To study the evolution of the *T. wilfordii* genome, we searched for whole genome duplication (WGD) in our assembled *T. wilfordii* genome. The protein sequences from *T. wilfordii* and *Vitis vinifera* were searched against themselves using blastp ($E<1e^{-5}$) to identity syntenic blocks. In addition, the protein sequences from *T. wilfordii* were searched against *Vitis vinifera, Populus trichocarpa* and *Manihot esculenta,* respectively. The results were subjected to McscanX[40] to determine syntenic blocks. At least five genes were required to identify synteny. We then calculated the 4DTv (fourfold degenerate synonymous sites of the third codons) for syntenic segments from the concatenated alignments constructed by fourfold degenerate sites of all gene pairs found in each segment and plotted the distribution of the 4DTv values. Two peaks at approximately 0.09 and 0.48 were observed in the *T. wilfordii* genomes, and the first peak at approximately 0.48 revealed the core eudicot gamma triplication event. The second peak at approximately 0.09 indicated that *T. wilfordii* underwent another WGD event after diverging from *Populus trichocarpa* and *Manihot esculenta* (Fig. 1c).

To estimate the timing of the WGD event in *T. wilfordii*, the synonymous substitution rate (*Ks*) values of *T. wilfordii* syntenic block genes were calculated with the codeml program of the PAML package[37], and the distribution of the *Ks* values was plotted (Supplementary Fig. 7). The result of the *Ks* distribution clearly showed a single peak at approximately 0.24. Then, we calculated the mean *Ks* value and standard deviations of WGD duplications. The calculated *Ks* value was converted to the divergence time according to T=*Ks*/2r, where r represented a substitution rate of 6.5×10^{-9} mutations per site per year for eudicots. We dated the *T. wilfordii* WGD (*Ks* = 0.27 ± 0.077) to approximately 20.7 ± 5.94 MYA.

To confirm that *T. wilfordii* did undergo a genome-wide duplication event, we performed synteny analysis on *T. wilfordii* and *Vitis vinifera*. Since its genome underwent minimal rearrangement following the γ event, *V. vinifera* is often used as a reference genome for studying the evolutionary history of eudicot genomes[41]. We detected syntenic blocks across *T. wilfordii* genome and *V. vinifera* genome. Syntenic analysis using the *T. wilfordii* and *V. vinifera* genomes suggested that *T. wilfordii* experienced the γ event and another whole genome triplication event, as suggested by a 1:3 syntenic relationship between *V. vinifera* and *T. wilfordii* (Fig. 1d,e). Taken together, our analysis provides convincing evidence for a single whole genome triplication event in the *T. wilfordii* genome.

Supplementary Note 4

Sample collections and RNA isolation of tissue transcriptome

A total of seven tissues of *T. wilfordii*, including leaf, flower, stem bark, peeled stem, root bark, root phloem and root xylem, were harvested from Taining County, Fujian Province, China. All collected samples were transported by dry ice, washed with

ultrapure water three times, immediately frozen in liquid nitrogen and stored at -80 °C prior to RNA extraction. Total RNA for each tissue was extracted using a modified cetyltrimethylammonium bromide (CTAB) method[42]. Three biological replicate samples from each tissue were analysed.

Sample collections and RNA isolation of suspension cell transcriptome

The *T. wilfordii* suspension cells were cultured in Murashige & Skoog basal medium containing 0.5 mg L^{-1} 2,4-dichlorophenoxyacetic acid (2,4-D), 0.1 mg L^{-1} kinetin (KT), 0.5 mg L^{-1} indole-3-butytric acid (IBA), and 30 g L^{-1} sucrose (pH = 5.8) by incubation in the dark at 25 °C on a rotary shaker[43]. MeJA elicitation started on 13 days after inoculating 2 g fresh weight of suspension cells in 40 mL of medium at a final concentration of 50 μM in a 100-mL shake flask at a final concentration of 50 μM, while control cultures were only treated with the same volume of carrier solution (dimethyl sulfoxide). Cell samples were harvested by filtration 0, 4, 8, 12, 24, 36, 48, 72, 96, 120, 240, 360 and 480 h after elicitation and the addition of DMSO. Three cell samples were stored at -20 °C for each time point in each group for metabolite and transcript profiling. The total RNA for each suspension cell was extracted using the Eastep® Super Total RNA Extraction Kit (Promega). Three biological replicate samples from suspension cells were analysed.

RNA sequencing and assembly

The total RNA was extracted from all samples using genomic DNA contamination and removed using RNase-Free DNase I (TIANGEN). The integrity of RNA was evaluated on a 1.0% agarose gel stained with ethidium bromide (EB), and its quality and quantity were assessed using an Agilent 2100 Bioanalyzer (Agilent Technologies). Then, the integrate RNA was used in cDNA library construction and Illumina sequencing. The cDNA library was constructed using the NEBNext Ultra RNA

Library Prep Kit for Illumina (NEB), following the manufacturer's recommendations. Library preparations were sequenced on an Illumina HiseqXTen platform, generating 150-bp paired-end reads.

Transcriptome analysis

Raw RNA reads were filtered and trimmed to yield clean reads and these high-quality reads were mapped to the draft reference genomes by TopHat2[17] with following the parameters: --max-intron-length 500000, --read-gap-length 10, --read-edit-dist 15, --max-insertion-length 5 and --max-deletion-length 5. The expression level (RPKM value) for each protein-coding gene was calculated by HTSeq[44] using default parameters. DESeq2[45] were used for nomorlizing gene expression (BaseMean) in each sample, and identified differentially expressed genes (DEGs) for each compared group by using P-adj (adjusted p value) < 0.05 as the threshold. Go enrichment analysis of DEGs was implemented by the GOseq R package[46], in which the gene length bias was corrected. GO terms with corrected P-values less than 0.05 were considered significantly enriched by DEGs. KEGG is a database resource for understanding high-level functions and utilities of the biological system, such as the cell, the organism and the ecosystem, from molecular-level information, especially large-scale molecular datasets generated by genome sequencing and other high-throughput experimental technologies (http://www.genome.jp/kegg/). We used KOBAS software[47] to test the statistical enrichment of DEGs in KEGG pathways. Pathways with q-values < 0.05 were considered significantly enriched.

Supplementary Note 5

Ultra-performance liquid chromatography (UPLC) analysis

The UPLC separation was performed using an Agilent Technologies 1290 Infinity

II system (Agilent Technologies, Santa Clara, CA, USA) with a Waters ACQUITY UPLC HSS T3 analytical column (2.1 mm × 100 mm, 1.8 μm) kept at 40 °C. The mobile phase, consisting of a mixture of 0.1% (v/v) acetic acid in water (A) and acetonitrile (B), was pumped at a flow rate of 0.4 mL min^{-1}. The gradient elution was programmed as follows: 0 min at 30% B, 5 min at 35% B, 8 min at 35% B, 15 min at 70% B, and 21 min at 90% B.

Metabolite and transcript profiling of the triptolide biosynthetic pathway

Terpenoids could be induced by MeJA, and some genes involved in terpenoid biosynthesis were affected by MeJA in previous reports[48, 49]. To gain a deeper understanding of triptolide-related genes and metabolites, we performed a suspension experiment induced by MeJA. The results of the induction experiment showed that the application of MeJA enhanced the production of triptolide and triptophenolide (Fig. 2). The triptolide level in MeJA-induced cells reached 145.6 μg g^{-1}, which was 3.60-fold higher than that in the control group at 360 h, while the triptophenolide level was 55.11-fold higher with 730.0 μg g^{-1} in the induced suspension cells. We then performed ultra-performance liquid chromatography-quadrupole time of flight-mass spectrometry (UPLC/Q-TOF MS) analysis and PCA analysis to define the dimensions of the large data sets and identify significant signals[50]. A clear separation of the cellular components between the control and MeJA-induced groups was found after 36 h (Supplementary Fig. 11), and peak filtering resulted in a final set of 142 peaks with a mass ratio of 295 to 400, of which 98 were upregulated and 44 were downregulated by MeJA (Supplementary Figs. 12-14). The unassigned peaks containing metabolites displaying masses in the range of 295-400 m/z were within the most expected range for diterpenoids in *T. wilfordii* and thus might constitute yet-unknown intermediates or side products involved in the triptolide biosynthetic pathway. Furthermore, we calculated the expression levels of these pathway-related genes at different times in the two groups, and most of the genes were significantly

upregulated after induction (Fig. 2). Due to the very significant increase in the content of both triptolide and triptophenolide, the downstream pathway of CYP450 genes were likely to be in an upregulated state.

We examined the metabolites and tissue-specific expression patterns of genes in seven different tissues, including leaf, flower, stem bark, peeled stem, root bark, root phloem and root xylem. The three parts of the root were significantly different in composition from the leaf, flower, stem bark and peeled stem (Supplementary Fig. 15). Among the 98 metabolite peaks upregulated mentioned above, 35 were detected in the tissues, and the vast majority of them had the highest content in the root bark, including triptolide and triptophenolide (Supplementary Fig. 16). These results revealed that the main synthetic site of triptolide may be in the root bark. Based on the differential expression analysis of root bark and six other tissues, gene functions showing significant differential expression in the root bark were mainly enriched in the GO terms oxidation-reduction process, terpene synthase activity and oxidoreductase activity etc., and in KEGG terms including diterpenoid biosynthesis, biosynthesis of secondary metabolites, plant hormone signal transduction and so on (Supplementary Fig. 17). At the same time, the expression levels of triptolide pathway-related genes in different tissues showed that most of the genes were highly expressed in the root bark (Supplementary Fig. 18).

Supplementary Note 6

Construction of miltiradiene producing yeast strain

A previous study confirmed that miltiradiene acted as the olefin precursor of triptolide[49]; therefore, a miltiradiene producing yeast strain will promote the identification of the function of subsequent CYP450 genes. We introduced a single *SmMS-SmCPS1* fusion module into the yeast chromosome of BY-HZ16 (PS1 strain),

yielding 1.17 mg L^{-1} miltiradiene, whereas the PS2 strain had double *SmMS-SmCPS1* modules, leding to a 7.03-fold increase to 8.23 mg L^{-1} miltiradiene (Supplementary Fig. 23).

NMR analysis

Dehydroabietic acid (**1**) (3.0 mg, white amorphous powder), HR-ESI-MS (neg) *m/z* 299.2014 $[M-H]^-$ (Calcd. for $C_{20}H_{28}O_2$, 300.2089). ^1H NMR ($CDCl_3$, 800 MHz): δ = 2.31-2.32 (1H, m, H-1a), 1.48-1.51 (1H, m, H-1b), 1.76-1.80 (2H, m, H-2), 1.81-1.82 (1H, m, H-3a), 1.70-1.74 (1H, m, H-3b), 2.25 (1H, dd, *J* = 12.0, 1.6 Hz, H-5), 1.83-1.88 (1H, m, H-6a), 1.52-1.55 (1H, m, H-6b), 2.92-2.95 (1H, m, H-7a), 2.86-2.89 (1H, m, H-7b), 7.17 (1H, d, *J* = 8.0 Hz, H-11), 7.00 (1H, d, *J* = 8.0 Hz, H-12), 6.88 (1H, brs, H-14), 2.80-2.84 (1H, m, H-15), 1.20 (3H, d, *J* = 4.8 Hz, H-16), 1.20 (3H, d, *J* = 4.8 Hz, H-17), 1.28 (1H, s, H-19), 1.22 (1H, s, H-20); ^{13}C NMR ($CDCl_3$, 200 MHz): δ = 37.9 (C-1), 18.5 (C-2), 36.7 (C-3), 47.4 (C-4), 44.6 (C-5), 21.8 (C-6), 30.0 (C-7), 134.7 (C-8), 146.7 (C-9), 36.9 (C-10), 124.1 (C-11), 123.9 (C-12), 145.7 (C-13), 126.9 (C-14), 33.5 (C-15), 24.0 (C-16), 24.0 (C-17), 183.9 (C-18), 16.3 (C-19), 25.1 (C-20) [51, 52, 53, 54].

Miltiradienoic acid (**2**) (2.9 mg, white amorphous powder), HR-ESI-MS (neg) *m/z* 301.2170 $[M-H]^-$ (Calcd. for $C_{20}H_{30}O_2$, 302.2246). ^1H NMR ($CDCl_3$, 800 MHz): δ =1.52-1.54 (1H, m, H-1a), 1.59-1.61 (1H, m, H-1b), 1.56-1.58 (1H, m, H-2a), 1.63-1.64 (1H, m, H-2b), 1.78-1.80 (1H, m, H-3b), 2.08-2.11 (1H, m, H-3b), 1.66-1.70 (1H, m, H-5), 1.75-1.76 (1H, m, H-6a), 1.64-1.66 (1H, m, H-6b), 1.89-1.92 (1H, m, H-7a), 2.06-2.08 (1H, m, H-7b), 2.36-2.40 (1H, m, H-11a), 2.63-2.65 (2H, m, H-11b, 14b), 5.45 (1H, dt, *J* = 1.6, 4.8 Hz, H-12), 2.48-2.53 (1H, m, H-14a), 2.16-2.19 (1H, H-15), 1.02 (3H, d, *J* = 4.8 Hz, H-16), 1.01 (3H, d, *J* = 4.8 Hz, H-17), 1.22 (3H, s, H-19), 1.00 (3H, s, H-20); ^{13}C NMR ($CDCl_3$, 200 MHz): δ = 36.5 (C-1), 18.2 (C-2), 34.2 (C-3), 47.5 (C-4), 45.8 (C-5), 19.0 (C-6), 25.2 (C-7), 124.2 (C-8), 134.6 (C-9),

36.7 (C-10), 31.3 (C-11), 116.2 (C-12), 140.0 (C-13), 35.9 (C-14), 33.0 (C-15), 21.3 (C-16), 21.1 (C-17), 183.9 (C-18), 21.5 (C-19), 19.7 (C-20).

Dehydroabietinol (**3**) (3.1 mg, colorless acicular crystal), HR-ESI-MS (pos) *m/z* 287.2364 $[M+H]^+$ (Calcd. for $C_{20}H_{30}O$, 286.2297). ^1H NMR ($CDCl_3$, 800 MHz): δ = 1.78-1.80 (1H, m, H-1a), 1.43-1.45 (1H, m, H-1b), 1.64-1.65 (2H, m, H-2), 1.37-1.40 (2H, m, H-3), 2.28 (1H, dd, J = 12.0, 1.6 Hz, H-5), 1.79-1.81 (1H, m, H-6a), 1.71-1.74 (1H, m, H-6b), 2.89-2.92 (1H, m, H-7a), 2.84-2.87 (1H, m, H-7b), 7.18 (1H, d, J = 8.0 Hz, H-11), 6.99 (1H, dd, J = 8.0, 2.4 Hz, H-12), 6.88 (1H, brs, H-14), 2.80-2.83 (1H, m, H-15), 1.22 (3H, d, J = 4.8 Hz, H-16), 1.22 (3H, d, J = 4.8 Hz, H-17), 3.48 (1H, d, J = 10.4 Hz, H-18a), 3.24 (1H, d, J = 10.4 Hz, H-18b), 1.22 (1H, s, H-19), 0.89 (1H, s, H-20); ^{13}C NMR ($CDCl_3$, 200 MHz): δ = 38.4 (C-1), 18.8 (C-2), 35.1 (C-3), 37.8 (C-4), 43.9 (C-5), 18.6 (C-6), 30.1 (C-7), 134.7 (C-8), 147.3 (C-9), 37.3 (C-10), 124.2 (C-11), 123.8 (C-12), 145.5 (C-13), 126.8 (C-14), 33.4 (C-15), 24.0 (C-16), 24.0 (C-17), 72.3 (C-18), 17.4 (C-19), 25.3 (C-20)[52, 53, 55, 56].

Miltiradienol (**4**) (2.4 mg, colorless acicular crystal), HR-ESI-MS (pos) *m/z* 289.2531 $[M+H]^+$ (Calcd. for $C_{20}H_{32}O$, 288.2453). **^1H** NMR ($CDCl_3$, 800 MHz): δ =1.52-1.54 (1H, m, H-1a), 1.61-1.63 (1H, m, H-1b), 1.56-1.58 (1H, m, H-2a), 1.63-1.65 (1H, m, H-2b), 1.38 (2H, td, J = 13.6, 4.0 Hz, H-3), 1.51-1.52 (1H, m, H-5), 1.73-1.75 (1H, m, H-6a), 1.64-1.66 (1H, m, H-6b), 1.91-1.94 (1H, m, H-7a), 2.01-2.05 (1H, m, H-7b), 2.37-2.41 (1H, m, H-11a), 2.61-2.65 (2H, m, H-11b, 14b), 5.45 (1H, dt, J = 1.6, 4.8 Hz, H-12), 2.48-2.52 (1H, m, H-14a), 2.16-2.19 (1H, H-15), 1.02 (3H, d, J = 4.8 Hz, H-16), 1.01 (3H, d, J = 4.8 Hz, H-17), 3.19 (1H, d, J = 11.2 Hz, H-18b), 3.44 (1H, d, J = 11.2 Hz, H-18b), 0.82 (3H, s, H-19), 1.03 (3H, s, H-20); ^{13}C NMR ($CDCl_3$, 200 MHz): δ = 36.4 (C-1), 18.6 (C-2), 34.2 (C-3), 37.0 (C-4), 45.1 (C-5), 18.4 (C-6), 25.5 (C-7), 123.9 (C-8), 135.3 (C-9), 37.7 (C-10), 31.5 (C-11), 116.3 (C-12), 139.9 (C-13), 35.1 (C-14), 33.1 (C-15), 21.3 (C-16), 21.1 (C-17), 72.7 (C-18), 17.4 (C-19), 20.0 (C-20).

Supplementary Note 7

Functional identification of the other 9 candidate CYP450s

To explore the potential functions of the other 9 candidate CYP450s, *in vitro* enzymatic activity assays were performed using accessible intermediates including dehydroabietic acid, triptinin B, triptophenolide and triptoquinonide, which were most likely involved in the triptolide biosynthetic pathway. However, no new compounds were produced, indicating that these specific CYP450s could not catalyze the transformation of these intermediates, and more intermediates should be tested (Supplementary Fig. 33).

Supplementary Note 8

Multi-level regulation of triptolide

Transcription factors (TFs) are critical to the regulation of gene expression levels. TFs were identified using the ITAK program (http://itak.feilab.net/cgi-bin/itak/index.cgi). Notably, 2833 TFs were identified and classified into 89 families from *T. wilfordii*, which is substantially more than in most of sequenced plant genomes (Supplementary Data 8). We explored TFs that regulate the triptolide biosynthetic pathway through correlation analysis after gene expression changes induced by MeJA (Supplementary Data 9). The gene expression patterns identified TFs and the triptolide biosynthetic genes were used to construct the co-expression network using the PCC method in the R platform between each set of variables to calculate the Pearson correlation coefficient, and significant positive correlations with p-value < 0.05 were detected between TFs and genes. The correlation network was analysed when the correlation coefficient was >0.9 by Cytoscape software (version 3.6.1)[57].Among the 89 TF families, the expression patterns of triptolide-related genes were most strongly associated with the bHLH, HB, ERF, WRKY, C2H2 and bZIP families, consistent

with previous reports on transcriptional regulation of terpenoid biosynthesis[58]. However, the regulation of triptolide biosynthesis in *T. wilfordii* may be a relative complex system, with several unusual TFs also showing strong associations, such as members of the NAC, MYB, Tify, C3H and GRAS families (Supplementary Fig. 34 and Supplementary Data 10). These TFs are associated with abiotic and/or biotic stress responses[59, 60], which is consistent with the reported antibacterial and anti-insect activities of triptolide[61]. In addition, TFs can provide either positive or negative regulation, and some genes potentially involved in triptolide biosynthesis are negatively correlated with TFs, such as *IDI* (TW020293.1). Other potentially relevant genes are particularly tightly linked to positive regulation by TFs, especially two isoforms of *DXS* (TW000900.1, TW008579.1). Perhaps not surprisingly, the correlation between the different members of the same gene family varied, indicating that regulation of the triptolide pathway can be intricately controlled by differential regulation of expanded gene families.

Knowledge of TFs involved in the biosynthesis of terpenoids is still fragmentary. Since most of the known pathway genes of triptolide were up-regulated within 36 hours after MeJA induction, TFs should change transcription levels at an earlier time after induction. Thus, we analysed differential expression of TFs after MeJA induction (Supplementary Data 11-13). Most of TFs showed changes in transcription levels at 4 hours and 12 hours, of which 235 were up-regulated at 4 h and 162 were up-regulated at 12 h. These TFs mainly belong to the WRKY, bHLH, bZIP, NAC, ERF, and other families, which are consistent with the results of correlation analysis above. Therefore, these TFs are most likely involved in the regulation of the biosynthesis of triptolide. Given that nothing is yet known about TFs associated with triptolide biosynthesis, our work affords insight by revealing potential candidates for future investigation.

Supplementary Table 1. Survey statistic results of *T. wilfordii.*

Species	Total base (Gb)	K-mer	K-mer number	K-mer depth	Genome size (Mb)	Revised genome size (Mb)	Heterozygous ratio (%)	Repeat ratio (%)
Twi	39.83	17	29,152,494,988	78	373.75	365.95	1.95	48.87

Supplementary Table 2. Sequencing data statistics of *T. wilfordii.*

Pair-end libraries	Insert size	Total data (Gb)	Reads length (bp)	Sequence coverage (X)
Illumina reads	350 bp	39.83	150	108.84
Pacbio reads	60 kb	75.79	-	207.10
10X Genomics	-	119.75	150	327.23
Hi-C	-	54.77	150	149.67
Total	-	225.37	-	793.34

Supplementary Table 3. Summary of the final genome assembly of *T. wilfordii.*

Version	V0.1	V1.0
Data source	PacBio+10X	V0.1+Hi-C
Total scaffolds	349,906,186	348,533,228
Number of scaffolds	232	321
Scaffold N50 (bp)	6,482,545	13,518,038
Scaffold L50	15	12
Scaffold N90 (bp)	1,275,922	10,826,392
Scaffold L90	54	23
Total contigs	348,378,095	348,378,095
Number of contigs	467	467
Contig N50 (bp)	4,362,171	4,362,171
Contig L50	30	30
Contig N90 (bp)	265,048	265,048
Contig L90	137	137

Supplementary Table 4. Coverage statistics of *T. wilfordii* genome.

		**Percentage**
Reads	Mapping rate (%)	94.40%
Genome	Coverage (%)	98.86%
	Coverage at least 4X (%)	98.17%
	Coverage at least 10X (%)	97.63%
	Coverage at least 20X (%)	96.98%
	Average sequencing depth	87.48X

Average sequence depth: The average depth of each base on the genome that is covered by reads; Coverage**:** The proportion of genomes that were covered by reads.

Supplementary Table 5. Assessment the gene coverage rate using CEGMA.

**species**	**complete**		**complete + partial**	
	**# Prots**	**%completeness**	**# Prots**	**%completeness**
Twi	234	94.35	240	96.77

Supplementary Table 6. Assessment the gene coverage rate using BUSCO.

**Species**	**Size (Mbp)**	**BUSCO notation assessment results**
Twi	365.95	C:95.1%, F:1.6%, M:3.3%, n:1440

Size: genome size; BUSCO notation: C: Complete Single-Copy BUSCOs; D: Complete Duplicated BUSCOs; F: Fragmented BUSCOs; M: Missing BUSCOs; n: Total BUSCO groups searched.

Supplementary Table 7. The EST evaluation results of *T. wilfordii* genome.

**Dataset**	**Number**	**Total length (bp)**	**with >90% sequence in one scaffold**		**with >50% sequence in one scaffold**	
			**Number**	**Percent (%)**	**Number**	**Percent (%)**
>0bp	78,912	39,462,823	71,928	91.15	76,595	97.064
>200bp	78,912	39,462,823	71,928	91.15	76,595	97.064
>500bp	20,167	21,596,707	18,560	92.032	19,749	97.927
>1k	7,022	12,714,172	6,506	92.652	6,898	98.234
>2k	2,031	5,822,347	1,895	93.304	2,003	98.621

Supplementary Table 8. Basic statistical results of gene structure prediction of *T. wilfordii* genome.

Gene set		Number	CDS + intron length (bp)	Average CDS length (bp)	Average exon length (bp)	Average intron length (bp)	Average exons per gene
De novo	Augustus	26,614	3,191.4	1,240.96	237.65	462	5.22
	GlimmerHMM	44,406	5,128.41	784.34	236.26	1,872.62	3.32
	SNAP	24,640	3,369.79	910.37	197.88	683.08	4.6
	Genscan	24,353	8,025.28	1,456.68	210.98	1,112.51	6.9
	Geneid	35,924	4,236.24	998.21	203.75	830.45	4.9
Homolog	*A. thaliana*	33,590	2,319.33	1,038.29	264.19	437.21	3.93
	P. trichocarpa	30,841	2,586.01	1,166.08	267.91	423.55	4.35
	R. communis	27,820	2,943.75	1,273.54	268.93	447.12	4.74
	V. vinifera	28,694	2,695.8	1,148.18	252.96	437.31	4.54
RNA-seq	Cufflinks	56,820	5,490.69	1,864.12	280.51	642.38	6.65
	PASA	56,057	2,524.83	931.32	217.18	484.62	4.29
EVM		30,264	3,297.96	1,188.5	225.83	494.87	5.26
PASA-update		30,083	3,232.43	1,203.23	228.83	476.54	5.26
Final set		28,321	3,337.54	1,240.60	228.24	472.76	5.44

Supplementary Table 9. Basic statistical results of gene structure prediction of *T. wilfordii* and relative species.

Species	Number	CDS + intron length (bp)	Average CDS length (bp)	Average exon length (bp)	Average intron length (bp)	Average exons per gene
T. wilfordii	28,321	3,338	1,241	228	473	5.44
A. thaliana	27,416	1,870	1,218	238	158	5.13
P. trichocarpa	41,377	2,318	1,116	238	326	4.68
R. communis	20,452	3,281	1,331	239	426	5.58
V. vinifera	29,927	4,729	1,096	231	969	4.75

Supplementary Table 10. Summary of Repeat contents in *T. wilfordii* genome.

Type	Length (bp)	Percent (%)
Tandem repeats	79,601,469	22.84
TE repeats	179,579,265	51.52
DNA	20,247,720	5.81
LINE	10,877,255	3.12
SINE	532,409	0.15
LTR	119,428,134	34.26
Unknown	9,809,878	2.81
Total	182,515,114	52.36

Supplementary Table 11. The statistical results of gene function annotation of *T. wilfordii* genome.

Database		Annotated Number	Annotated Percent (%)
NR		27,437	96.9
Swiss-Prot		22,571	79.7
KEGG		21,148	74.7
InterPro	All	28,055	99.1
	Pfam	22,451	79.3
	GO	25,972	91.7
Annotated		28,201	99.6
Total		28,321	-

Supplementary Table 12. The statistical results of non-coding RNA of *T. wilfordii* genome.

Type		Copy	Average length (bp)	Total length (bp)	% of genome
miRNA		373	123.05	45,898	0.013
tRNA		407	75.02	30,532	0.009
rRNA	rRNA	2,563	371.18	951,328	0.273
	18S	490	1,348.13	660,585	0.190
	28S	1,424	141.26	201,153	0.058
	5.8S	361	157.08	56,706	0.016
	5S	288	114.18	32,884	0.009
snRNA	snRNA	892	107.71	96,073	0.028
	CD-box	698	100.73	70,309	0.020
	HACA-box	79	125.58	9,921	0.003
	splicing	114	137.87	15,717	0.005

Supplementary Table 13. The GO enrichment of *Twi*-specific genes.

GO_ID	GO_Term	GO_Class	AdjustedPv
GO:0009055	electron carrier activity	MF	$2.61E^{-19}$
GO:0005507	copper ion binding	MF	$1.85E^{-16}$
GO:0020037	heme binding	MF	$1.21E^{-13}$
GO:0005506	iron ion binding	MF	$1.64E^{-08}$
GO:0047134	protein-disulfide reductase activity	MF	$1.50E^{-07}$
GO:0016651	oxidoreductase activity, acting on NAD(P)H	MF	$9.02E^{-07}$
GO:0030246	carbohydrate binding	MF	$1.26E^{-06}$
GO:0030247	polysaccharide binding	MF	$2.69E^{-05}$
GO:0055114	oxidation-reduction process	BP	$7.34E^{-05}$
GO:0046914	transition metal ion binding	MF	0.000418517
GO:0010333	terpene synthase activity	MF	0.001073554
GO:0006950	response to stress	BP	0.00117363
GO:0008097	5S rRNA binding	MF	0.001245914
GO:0033897	ribonuclease T2 activity	MF	0.001482161
GO:0005615	extracellular space	CC	0.002114071
GO:0005576	extracellular region	CC	0.003153772
GO:0007275	multicellular organismal development	BP	0.003648027
GO:0044183	protein binding involved in protein folding	MF	0.004885662
GO:0045900	negative regulation of translational elongation	BP	0.004885662
GO:0005673	transcription factor TFIIE complex	CC	0.004885662
GO:0004360	glutamine-fructose-6-phosphate transaminase (isomerizing) activity	MF	0.004885662

* $P < 0.05$ by hypergeometric test and FDR adjustments.

Supplementary Table 14. The KEGG enrichment of *Twi*-specific genes.

Term	Database	AdjustedPv
Photosynthesis - antenna proteins	KEGG PATHWAY	$3.61E^{-12}$
Plant-pathogen interaction	KEGG PATHWAY	$1.51E^{-07}$
Protein processing in endoplasmic reticulum	KEGG PATHWAY	$3.05E^{-05}$
Monoterpenoid biosynthesis	KEGG PATHWAY	0.002026133
Phosphatidylinositol signaling system	KEGG PATHWAY	0.002631333
Glutathione metabolism	KEGG PATHWAY	0.004442238
Protein export	KEGG PATHWAY	0.005634523
Ribosome	KEGG PATHWAY	0.012220486
Ubiquitin mediated proteolysis	KEGG PATHWAY	0.016212495

* $P < 0.05$ by hypergeometric test and FDR adjustments.

Supplementary Table 15. The GO enrichment of *T. wilfordii* expansion genes.

GO_ID	GO_Term	AdjustedPv
GO:0043565	sequence-specific DNA binding	$7.50E^{-19}$
GO:0003677	DNA binding	$1.16E^{-16}$
GO:0003700	sequence-specific DNA binding transcription factor activity	$5.36E^{-16}$
GO:0005488	binding	$4.69E^{-07}$
GO:0005515	protein binding	$1.90E^{-05}$
GO:0046983	protein dimerization activity	0.001018957

* $P < 0.05$ by hypergeometric test and FDR adjustments.

Supplementary Table 16. The GO enrichment of *T. wilfordii* positive selected genes.

GO_ID	GO_Term	GO_Class	Adjusted Pv
GO:0017111	nucleoside-triphosphatase activity	MF	$2.56E^{-08}$
GO:0004386	helicase activity	MF	$1.00E^{-07}$
GO:0003676	nucleic acid binding	MF	$1.51E^{-07}$
GO:0008026	ATP-dependent helicase activity	MF	$5.34E^{-07}$
GO:0042623	ATPase activity, coupled	MF	$4.66E^{-06}$
GO:0016787	hydrolase activity	MF	$8.18E^{-06}$
GO:0016887	ATPase activity	MF	$2.30E^{-05}$
GO:0090304	nucleic acid metabolic process	BP	$4.27E^{-05}$
GO:0006281	DNA repair	BP	0.000806702

* $P < 0.05$ by hypergeometric test and FDR adjustments.

Supplementary Table 17. The KEGG enrichment of *T. wilfordii* positive selected genes.

Term	Database	AdjustedPv
RNA polymerase	KEGG PATHWAY	0.007089115
Purine metabolism	KEGG PATHWAY	0.010614018
Pyrimidine metabolism	KEGG PATHWAY	0.01694719
Nucleotide excision repair	KEGG PATHWAY	0.021243179

* $P < 0.05$ by hypergeometric test and FDR adjustments.

Supplementary Table 18. Copy number variation of genes involved in the triptolide biosynthesis in the 15 plant species.

Symbol	*Twi*	*Ath*	*Cpa*	*Dca*	*Dlo*	*Gma*	*Gra*	*Gur*	*Osa*	*Ppe*	*Ptr*	*Sly*	*Smi*	*Tca*	*Vvi*
ACAT	4	2	3	4	7	8	5	4	4	4	5	5	3	4	4
DXS	8	3	3	3	4	10	5	2	2	3	5	3	5	5	6
CMK	2	1	0	1	1	2	2	1	1	1	2	1	1	1	1
DXR	1	1	1	1	1	3	2	2	1	1	2	1	1	1	1
HDR	2	1	1	3	1	2	3	1	2	1	2	1	1	1	1
HDS	2	1	1	2	1	2	1	1	1	1	2	1	1	1	1
HMGR	3	2	3	2	3	7	9	4	2	3	6	3	4	3	3
HMGS	2	1	1	1	1	5	2	3	3	1	3	3	1	2	2
IDI	3	2	1	1	2	2	1	0	2	1	1	2	0	1	1
MCT	4	3	2	3	3	3	2	1	3	2	3	2	1	2	2
MCS	1	1	1	1	2	2	2	1	1	1	2	1	1	1	0
MVK	2	1	1	1	2	2	2	1	1	1	2	1	1	1	1
MVD	2	2	1	0	1	2	1	0	1	1	2	2	1	1	1
PMK	2	1	1	2	1	2	1	1	1	1	2	2	0	1	1
FPS	2	2	1	1	2	2	2	1	4	2	1	3	1	2	1
GPS	3	1	2	4	2	4	3	3	2	2	3	2	2	4	2
GGPPS	4	11	3	11	10	9	11	7	5	6	10	8	7	9	6
TPS	40	34	21	36	102	37	46	33	39	25	65	51	27	41	104
CYP450	228	259	170	319	386	440	332	260	334	302	417	253	297	300	385

Species names were abbreviated as follows: *Tripterygium wilfordii* (*Twi*), *Arabidopsis thaliana* (*Ath*), *Carica papaya* (*Cpa*), *Daucus carota* (*Dca*), *Dimocarpus longan* (*Dlo*), *Glycine max* (*Gma*), *Gossypium raimondii* (*Gra*), *Glycyrrhiza uralensis* (*Gur*), *Oryza sativa* (*Osa*), *Prunus persica* (*Ppe*), *Populus trichocarpa* (*Ptr*), *Solanum lycopersicum* (*Sly*), *Salvia miltiorrhiza* (*Smi*), *Theobroma cacao* (*Tca*), *Vitis vinifera* (*Vvi*)

Supplementary Table 19. *Ks* values and duplication/divergence times of genes involved in triptolide biosynthesis in *T. wilfordii*.

Symbol	Gene ID	Duplicated Gene ID	*Ka*	*Ks*	Duplication Time (Mya)
ACAT	TW025077.1	TW024523.1	0.0168	0.2067	15.90
FPS	TW001817.1	TW004801.1	0.1269	1.2733	97.95
GGPPS	TW001897.1	TW005800.1	0.6635	2.4501	184.62
GPS	TW007487.1	TW029911.1	0.0351	0.2351	18.08
HMGR	TW003170.1	TW027293.1	0.0228	0.1656	12.74
IDI	TW024713.1	TW029745.1	0.0485	0.2745	21.12
MCT	TW011900.1	TW031000.1	0.0572	0.1667	12.82
MVD	TW001485.1	TW023960.1	0.0188	0.2272	17.48
HMGS	TW000414.1	TW031662.1	0.0265	0.2067	15.90
CMK	TW021748.1	TW013910.1	0.1196	0.2232	17.17
HDR	TW005049.1	TW010180.1	0.0364	0.2036	15.66
HDS	TW014055.1	TW019187.1	0.0289	0.1618	12.45
MVK	TW015762.1	TW019597.1	0.0768	0.2481	19.08
PMK	TW021225.1	TW031844.1	0.0479	0.1947	14.98
CPS	TW032221.1	TW023853.1	0.0869	0.3071	23.62
	TW010739.1	TW023867.1	0.338	0.987	75.92
MS	TW023854.1	TW026987.1	0.1855	0.3028	23.29

Supplementary Table 20. Screening 10 CYP450 genes as candidates.

Gene ID	CYP450 Family	Possible function	Reference
TW017699.1	CYP716C	unknown pathway	-
TW018099.1	CYP81AM	unknown pathway	-
TW011445.1	CYP81AM	unknown pathway	-
TW017431.1	CYP76A	unknown pathway	-
TW031838.1	CYP76Y	unknown pathway	-
TW023804.1	CYP82J	unknown pathway	-
TW012149.1	CYP82AS	unknown pathway	-
TW006625.1	CYP712K	unknown pathway	-
TW009876.1	CYP728B	diterpenoid pathway	This study
TW019296.1	CYP94C	jasmonate biosynthesis and signaling	62

Supplementary Table 21. All strains used in this study.

Strains	Genotype or characteristic	Source
BY-T20	BY4742 *ΔTrp1,Trp1::His3-P_{PGK1}-BTS1/ERG20-T_{ADH1}-P_{TDH3}-SaGGPPS-T_{TPI1}-P_{TEF1}-tHMG1-T_{CYC1}*	[63, 64]
BY-HZ16	BY-T20 *rox1Δ erg9::Δ-220-176 yjl064wΔ ypl062wΔ*	This study
PS1	BY-HZ16, *YPRCΔ15 Ura3-P_{ADH1}-SmMS/SmCPS1-T_{PGI}*	This study
PS2	BY-HZ16, *YPRCΔ15 Ura3-P_{TDH3}-SmMS/SmCPS1-T_{TPI1}-P_{ADH1}-SmMS/SmCPS1-T_{PGI}*	This study
PS3	BY-HZ16, *YPRCΔ15 Ura3-P_{TDH3}-SmMS/SmCPS1-T_{TPI1}-P_{ADH1}-SmMS/SmCPS1-T_{PGI}-P_{PGK1}-TwCYP728B70-T_{ADH1}/P_{TEF2}-TwCPR3-T_{CYC1}*	This study

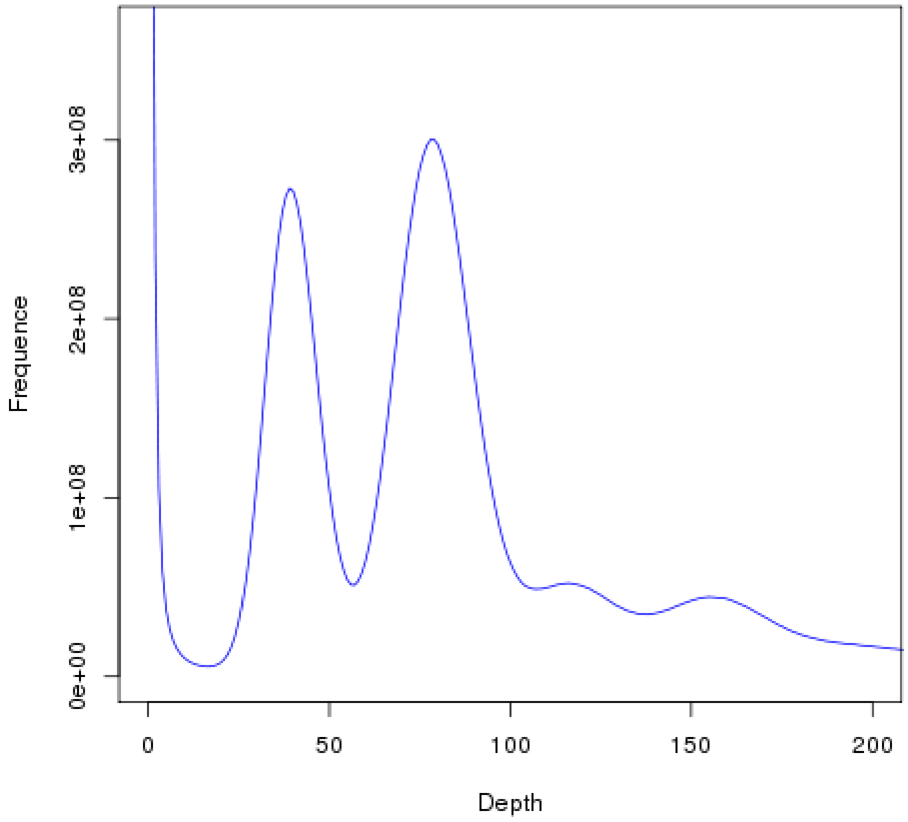

Supplementary Figure 1. K-mer analysis for estimating the genome size of *T. wilfordii*.

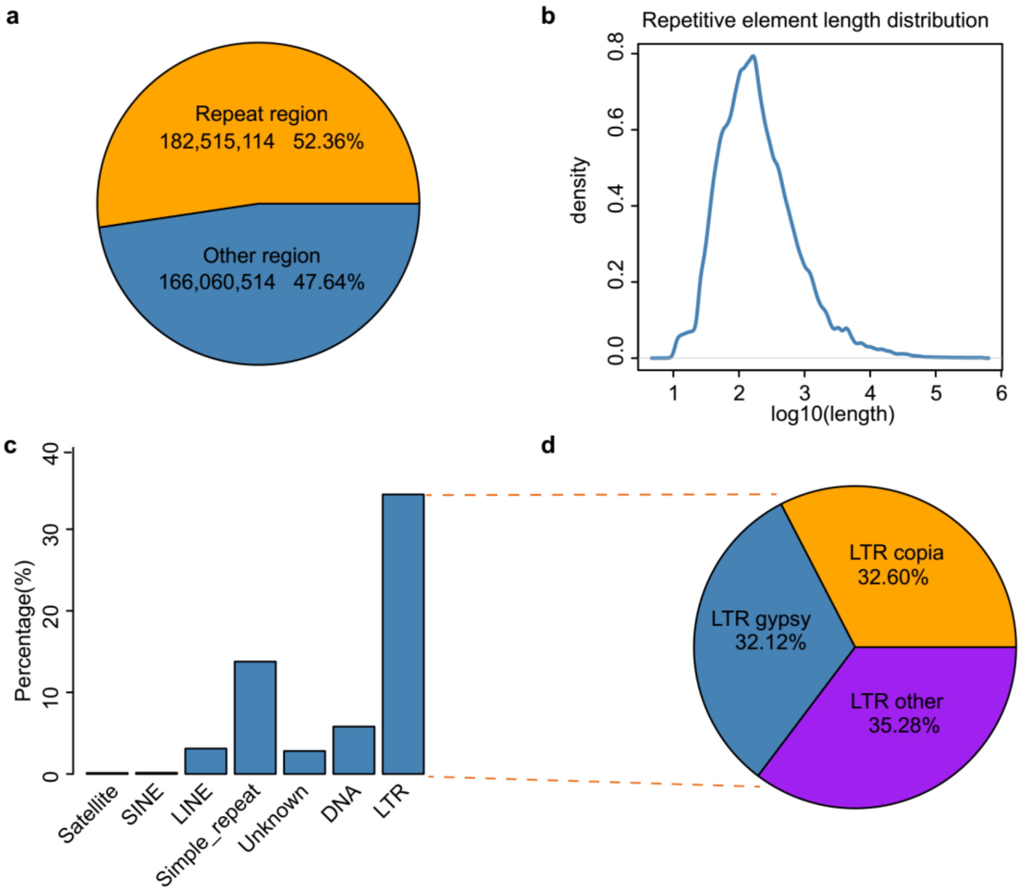

Supplementary Figure 2. Characteristics of repetitive elements in the *T. wilfordii* genome. a, The proportions of repetitive elements in the *T. wilfordii* genome. **b**, The length distribution of repetitive elements. **c**, The proportions of different classes of repetitive elements in the *T. wilfordii* genome. The LTRs are the most abundant repetitive elements (n=1 biologically independent samples). **d**, The proportions of different LTR types.

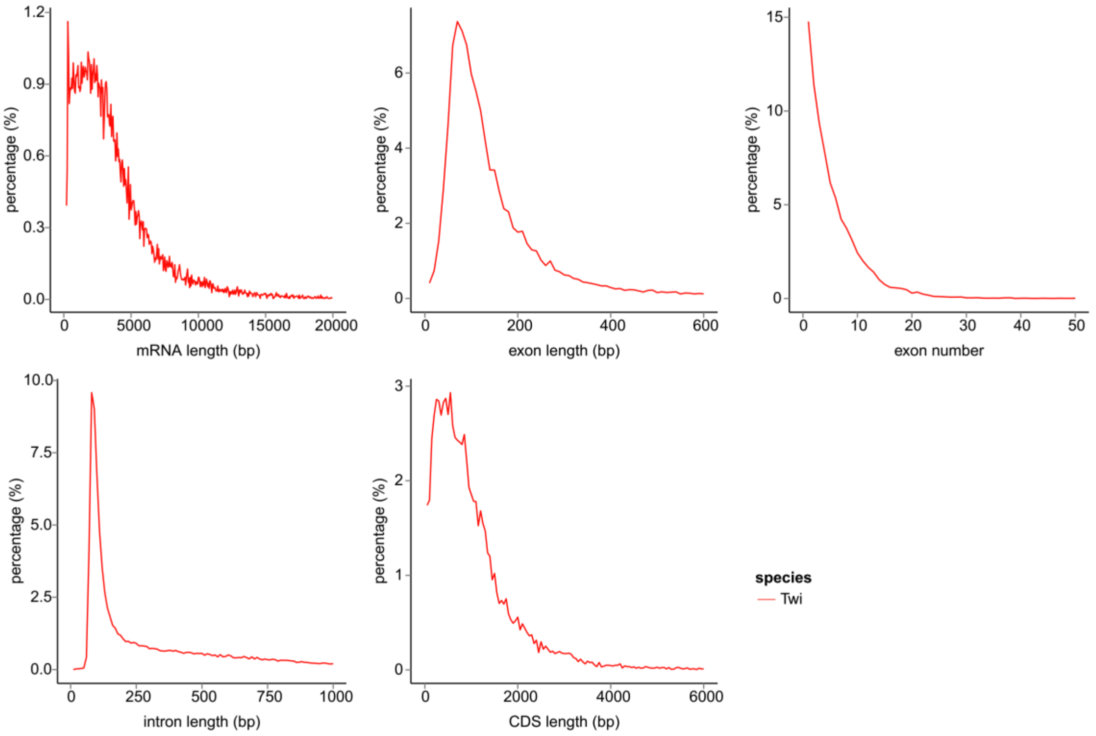

Supplementary Figure 3. Characteristics of predicted *T. wilfordii* protein-coding genes. a, Distribution of mRNA length. **b**, Distribution of exon length. **c**, Distribution of exon number. **d**, Distribution of intron length. **e**, Distribution of CDS length.

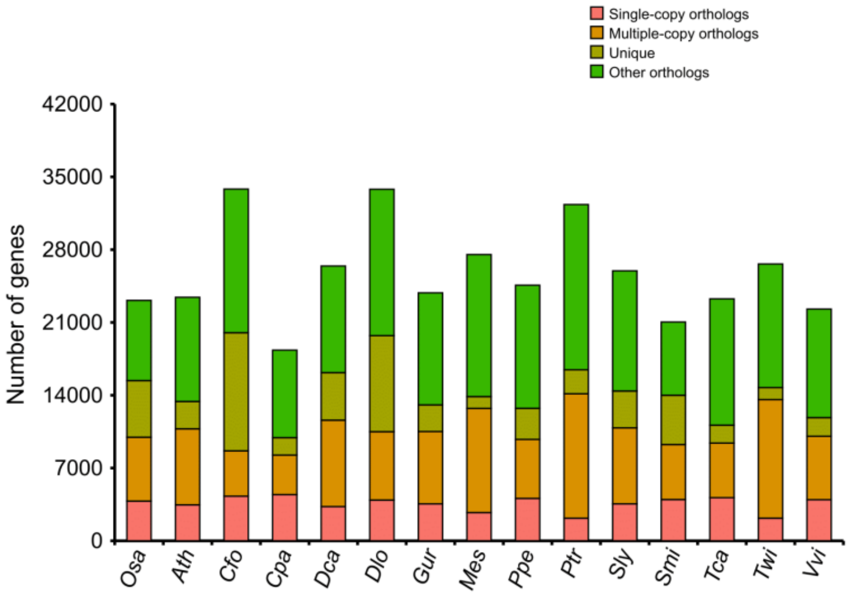

Supplementary Figure 4. The distribution of genes in different species.

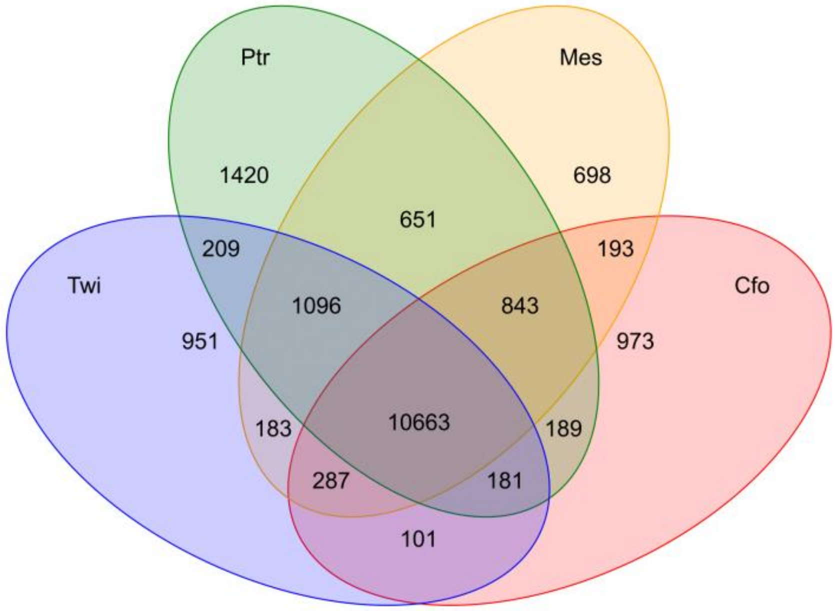

Supplementary Figure 5. Common and unique gene families in four species.

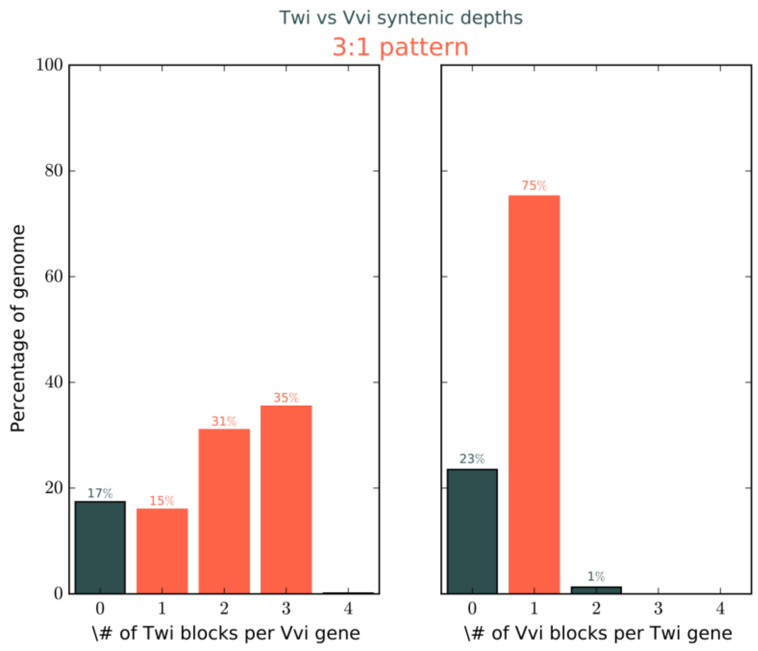

Supplementary Figure 6. Summary of the syntenic analysis between between *T. wilfordii* and *V. vinifera*. n=1 biologically independent samples.

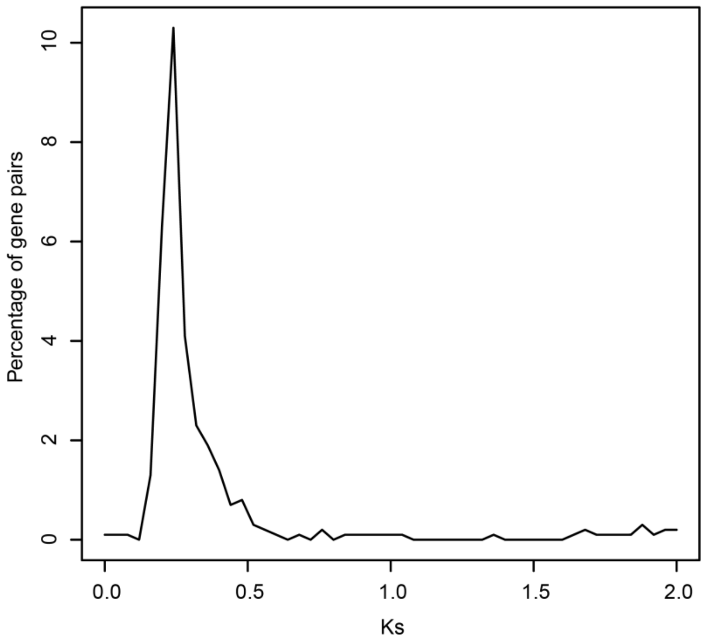

Supplementary Figure 7. Synonymous substitution rate (*Ks*) distributions of syntenic blocks.

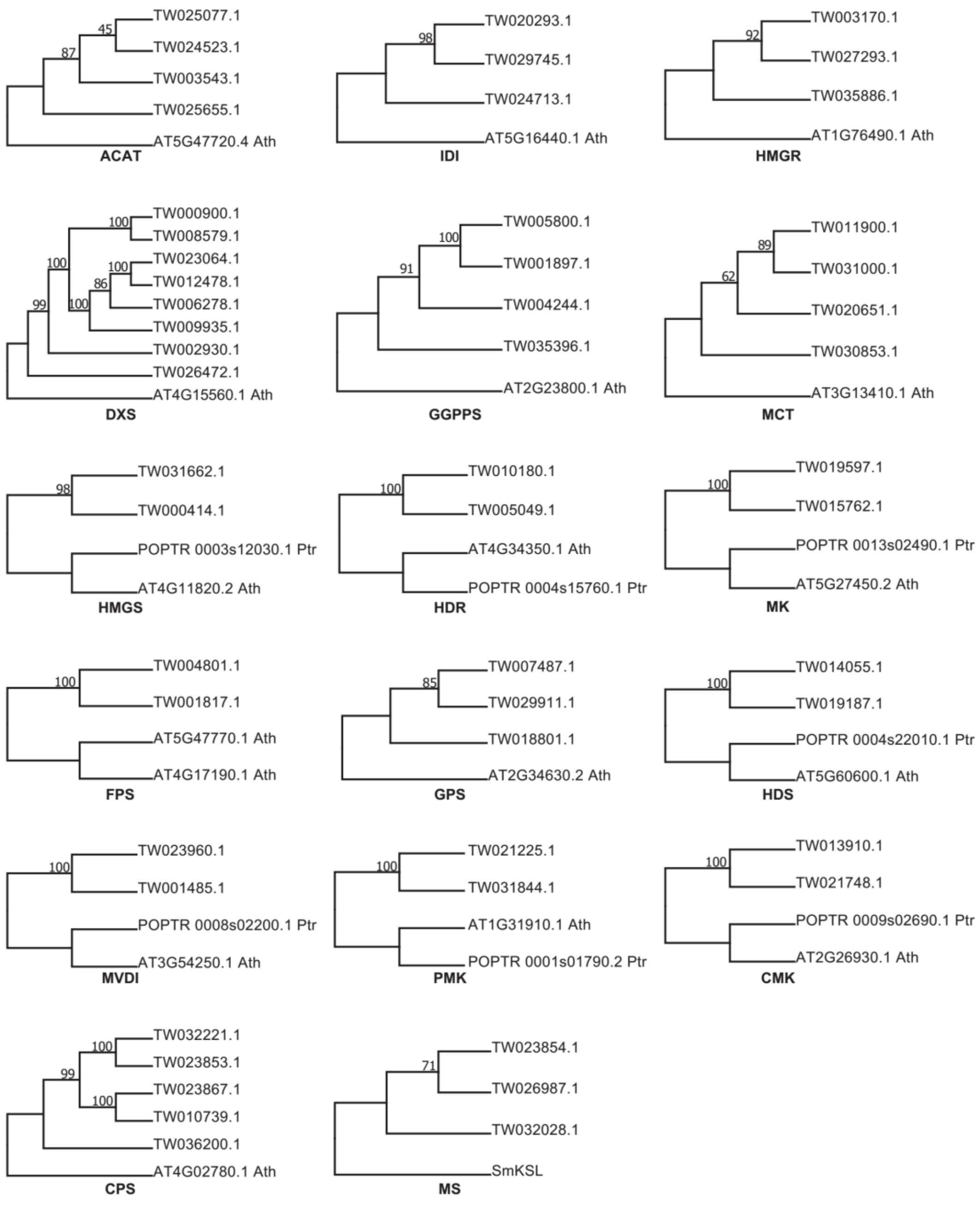

Supplementary Figure 8. Phylogenetic trees of the genes involved in triptolide biosynthetic pathway. Each triptolide biosynthetic gene was construct phylogenetic trees using RAxML with the maximum likelihood method.

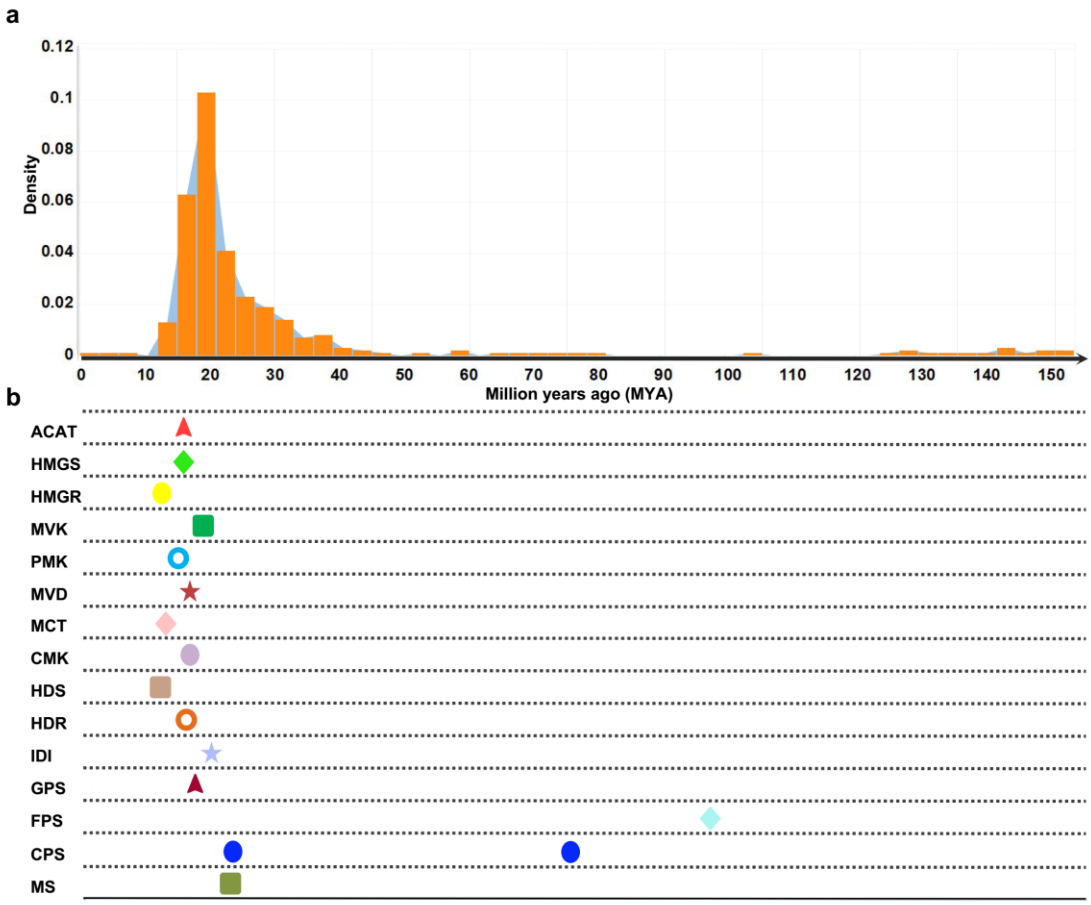

Supplementary Figure 9. Evolution of secondary metabolite-associated genes in *T. wilfordii*. a. Genome duplication in *T. wilfordii*. The calculated Ks value was converted to the divergence time according to T=Ks/2r, where r represents a substitution rate of 6.5×10-9 mutations per site per year for eudicots (n=1 biologically independent samples). **b.** Duplication event(s) for each gene pair is(are) shown along the timeline from 0 to 150 million years ago.

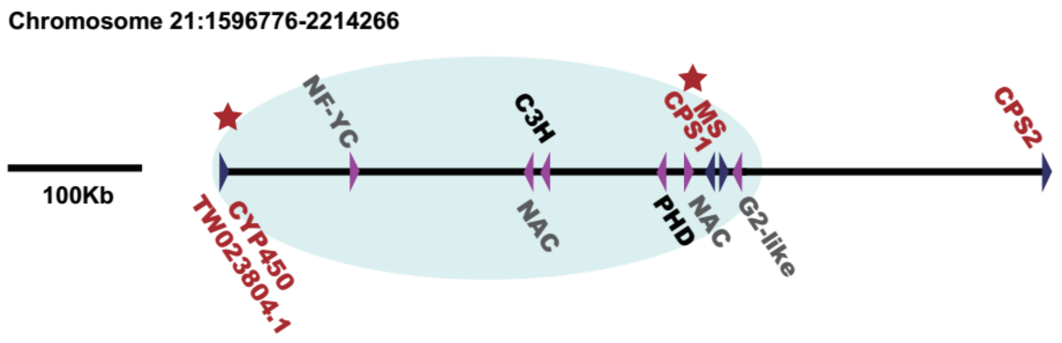

Supplementary Figure 10. Chromosome containing the paired *TwCPS1* and *TwMS* genes involved in triptolide biosynthesis.

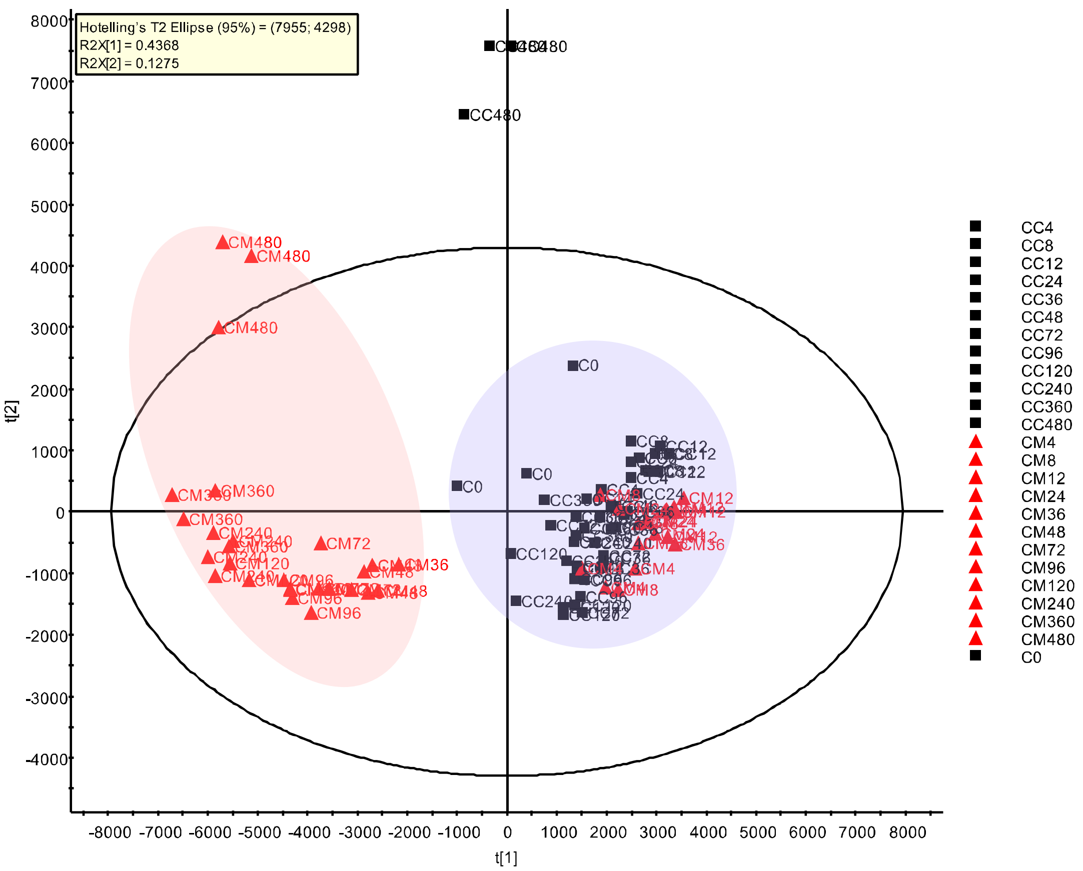

Supplementary Figure 11. Principal component analysis of metabolic profiles in the suspension cells treated with MeJA and DMSO at different times. CC means cell control group, CM means cell MJ-treated group. Mass spectrometry data was analyzed using Progenesis QI and Ezinfo.

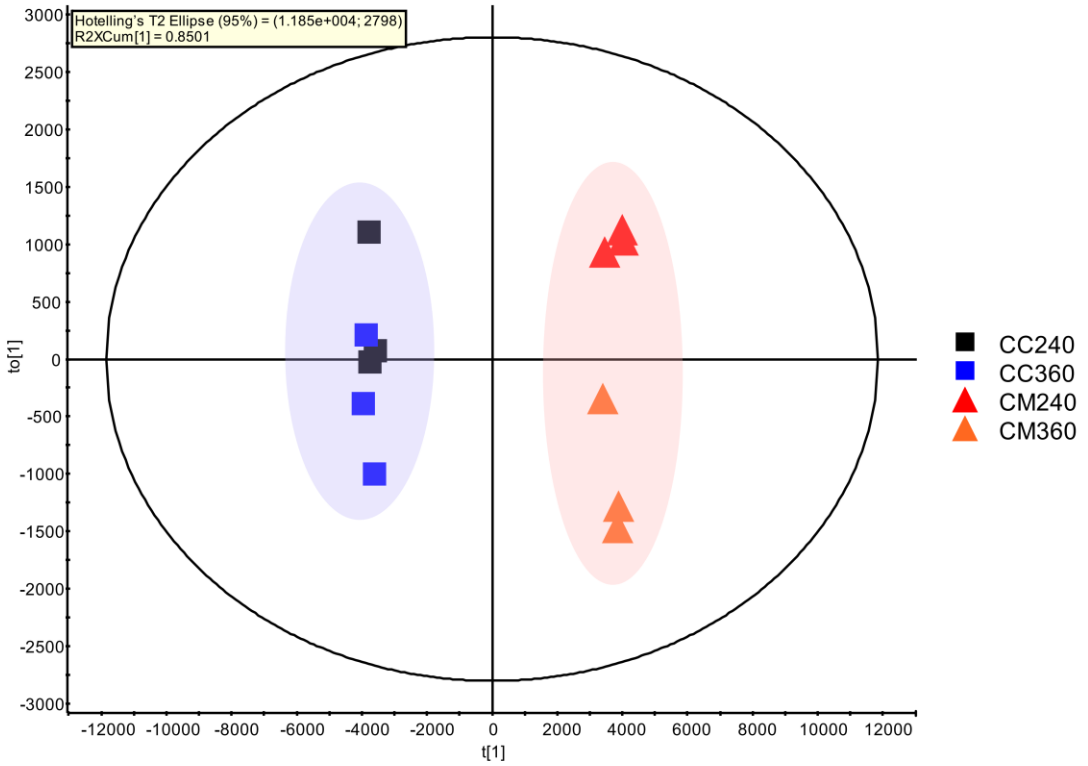

Supplementary Figure 12. The OPLS-DA analysis of metabolic profiles in the suspension cells treated with methyl jasmonate and DMSO at 240-360h. CC means cell control group, CM means cell MJ-treated group. Mass spectrometry data was analyzed using Progenesis QI and Ezinfo.

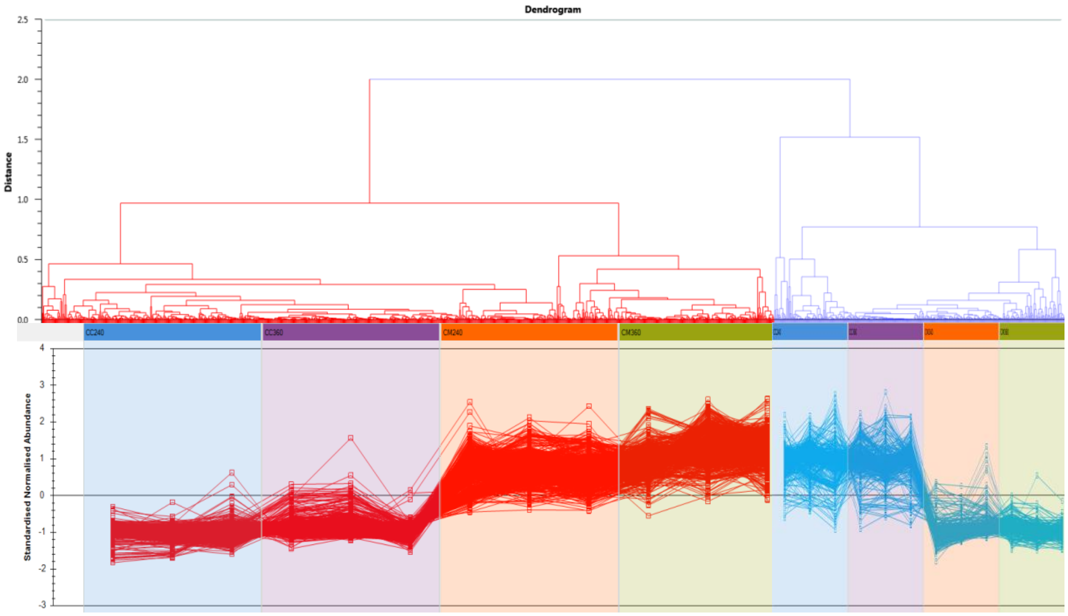

Supplementary Figure 13. 142 peaks with a mass ratio of 295 to 400 obtained from OPLS-DA analysis. Red line represents the up-regulated peak induced by methyl jasmonate and blue line represents the peak of the down-regulation.

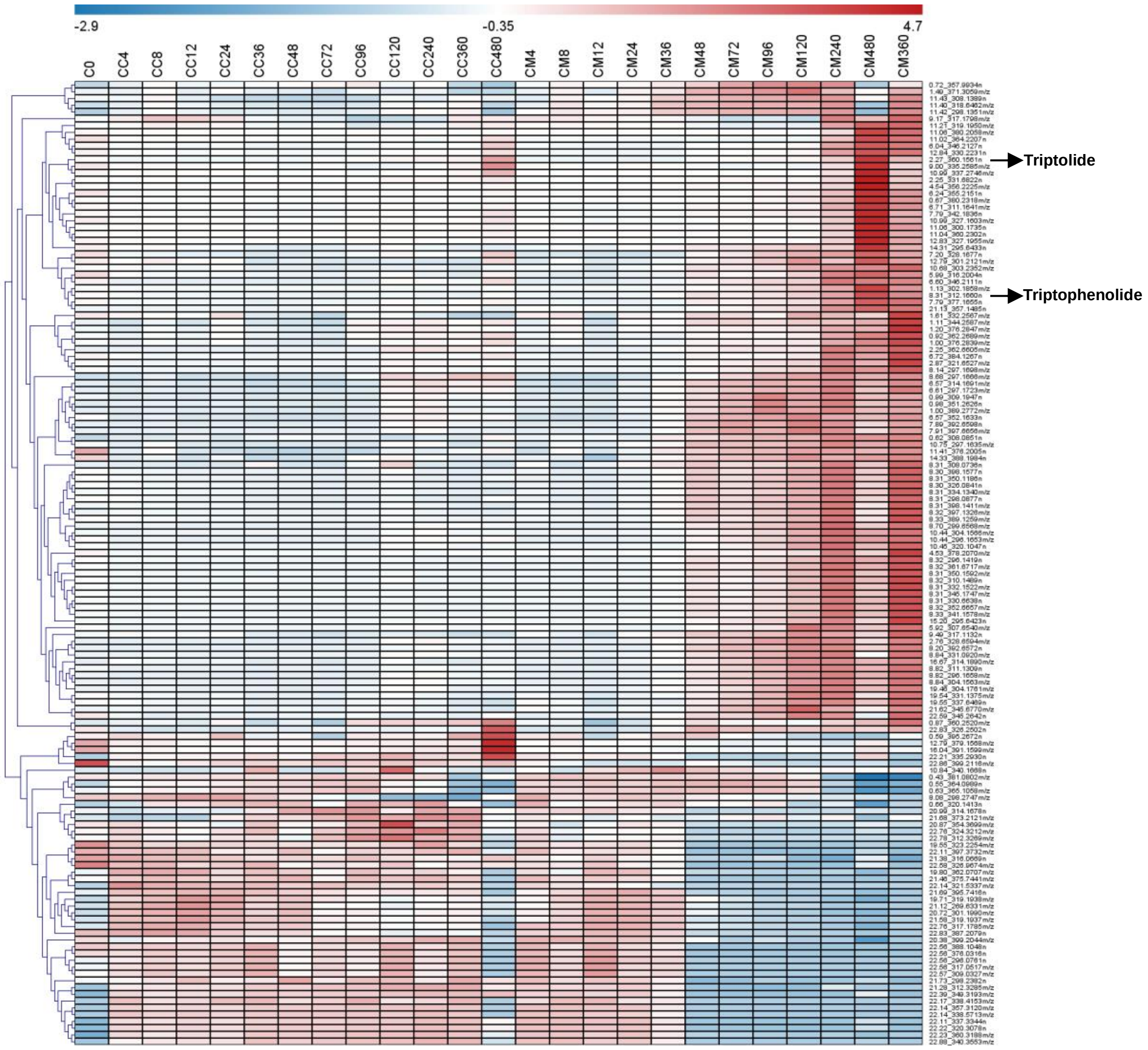

Supplementary Figure 14. Analysis of the content of 142 metabolites at 0-480 hours in the suspension cells. Heat map was plotted using MeV software (version 4.9.0).

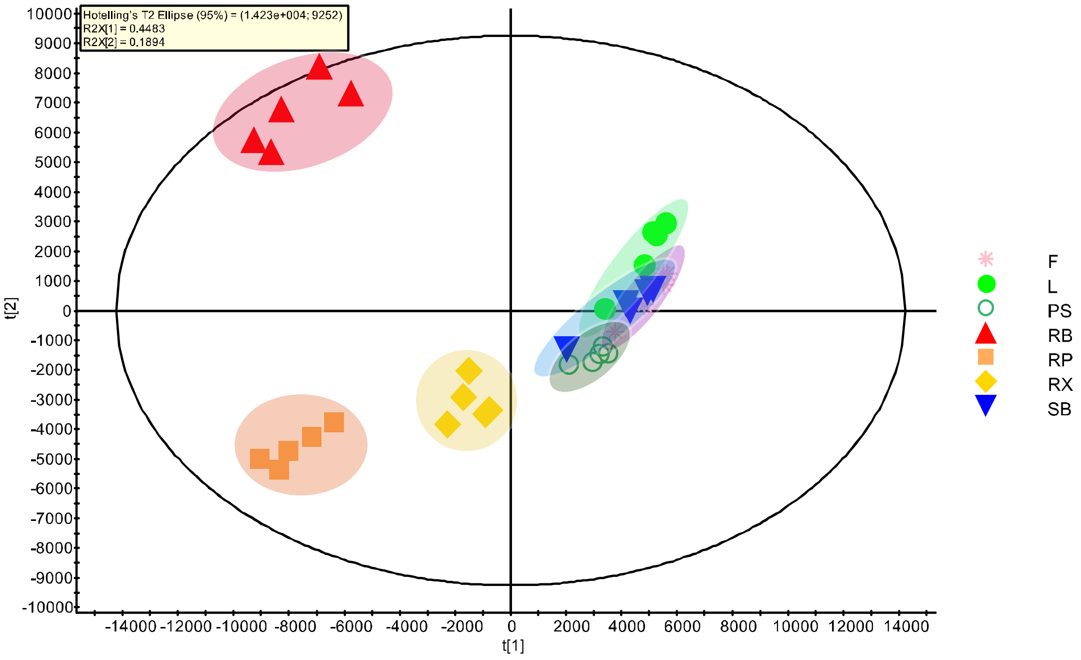

Supplementary Figure 15. Principal component analysis (PCA) of metabolic profiles in seven tissue parts. RB root bark, RP root phloem, RX root xylem, L leaf, F flower, SB stem bark, PS peeled stem. Mass spectrometry data was analyzed using Progenesis QI and Ezinfo.

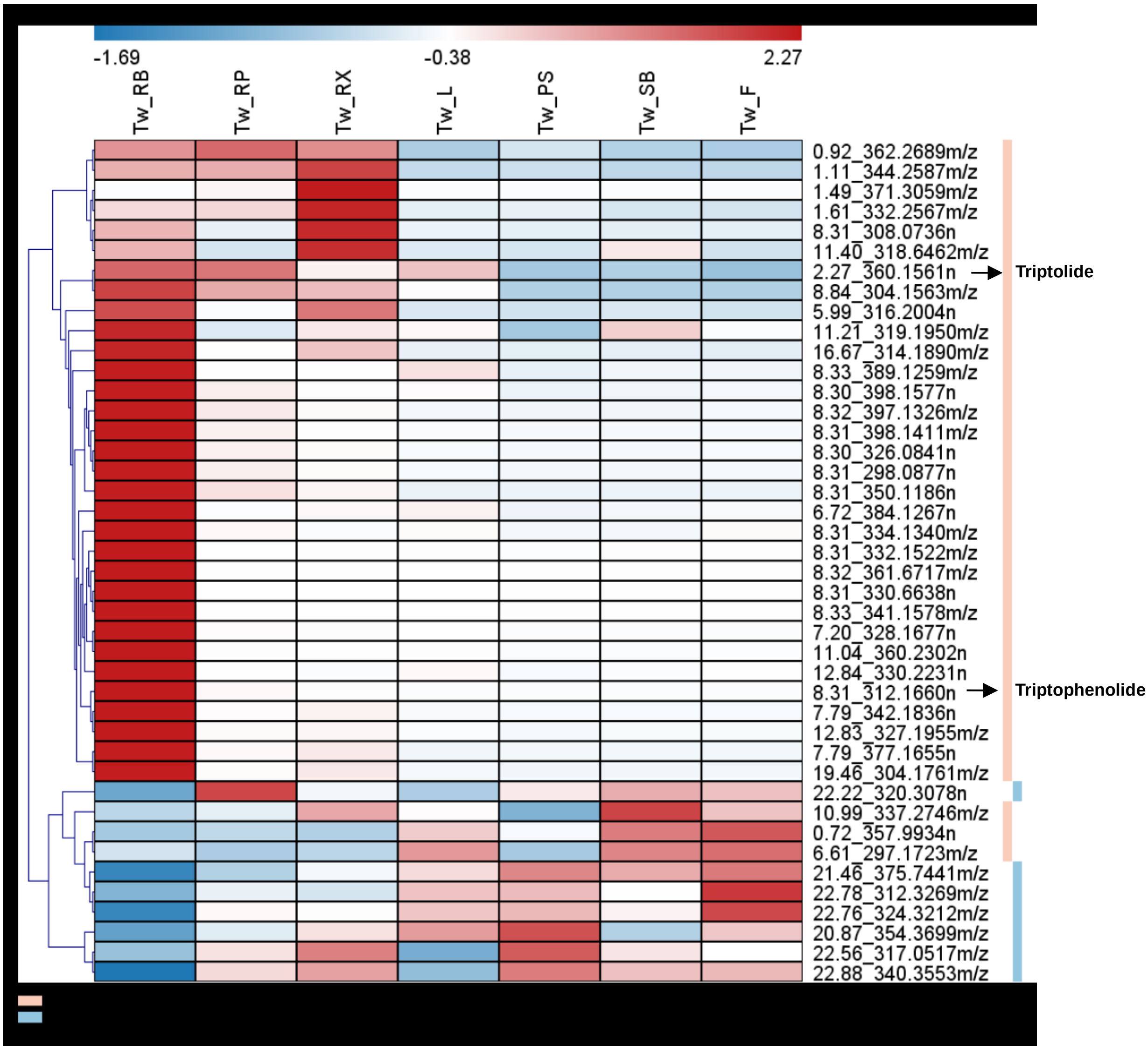

Supplementary Figure 16. Analysis of the content of 42 metabolites in seven tissue parts. Heat map was plotted using MeV software (version 4.9.0).

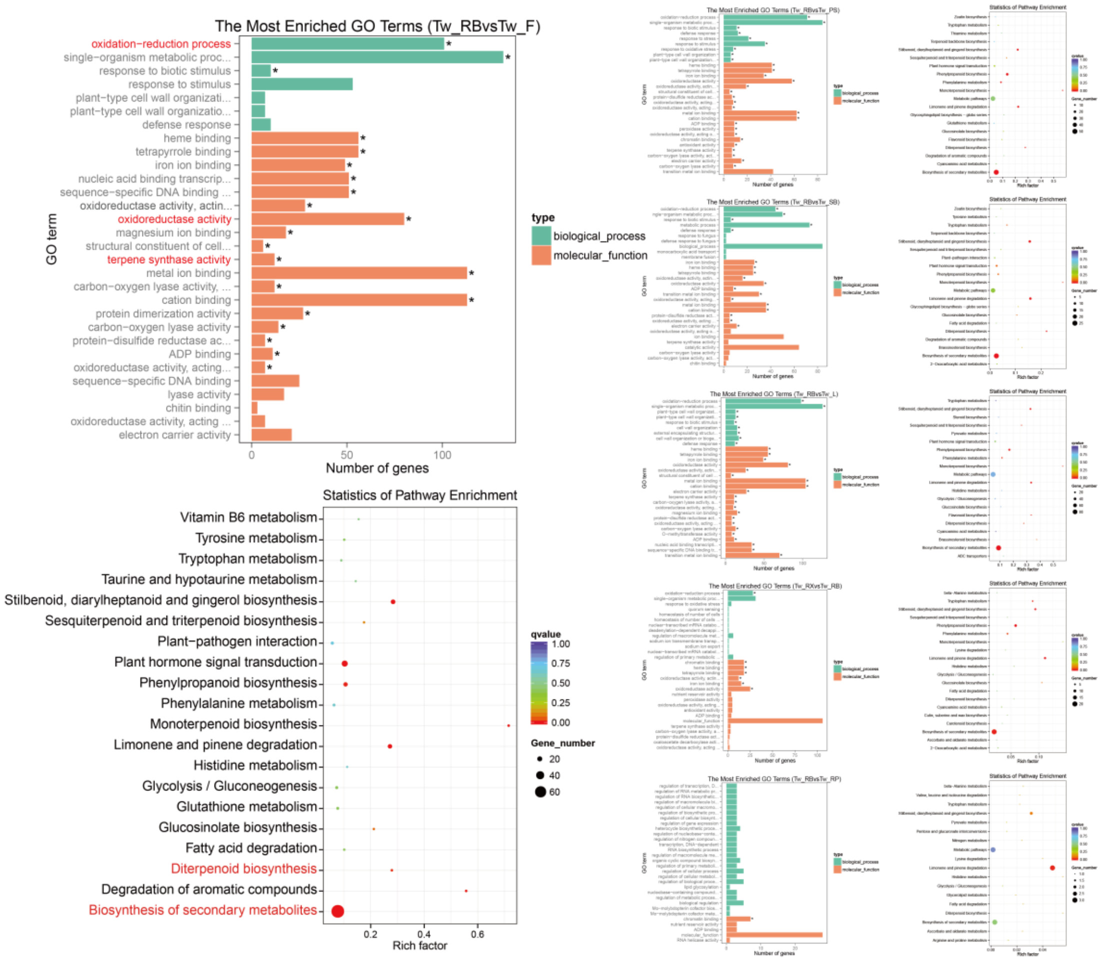

Supplementary Figure 17. GO and KEGG function enrichment of differentially expressed genes in root bark relative to other six tissue parts. * P < 0.05 by hypergeometric test and FDR adjustments.

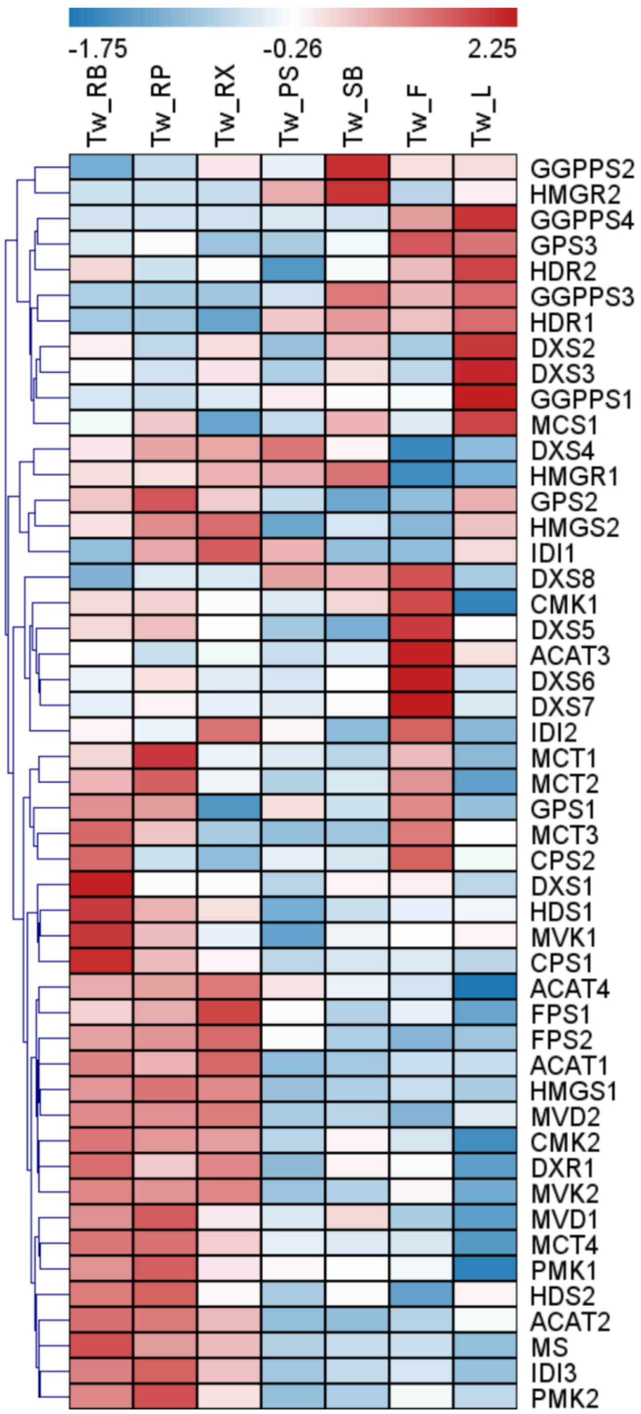

Supplementary Figure 18. Comparative transcriptomic analysis of genes involved in the triptolide biosynthesis among tissues.

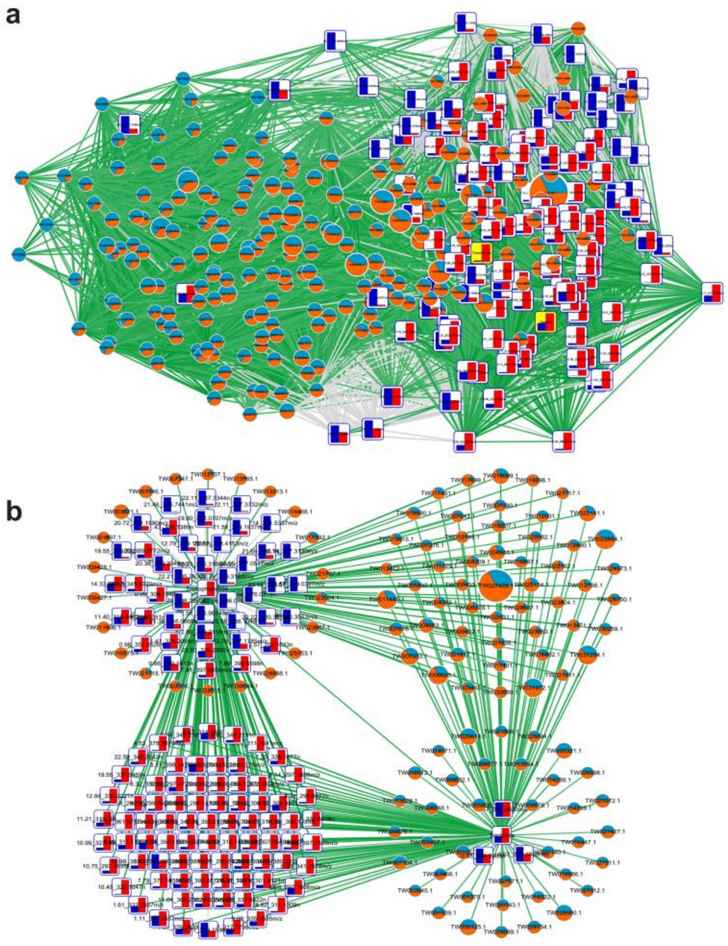

Supplementary Figure 19. Gene-to-metabolite networks in elicited suspension cells. Transcripts are represented by circles and metabolites by squares and edges are drawn when the linear correlation coefficient is > 0.7. The green line represents a positive correlation, the gray line represents a negative correlation, and the darker the line color, the stronger the correlation. The pie chart shows the cumulative expression of genes in the induction group and the control group at 240 h, with orange representing the induction group and blue representing the control group. The size of each pie chart represents the expression level of the gene at 0 h. The histogram represents the relative content of metabolite peaks at 360 h, red for the induction group and blue for the control group. **a**, Correlation network for 142 metabolites and genes consisting of all CYP450s, *TwCPS1* and *TwMS* for suspension cells. **b**, Correlation network only for genes and metabolite peaks directly related to triptolide and triptophenolide in suspension cells.

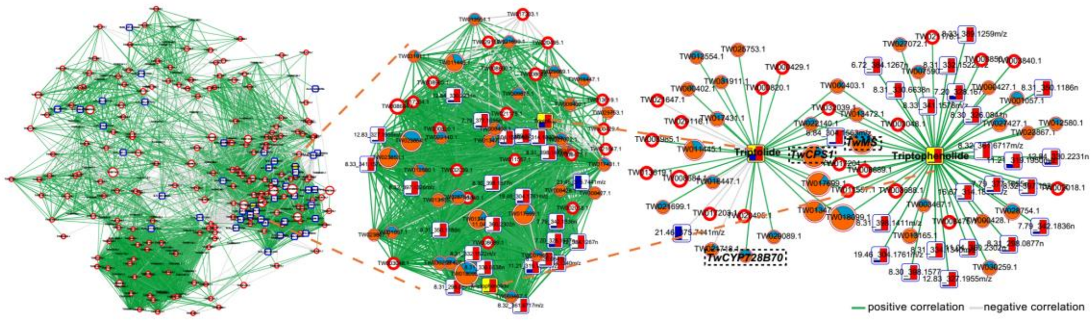

Supplementary Figure 20. Gene-to-metabolite networks in tissues. Transcripts are represented by circles and metabolites by squares and edges are drawn when the linear correlation coefficient is > 0.7 in tissues. The size of each circle represents the expression level of the gene. The pie chart shows the ratio of gene accumulation expression in the induction group and the control group at 240 h, with orange representing the induction group and blue representing the control group. The histogram shows the relative contents of metabolite peaks at 360 h, red for the induction group and blue for the control group.

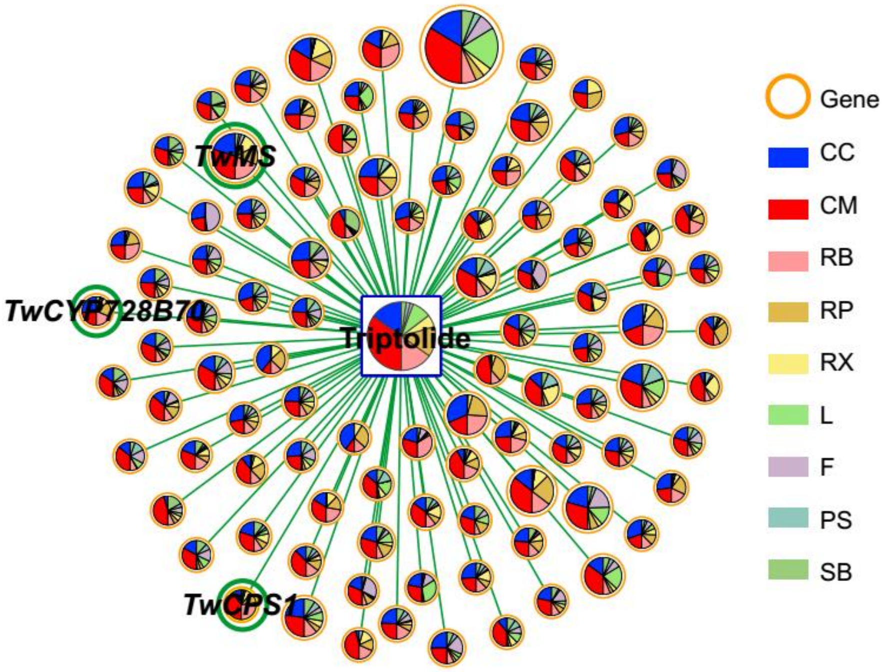

Supplementary Figure 21. Network of 97 CYP genes strongly associated with triptolide in *T. wilfordii*. *TwCPS1* and *TwMS* genes known to be involved in triptolide biosynthesis are also shown. Edges are drawn when the linear correlation coefficient is > 0.7 in tissues and cells. The size of each circle represents the expression level of the gene. The pie chart shows the ratio of relative gene expression or triptolide in different groups, with the left side representing the cells and the right side representing seven tissues. RB, root bark. RP, root phloem. RX, root xylem. L, leaf. F, flower. SB, stem bark. PS, peeled stem

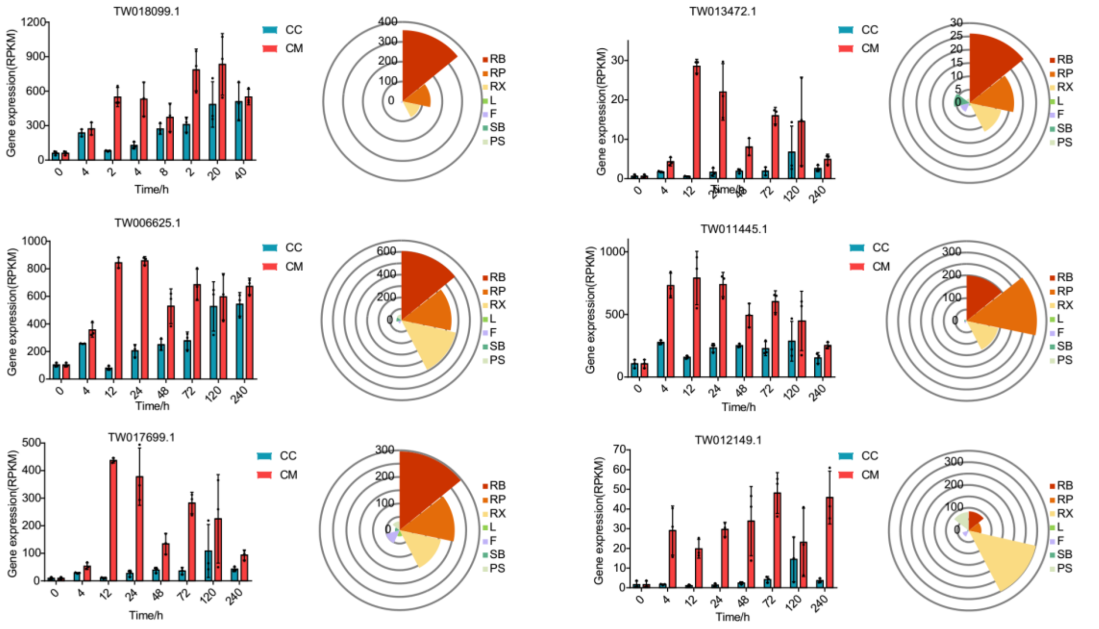

Supplementary Figure 22. The expression profiles of the candidate triptolide-pathway genes of the specific CYP450 subfamily in suspension cell induction experiment and different tissues. RB root bark, RP root phloem, RX root xylem, L leaf, F flower, SB stem bark, PS peeled stem. Error bars, mean +/- SD (n=3 biologically independent samples)

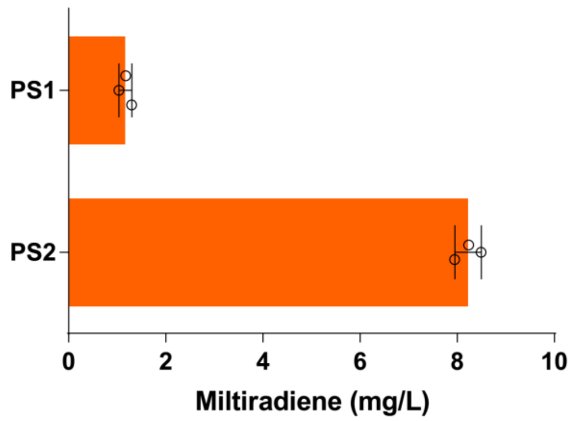

Supplementary Figure 23. Pressing the fusion protein SmMS-SmCPS1. Error bars, mean +/- SD (n=3 biologically independent samples)

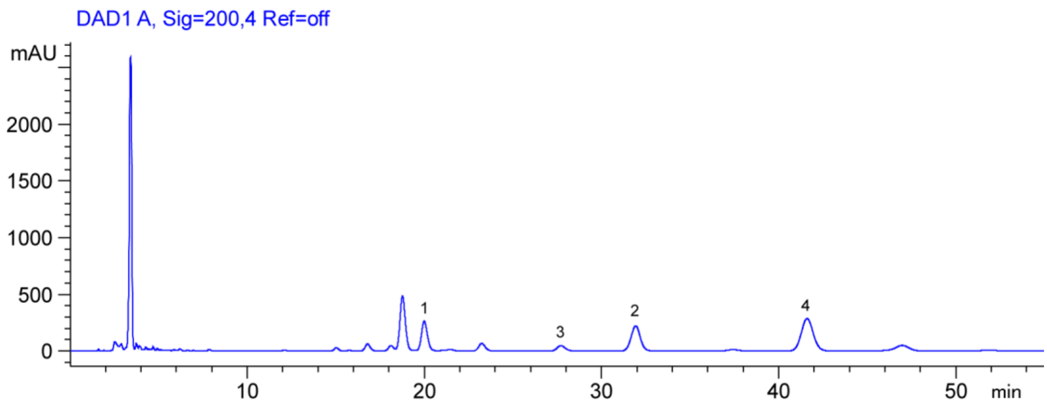

Supplementary Figure 24. Separation and purification of compound 1 to 4.

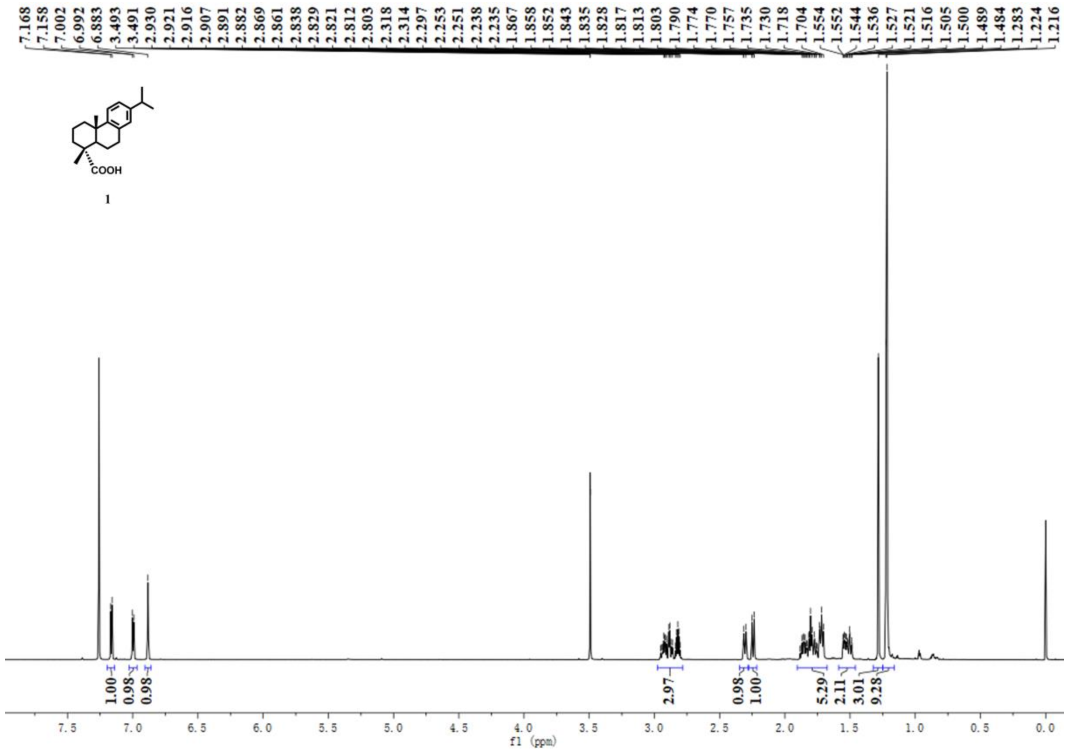

Supplementary Figure 25. ^{1}H NMR spectrum of (1) recorded in chloroform-d at 25 °C.

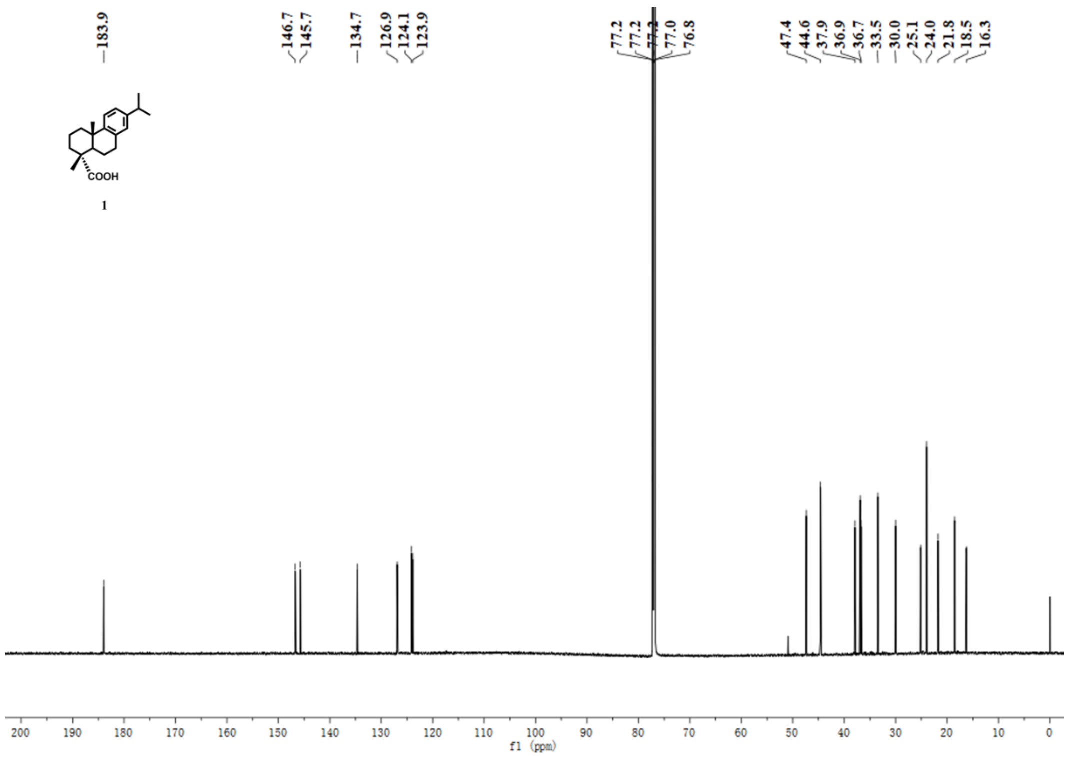

Supplementary Figure 26. ^{13}C NMR spectrum of (1) recorded in chloroform-d at 25 °C.

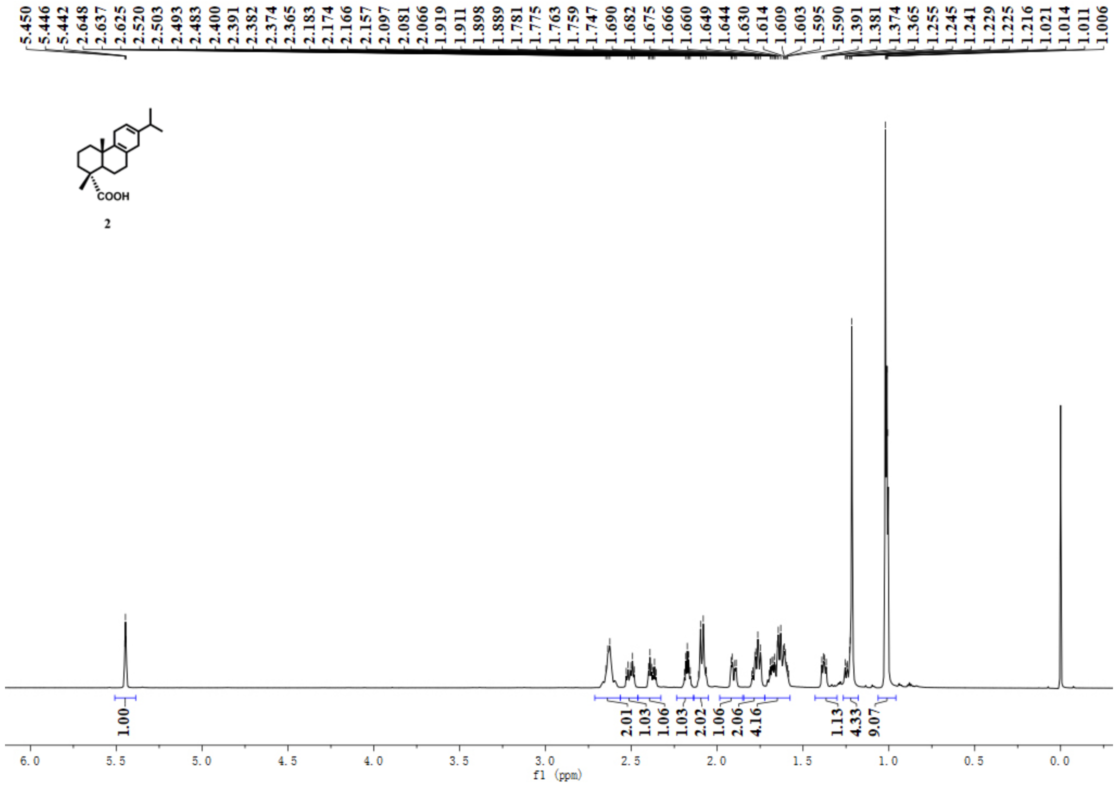

Supplementary Figure 27. ^{1}H NMR spectrum of (2) recorded in chloroform-d at 25 °C.

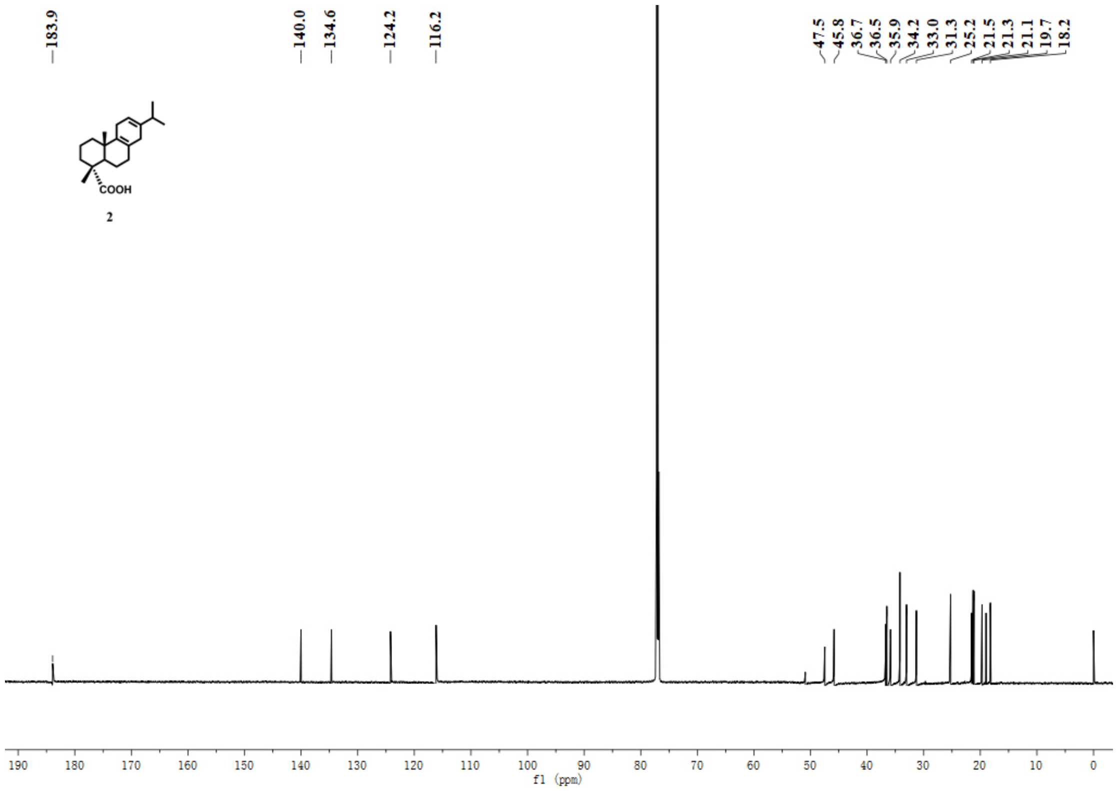

Supplementary Figure 28. ^{13}C NMR spectrum of (2) recorded in chloroform-d at 25 °C.

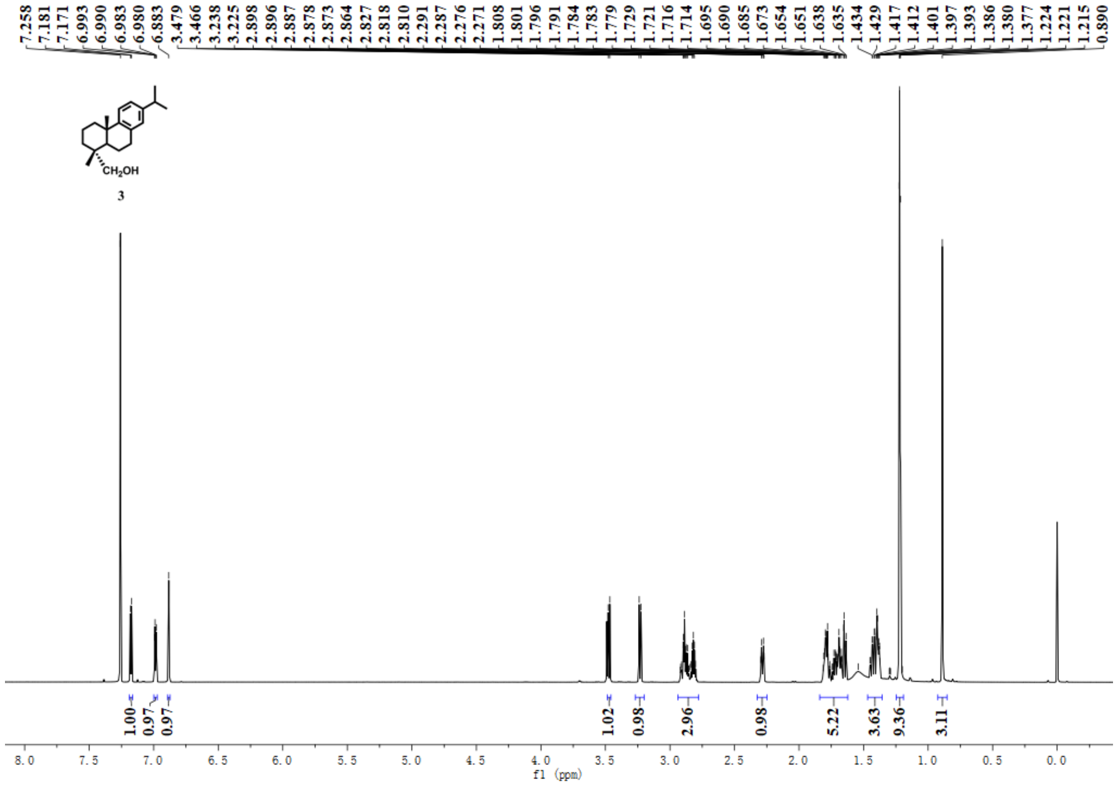

Supplementary Figure 29. ^{1}H NMR spectrum of (3) recorded in chloroform-d at 25 °C.

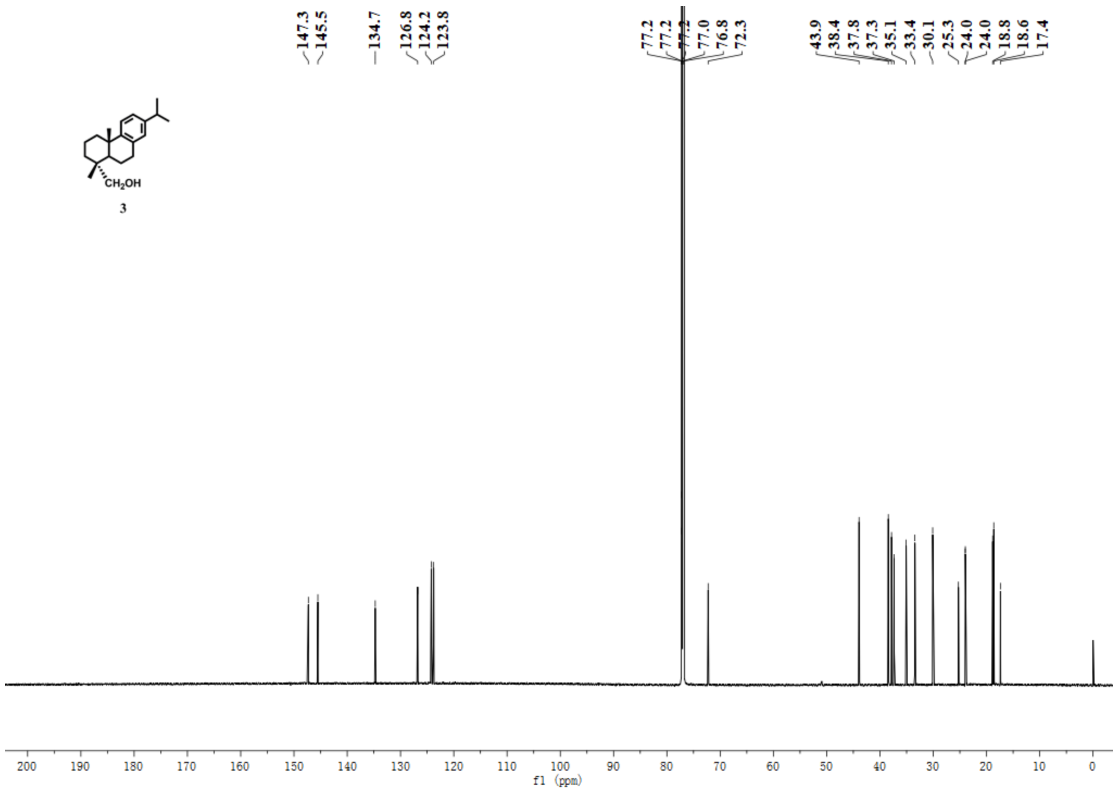

Supplementary Figure 30. ^{13}C NMR spectrum of (3) recorded in chloroform-d at 25 °C.

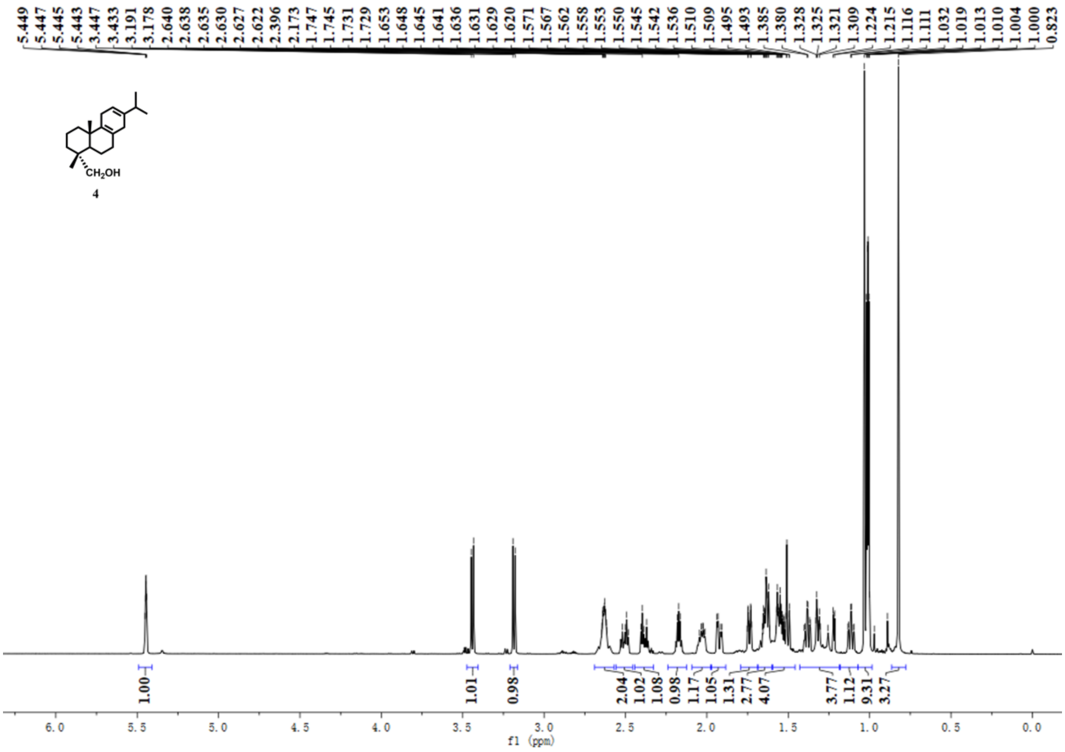

Supplementary Figure 31. ^{1}H NMR spectrum of (4) recorded in chloroform-d at 25 °C.

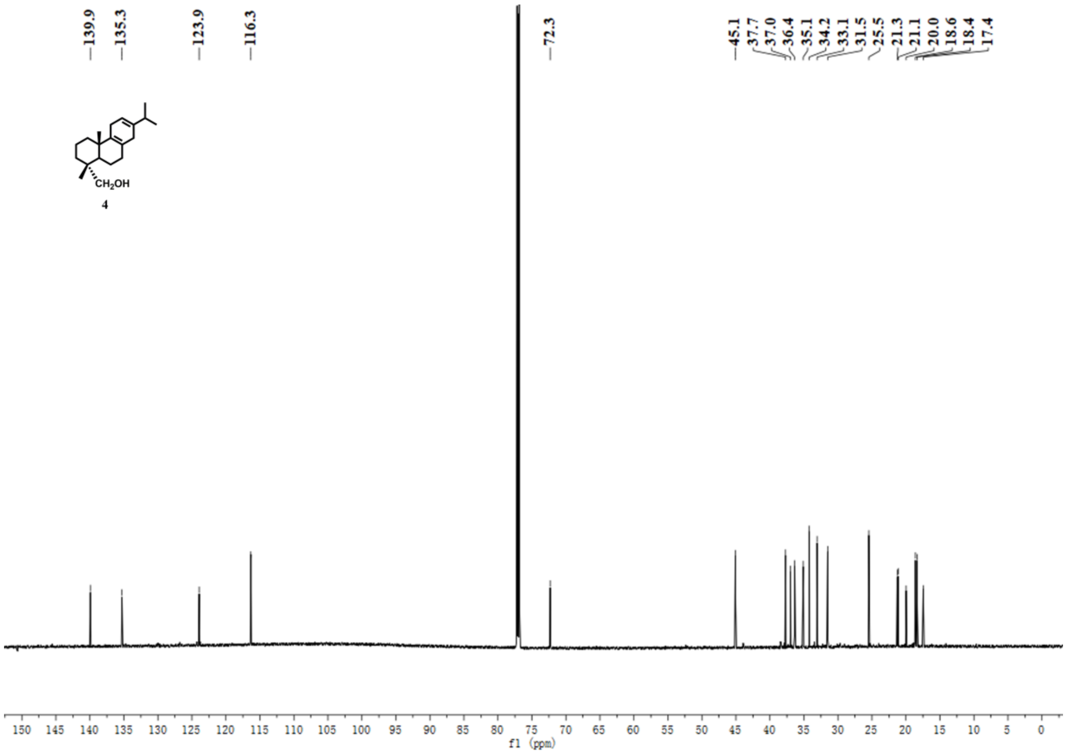

Supplementary Figure 32. ^{13}**C NMR spectrum of (4) recorded in chloroform-d at 25 °C.**

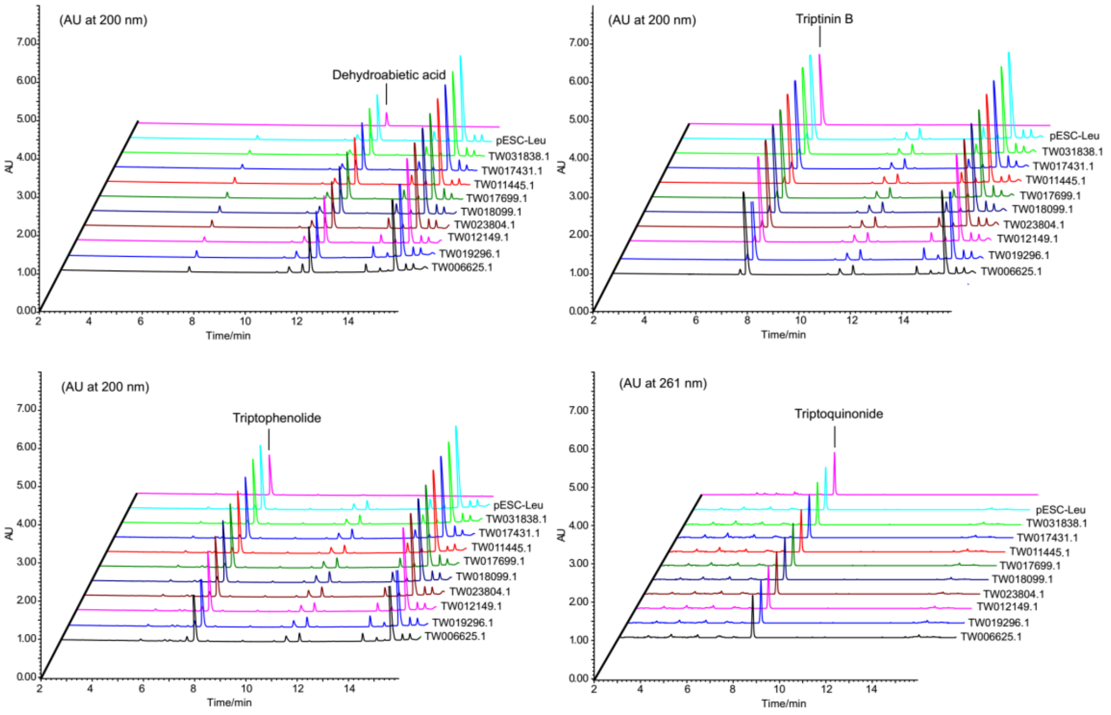

Supplementary Figure 33. ***In vitro*** **enzymatic activity assays**

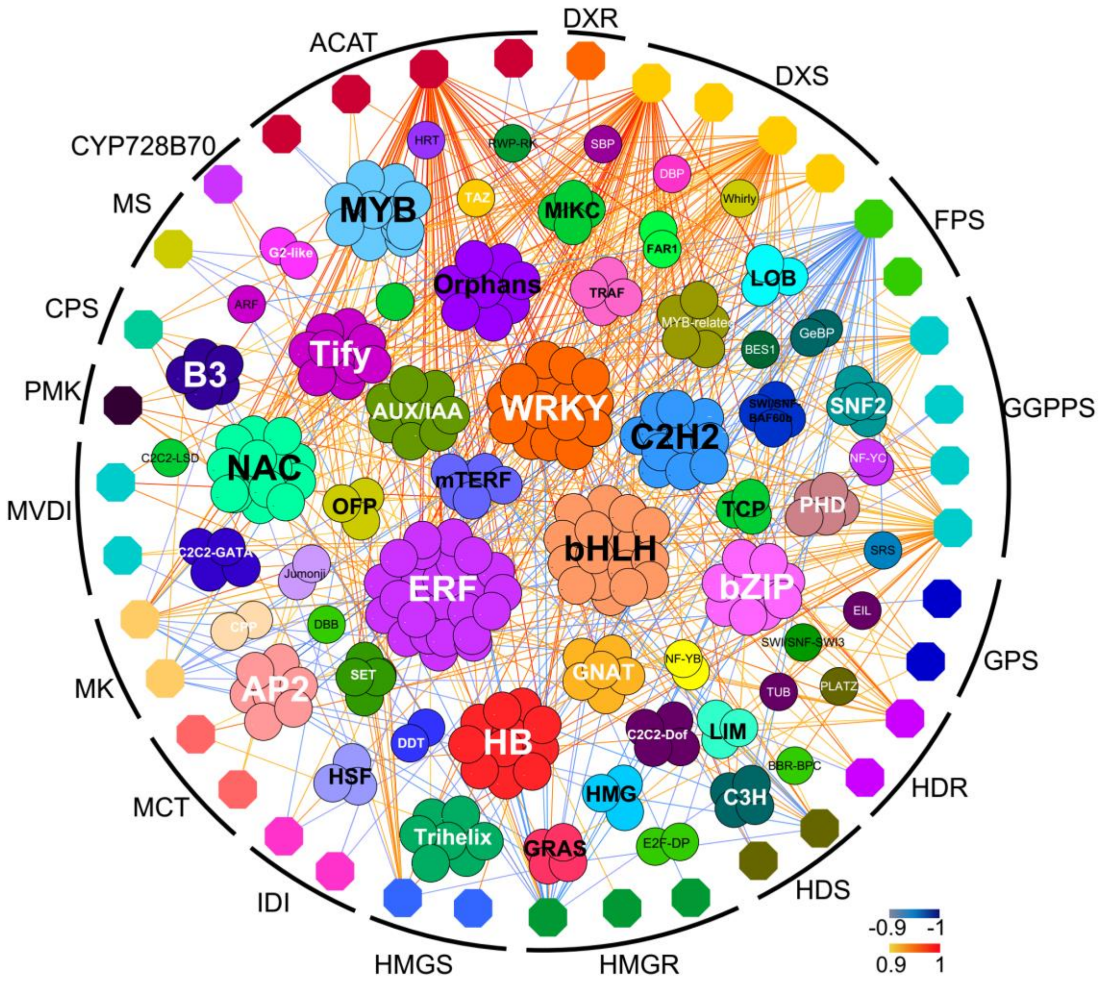

Supplementary Figure 34. Regulatory network of transcription factors and triptolide biosynthetic genes. The octagons represent triptolide biosynthetic genes and the coloured solid circles represent TFs. The edges are drawn when the linear correlation coefficient is > 0.9.

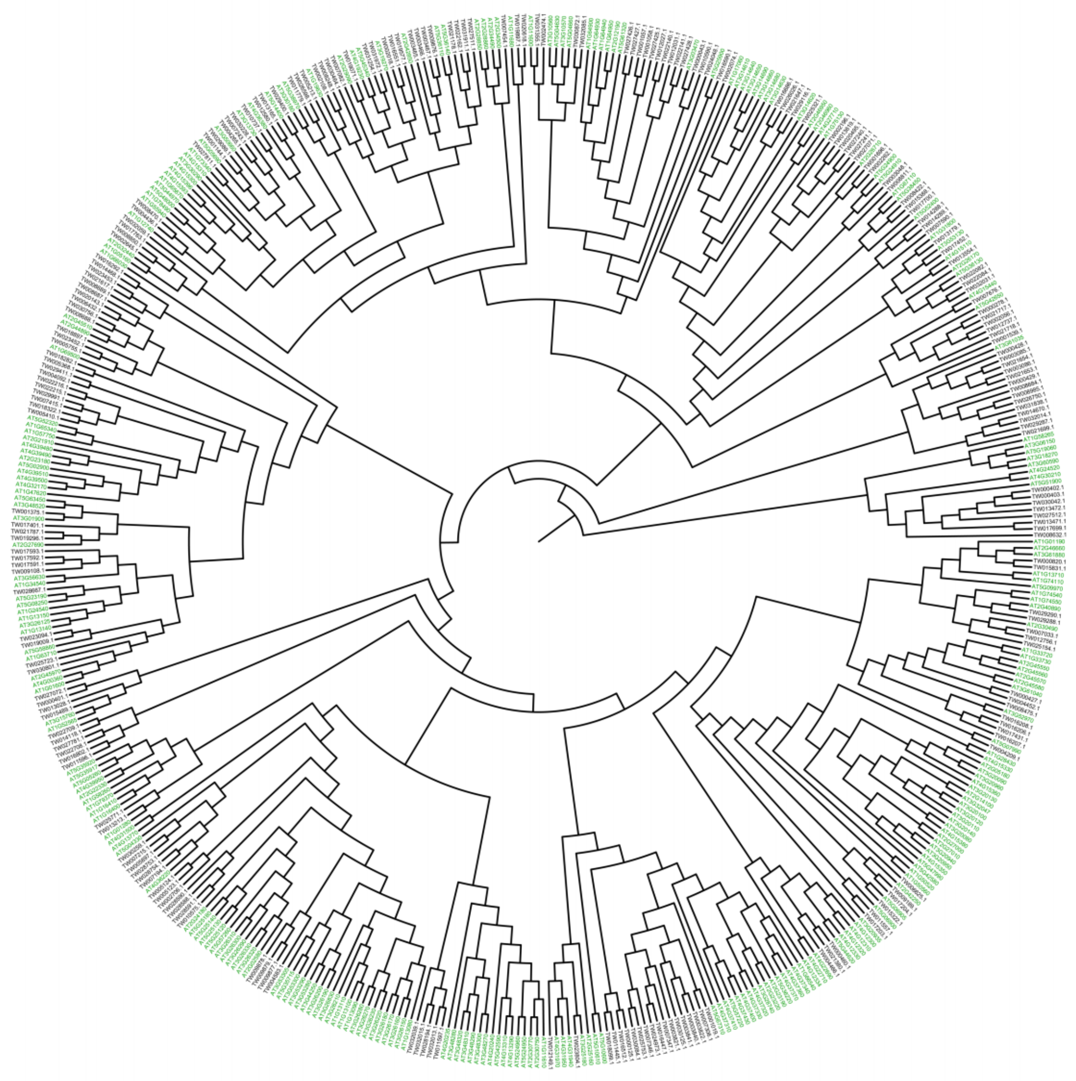

Supplementary Figure 35. Neighbour-joining phylogenetic tree of *Cytochrome P450* genes. *T. wilfordii*: black; *A. thaliana:* green. The NJ tree was constructed using TreeBeST (Version: 1.9.2) with the JTT model and 1000 bootstrap replicates.

Supplementary References

1. Yaffe E, Tanay A. Probabilistic modeling of Hi-C contact maps eliminates systematic biases to characterize global chromosomal architecture. *Nat Genet* **43**, 1059-1065 (2011).

2. Chin CS, *et al.* Phased diploid genome assembly with single-molecule real-time sequencing. *Nat Methods* **13**, 1050-1054 (2016).

3. Chin CS, *et al.* Nonhybrid, finished microbial genome assemblies from long-read SMRT sequencing data. *Nat Methods* **10**, 563-569 (2013).

4. Li H. Toward better understanding of artifacts in variant calling from high-coverage samples. *Bioinformatics* **30**, 2843-2851 (2014).

5. Adey A, *et al.* In vitro, long-range sequence information for de novo genome assembly via transposase contiguity. *Genome Res* **24**, 2041-2049 (2014).

6. Walker BJ, *et al.* Pilon: an integrated tool for comprehensive microbial variant detection and genome assembly improvement. *PLoS ONE* **9**, e112963 (2014).

7. Li H. Aligning sequence reads, clone sequences and assembly contigs with BWA-MEM. Preprint at https://arxiv.org/abs/1303.3997 (2013).

8. Li H, *et al.* The Sequence Alignment/Map format and SAMtools. *Bioinformatics* **25**, 2078-2079 (2009).

9. Grabherr MG, *et al.* Full-length transcriptome assembly from RNA-Seq data without a reference genome. *Nat Biotechnol* **29**, 644-652 (2011).

10. Kent WJ. BLAT - The BLAST-like alignment tool. *Genome Res* **12**, 656-664 (2002).

11. Parra G, Bradnam K, Korf I. CEGMA: a pipeline to accurately annotate core genes in eukaryotic genomes. *Bioinformatics* **23**, 1061-1067 (2007).

12. Simao FA, Waterhouse RM, Ioannidis P, Kriventseva EV, Zdobnov EM. BUSCO: assessing genome assembly and annotation completeness with single-copy orthologs. *Bioinformatics* **31**, 3210-3212 (2015).

13. Benson G. Tandem repeats finder: a program to analyze DNA sequences. *Nucleic Acids Res* **27**, 573-580 (1999).

14. Altschul SF, Gish W, Miller W, Myers EW, Lipman DJ. Basic local alignment search tool. *J Mol Biol* **215**, 403-410 (1990).

15. Yu XJ, Zheng HK, Wang J, Wang W, Su B. Detecting lineage-specific adaptive evolution of brain-expressed genes in human using rhesus macaque as outgroup. *Genomics* **88**, 745-751 (2006).

16. Birney E, Durbin R. Using GeneWise in the Drosophila annotation experiment. *Genome Res* **10**, 547-548 (2000).

17. Trapnell C, Pachter L, Salzberg SL. TopHat: discovering splice junctions with RNA-Seq. *Bioinformatics* **25**, 1105-1111 (2009).

18. Trapnell C, *et al.* Transcript assembly and quantification by RNA-Seq reveals unannotated transcripts and isoform switching during cell differentiation. *Nat Biotechnol* **28**, 511-515 (2010).

19. Campbell MA, Haas BJ, Hamilton JP, Mount SM, Buell CR. Comprehensive analysis of alternative splicing in rice and comparative analyses with Arabidopsis. *BMC Genomics* **7**, 327 (2006).

20. Stanke M, Steinkamp R, Waack S, Morgenstern B. AUGUSTUS: a web server for gene finding in eukaryotes. *Nucleic Acids Res* **32**, W309-312 (2004).

21. Aggarwal G, Ramaswamy R. Ab initio gene identification: prokaryote genome annotation with GeneScan and GLIMMER. *J Biosci* **27**, 7-14 (2002).

22. Majoros WH, Pertea M, Salzberg SL. TigrScan and GlimmerHMM: two open source ab initio eukaryotic gene-finders. *Bioinformatics* **20**, 2878-2879 (2004).

23. Parra G, Blanco E, Guigo R. GeneID in Drosophila. *Genome Res* **10**, 511-515 (2000).

24. Bromberg Y, Rost B. SNAP: predict effect of non-synonymous polymorphisms on function. *Nucleic Acids Res* **35**, 3823-3835 (2007).

25. Haas BJ, *et al.* Automated eukaryotic gene structure annotation using EVidenceModeler and the program to assemble spliced alignments. *Genome Biol* **9**, R7 (2008).

26. Gish W, States DJ. Identification of protein coding regions by database similarity search. *Nat Genet* **3**, 266-272 (1993).

27. Hunter S, *et al.* InterPro: the integrative protein signature database. *Nucleic*

Acids Res **37**, D211-D215 (2009).

28. Finn RD, *et al.* Pfam: the protein families database. *Nucleic Acids Res* **42**, D222-D230 (2014).

29. Quevillon E, *et al.* InterProScan: protein domains identifier. *Nucleic Acids Res* **33**, W116-W120 (2005).

30. Finn RD, Clements J, Eddy SR. HMMER web server: interactive sequence similarity searching. *Nucleic Acids Res* **39**, W29-W37 (2011).

31. Lowe TM, Eddy SR. tRNAscan-SE: a program for improved detection of transfer RNA genes in genomic sequence. *Nucleic Acids Res* **25**, 955-964 (1997).

32. Nawrocki EP, Kolbe DL, Eddy SR. Infernal 1.0: inference of RNA alignments. *Bioinformatics* **25**, 1335-1337 (2009).

33. Griffiths-Jones S, Moxon S, Marshall M, Khanna A, Eddy SR, Bateman A. Rfam: annotating non-coding RNAs in complete genomes. *Nucleic Acids Res* **33**, D121-D124 (2005).

34. Li L, Stoeckert CJ, Roos DS. OrthoMCL: identification of ortholog groups for eukaryotic genomes. *Genome Res* **13**, 2178-2189 (2003).

35. Edgar RC. MUSCLE: multiple sequence alignment with high accuracy and high throughput. *Nucleic Acids Res* **32**, 1792-1797 (2004).

36. Stamatakis A. RAxML version 8: a tool for phylogenetic analysis and post-analysis of large phylogenies. *Bioinformatics* **30**, 1312-1313 (2014).

37. Yang Z. PAML 4: phylogenetic analysis by maximum likelihood. *Mol Biol Evol* **24**, 1586-1591 (2007).

38. Hedges SB, Dudley J, Kumar S. TimeTree: a public knowledge-base of divergence times among organisms. *Bioinformatics* **22**, 2971-2972 (2006).

39. Han MV, Thomas GWC, Lugo-Martinez J, Hahn MW. Estimating gene gain and loss rates in the presence of error in genome assembly and annotation using CAFE 3. *Mol Biol Evol* **30**, 1987-1997 (2013).

40. Tang HB, Bowers JE, Wang XY, Ming R, Alam M, Paterson AH. Perspective - synteny and collinearity in plant genomes. *Science* **320**, 486-488 (2008).

41. Guo L, *et al.* The opium poppy genome and morphinan production. *Science* **362**, 343-347 (2018).

42. Del Sal G, Manfioletti G, Schneider C. The CTAB-DNA precipitation method: a common mini-scale preparation of template DNA from phagemids, phages or plasmids suitable for sequencing. *Biotechniques* **7**, 514-520 (1989).

43. Su P, *et al.* Characterization of eight terpenoids from tissue cultures of the Chinese herbal plant, *Tripterygium wilfordii*, by high-performance liquid chromatography coupled with electrospray ionization tandem mass spectrometry. *Biomed Chromatogr* **28**, 1183-1192 (2014).

44. Anders S, Pyl PT, Huber W. HTSeq-a Python framework to work with high-throughput sequencing data. *Bioinformatics* **31**, 166-169 (2015).

45. Anders S, Huber W. Differential expression analysis for sequence count data. *Genome Biol* **11**, R106 (2010).

46. Young MD, Wakefield MJ, Smyth GK, Oshlack A. Gene ontology analysis for RNA-seq: accounting for selection bias. *Genome Biol* **11**, R14 (2010).

47. Mao XZ, Cai T, Olyarchuk JG, Wei LP. Automated genome annotation and pathway identification using the KEGG Orthology (KO) as a controlled vocabulary. *Bioinformatics* **21**, 3787-3793 (2005).

48. Rischer H, *et al.* Gene-to-metabolite networks for terpenoid indole alkaloid biosynthesis in *Catharanthus roseus* cells. *Proc Natl Acad Sci U S A* **103**, 5614-5619 (2006).

49. Su P, *et al.* Identification and functional characterization of diterpene synthases for triptolide biosynthesis from *Tripterygium wilfordii*. *Plant J* **93**, 50-65 (2018).

50. Fukusaki E, Kobayashi A. Plant metabolomics: potential for practical operation. *J Biosci Bioeng* **100**, 347-354 (2005).

51. Surendra K, Rajendar G, Corey EJ. Useful catalytic enantioselective cationic double annulation reactions initiated at an internal pi-bond: method and applications. *J Am Chem Soc* **136**, 642-645 (2014).

52. Thommen C, Jana CK, Neuburger M, Gademann K. Syntheses of Taiwaniaquinone F and Taiwaniaquinol A *via* an unusual remote C-H functionalization. *Org Lett* **15**, 1390-1393 (2013).

53. Koutsaviti A, Ioannou E, Couladis M, Tzakou O, Roussis V. ^{1}H and ^{13}C NMR spectral assignments of abietane diterpenes from *Pinus heldreichii* and *Pinus nigra* subsp. *nigra*. *Magn Reson Chem* **55**, 772-778 (2017).

54. Woldemichael GM, Wachter G, Singh MP, Maiese WM, Timmermann BN. Antibacterial diterpenes from *Calceolaria pinifolia*. *J Nat Prod* **66**, 242-246 (2003).

55. Gonzalez MA, *et al.* Synthesis and biological evaluation of dehydroabietic acid derivatives. *Eur J Med Chem* **45**, 811-816 (2010).

56. Yang XW, *et al.* Isolation, structure, and bioactivities of abiesadines A-Y, 25 new diterpenes from *Abies georgei* Orr. *Bioorg Med Chem* **18**, 744-754 (2010).

57. Shannon P, *et al.* Cytoscape: a software environment for integrated models of biomolecular interaction networks. *Genome Res* **13**, 2498-2504 (2003).

58. He X, Wang H, Yang J, Deng K, Wang T. RNA sequencing on *Amomum villosum* Lour. induced by MeJA identifies the genes of WRKY and terpene synthases involved in terpene biosynthesis. *Genome* **61**, 91-102 (2018).

59. Hahlbrock K, *et al.* Non-self recognition, transcriptional reprogramming, and secondary metabolite accumulation during plant/pathogen interactions. *Proc Natl Acad Sci U S A* **100 Suppl 2**, 14569-14576 (2003).

60. Wei CL, *et al.* Draft genome sequence of *Camellia sinensis* var. *sinensis* provides insights into the evolution of the tea genome and tea quality. *Proc Natl Acad Sci USA* **115**, E4151-E4158 (2018).

61. Zhang CP, *et al.* Studies on diterpenoids from leaves of *Tripterygium wilfordii*. *Yao Xue Xue Bao* **28**, 110-115 (1993).

62. Ehlting J, Sauveplane V, Olry A, Ginglinger JF, Provart NJ, Werck-Reichhart D. An extensive (co-)expression analysis tool for the cytochrome P450 superfamily in *Arabidopsis thaliana*. *BMC Plant Biol* **8**, 47 (2008).

63. Dai Z, *et al.* Producing aglycons of ginsenosides in bakers' yeast. *Sci Rep* **4**, 3698 (2014).

64. Dai Z, *et al.* Metabolic engineering of *Saccharomyces cerevisiae* for production of ginsenosides. *Metab Eng* **20**, 146-156 (2013).
