# Supplementary material for: Genome of Tripterygium wilfordii and identification of cytochrome P450 involved in triptolide biosynthesis
Source: Nat Commun. 2020 Feb 20;11:971. doi: 10.1038/s41467-020-14776-1 (PMC7033203; doi:10.1038/s41467-020-14776-1)
Supplement: Supplementary file 4 — Description of Additional Supplementary Files [file 41467_2020_14776_MOESM4_ESM.docx]

**Description of Additional Supplementary Files**

File Name: Supplementary Data 1

Description: Summary of *T.wilfordii* pseudomolecules.

File Name: Supplementary Data 2

Description: Gennomic location of genes involved in triptolide biosynthesis in *T. wilfordii*.

File Name: Supplementary Data 3

Description: Correlation analysis of genes and metabolites involved in triptolide biosynthetic pathway in induction experiment.

File Name: Supplementary Data 4

Description: Correlation analysis of genes and metabolites involved in triptolide biosynthetic pathway in tissues.

File Name: Supplementary Data 5

Description: 228 P450 genes in *T. wilfordii* genome and functional annotation.

File Name: Supplementary Data 6

Description: Statistical analysis of differential expression of P450s.

File Name: Supplementary Data 7

Description: All primers used in this study.

File Name: Supplementary Data 8

Description: Transcription factors (TF) across sequenced plant genomes.

File Name: Supplementary Data 9

Description: Correlation analysis of transcription factors that are highly associated with triptolide biosynthetic genes.

File Name: Supplementary Data 10

Description: Statistical analysis of the numbers of transcription factors highly associated with triptolide biosynthesis genes.

File Name: Supplementary Data 11

Description: Statistical analysis of differential expression of transcription factor.

File Name: Supplementary Data 12

Description: Statistical time of up-regulation of transcription factors induced by MeJA.

File Name: Supplementary Data 13

Description: Statistical time of down-regulation of transcription factors induced by MeJA.
