# Supplementary material for: Genome of Tripterygium wilfordii and identification of cytochrome P450 involved in triptolide biosynthesis
Source: Nat Commun. 2020 Feb 20;11:971. doi: 10.1038/s41467-020-14776-1 (PMC7033203; doi:10.1038/s41467-020-14776-1)
Supplement: Supplementary file 2 — Peer Review File [file 41467_2020_14776_MOESM2_ESM.pdf]

Reviewers' comments:

Reviewer #1 (Remarks to the Author):

Review of manuscript entitled “Multi-omic analysis of Tripterygium wilfordii provides a foundation for investigation of triptolide biosynthesis”

Tu et al. developed a multi-omic approach to elucidating biosynthetic pathways, called MAEBLC, and applied it to the production of triptolide in the perennial shrub Tripterygium wilfordii. There were two major challenges. The low abundance of triptolide implies that relevant enzymes may be encoded by genes with correspondingly low expression levels. Previous work had identified the relevant diterpene synthases, but subsequently acting enzymes like cytochrome P450s (CYPs) remained enigmatic as the complexity of this gene family makes it difficult to infer catalytic function by sequence homology alone. Notable progress was made, as follows.

To begin, they generated a de novo genome sequence, with N50 scaffold size of 1.03 Mb, 96% CEGMA coverage of core eukaryotic genes, and 94% BUSCO coverage of single-copy orthologs. These are all respectable numbers, indicating a high-quality genome assembly by contemporary standards. Overall statistics for annotated protein-coding genes are also in line with what has been reported for other plant genomes. Unfortunately, the evolutionary analysis conveyed by Figure 1 is sloppily described. 1a has an obvious typo: P. richocarpa should be P. trichocarpa. More distressingly, they claim peaks are seen at 0.36 and 0.51 in their T. wilfordii genome, but that is not what is seen in the green distribution labeled Twi_Twi. Either the figure is mislabeled or the manuscript is wrong, but it is not my job as a reviewer to correct the mistakes of the authors. That said, it is not essential for a paper on the MAEBLC method to correctly describe the evolutionary history of T. wilfordii.

Triptolide formation was induced by addition of methyl jasmonate (MeJA), which showed a 3.6-fold increase relative to control cultures after 360h; increase in the level of triptophenolide was even higher, with a 55-fold effect. Pearson correlation coefficients were computed between each set of variables (metabolite or gene), including all conditions and time points, to identify candidate enzymes for further analysis. Because triptolide is found only in Tripterygium, some of the relevant CYPs are likely to be specific to T. wilfordii. Phylogenetic analysis of 2335 CYP genes from multiple species revealed 16 genes that satisfied their uniqueness criteria. The expression levels for six of these genes were significantly increased by MeJA induction, especially in root bark, where triptolide and/or triptophenolide formation was most abundant. By some unknown criteria that needs to be explained, they then expanded their candidates list from 6 to 10 CYPs.

RNAi was used to narrow the candidates list, searching for instances where transcript levels of the targeted gene and triptolide accumulation were decreased compared to control cultures transformed with an empty vector. This gave them a list of 4 CYPs, of which CYP728B70 (strongly associated with triptolide in the gene-metabolite network) was the most promising. It generated four new compounds identified by 1H and 13C NMR spectrums. Overexpression of CYP728B70 produced a 70% increase in triptolide levels, relative to control cultures. As for the other 9 candidates, in vitro enzymatic assays did not reveal the production of likely triptolide biosynthesis intermediates. No new compounds were observed. Contrary to the claims in the introduction that some important enzymes might be expressed at levels too low to detect, this does not appear to be the case for CYP728B70. Nevertheless, it is a member of a very large and complex gene family, and catalytic function cannot be characterized purely by homology. The fact they succeeded is an achievement worth reporting.

Transcription factors (TFs) that regulate triptolide biosynthesis were identified by correlation analysis of gene expression changes induced by MeJA. Not much in the way of novel insight was offered, in part because transcription regulation is, in general, poorly understood. As an aside, Figure 5 was cited before Figure 4, and this should be corrected.

It is fair to say the authors have done a lot of work, providing the first genome sequence for a plant of the order Celastrales, and characterizing the catalytic function for a cytochrome P450 (CYP728B70) that is clearly important for triptolide biosynthesis. However, they fell notably short of the ultimate goal, to reconstitute the biosynthetic pathway in a heterologous organism. On the other hand, they did engineer yeast to make dehydroabietic acid, arguably an important intermediate goal.

Lastly, their figures are much too complex; especially Figure 3, where the details are so small they are basically illegible. Given the inevitable word limits, it would be better if the authors would decide what is essential, devote more wording in their captions to explain what is depicted, and relegate any omitted materials to the online supplements.

Reviewer #2 (Remarks to the Author):

The authors designed a multi-omics workflow to investigate triptolide biosynthesis in Tripterygium wilfordii. With comparative transcriptomics analysis, RNA interference in suspension cells and heterologously expression of genes in engineered yeast, the authors identified TwCYP728B70, which was shown to trigger consecutive oxidations at C-18 of miltiradiene or abietatriene to form the corresponding alcohol and carboxylic acid derivatives, which may be biosynthetic precursors of triptolide.

Although the manuscript represents a compilation of great amount of work (including the genome sequence of Tripterygium wilfordii), it did not yield significant advance in our understanding of triptolide biosynthesis. At this point, the multi-omics-guided workflow for studying plant secondary metabolism highlighted in this paper has become well-established in the field. The fact that the whole approach only identified one gene potentially involved in triptolide biosynthesis suggests such approach is less effective than what the authors claimed. The genome sequencing effort did not seem to help gene discovery for triptolide biosynthesis. Folding it into this paper renders this potentially valuable data set not adequately explored. The genome-wide analysis of transcription factors without functional testing is also not relevant to the story. Some of my specific comments are below.

Specific comments:

1. When the authors extracted metabolites from MJ-treated cells, “60 mg aliquots were soaked in 1.5 mL of 80% (v/v) methanol overnight at room temperature and then dissolved in an ultrasonic water bath for 60 min”. It is not clear why the authors decided to do overnight extraction. Unstable intermediates might not be preserved under such protocol.

2. It is noted that, when the authors characterizedTwCYP728B70 in yeast, a different extraction and detection method were employed (in this case GC-MS). Would this suggest the original 80% methanol extraction coupled with the UPLC/Q-TOF MS analysis might miss the intermediates derived from miltiradiene? If this were the case, the “gene-to-metabolite” analysis would be greatly limited. It is obvious, even without any experiments, that CYPs will be involved. Had the authors just performed RNA-seq analysis of the suspension cells and searched for T. wilfordii specific CYPs, TwCYP728B70 would nevertheless stand out. The point here is that the power of the multi-omics approach might be greatly limited due to the experimental design/analytic capacity.

3. In the overexpression experiment described in Fig. 3f, it is perplexing that the level of triptolide suddenly increased at day 9, given that the transcription level of TwCYP728B70 remained constant from day 5 to day 9. Also, the increased expression of TwCYP728B70 was only observed in day 3 and that quickly dropped to the basal level for the rest of the experiment (all the way to day 9). These observations should be discussed.

4. The authors tested the functions of the other 9 candidate CYPs by in vitro microsome assay. The microsome assay can be complicated. Some control experiments should be included to show that active CYPs are present. The substrates tested do not seem to be water soluble. Did the authors prepare the stock solution in organic solution and then add to the assays? If that were the case, would the addition of organic solvent affect the enzyme activity? Did the substrate remain dissolved?

5. Details of extraction and purification procedure of compound 1 to 4 should be included.

Reviewer #3 (Remarks to the Author):

This is an interesting study describing the elegant elucidation of a biosynthetic reaction en route to the bioactive diterpene triptolide and possibly related compounds. This study follows prior work on the characterization of diterpene synthases for triptolide biosynthesis from Tripterygium wilfordii (Su et al. 2018 Plant Journal 93:50-65). In the present study, the authors generated a draft genome of Tripterygium wilfordii, providing deeper insight into the divergence of diterpene-metabolic genes in this species. Further transcriptomics- and metabolomics-enabled gene discovery was used in combination with RNAi and biochemical enzyme characterization to uncover a new cytochrome P450 that generates dehydroabietic acid, a likely intermediate in the triptolide pathway. I appreciate the multidisciplinary approach, using enzymology, -omics, and phylogenetics employed here, which is timely and state of the art. The functional involvement of a newly characterized P450 (of a family not previously associated with terpenoid metabolism) in triptolide synthesis has been comprehensively demonstrated and is well presented.

Comments for the authors:

- While I appreciate this study, I believe the presentation would benefit from toning down the overstatement and hype. Most notably, the authors present the approach taken here as a novel strategy (termed MAEBLC). While clearly cutting edge, correlation of combined omics approaches for gene-metabolite network analysis has been used in prior studies and does not per se present a newly thought-out strategy. The authors should consider rephrasing the corresponding statements. Along the same lines, the authors state that this strategy now enables the discovery of pathways that are ‘currently intractable’ (line 333), which is an overstatement given the increasing number of studies demonstrating different successful approaches for identifying species-specific secondary metabolite pathways. Likewise, the authors describe triptolide as having ‘remarkable antitumor activities’ and ‘a new blockbuster’ drug’. Again, while triptolide clearly has important therapeutic potential as indicated by (pre-)clinical trials, it is far from being an approved drug. The relevance of the target metabolite should be stated in this context.

- The authors identify 10 P450 candidates based their gene-metabolite network analysis and characterize one as a terpenoid-metabolic P450 of the CYP728 family. Did the other candidates belong to the same family or to P450 families previously shown to be involved in terpenoid-metabolism? A more detailed description of the identified candidates would provide a more comprehensive picture of the divergence of P450s in Tripterygium and the spectrum of P450 families with possible roles in this pathways.

- The description of identified transcription factor networks is somewhat superficial and reads like a late addition to the study. While knowledge of the regulatory elements controlling secondary metabolite pathways is of great importance, a deeper analysis would be valuable here. For example, a genomic and/or phylogenetic analysis of select TF candidates with those identified in other plant species would already provide a deeper insight into possible candidates for future studies.

- Figure 3 a: The figure is very hard to read and does not provide much insight beyond highlighting the complexity of the network. A clearer presentation, for example, buy highlighting relevant correlation would be helpful.

Minor comments:

- A minor suggestion for the title: 'foundation for the investigation of' or 'foundation for investigating'

- Line 57: the contaminating impact of Tripterygium pollen on honey should be described in more detail.

- Line 57: 'at' a large scale

- Line 79: Original works on the identification of triptolide-metabolic diterpene synthases should cited (Su et al. 2018 Plant Journal 93:50-65; Hansen et al. 2017 Plant Journal 89:429-41).

- Line 97: It would be helpful to describe the novel de novo assembler in more detail here. What is the advantage over previously established approaches?

- Line 148: GGPPS instead of GGPS?

- Line 187: Tripterygium should be in italics

- Line 240: 'intermediates' instead of 'intermeidates'

Reviewer #4 (Remarks to the Author):

In the presented research article by Tu et al., titled, "Multi-omic analysis of Tripterygium wilfordii provides a foundation for the investigation of triptolide biosynthesis", authors reported a multi-omics approach to elucidating the biosynthesis of low-abundance compounds (MAEBLC). Authors presented highly heterozygous genome of T. wilfordii. Authors then used chemical induced cell suspension culture to perform time-series transcriptome and metabolome analysis, and integrative omics analysis to identify CYP728B70 and further functionally characterized it. This is a well-written manuscript and flow of this study is easy to follow. This study does advances resource for medicinal plants which is extremely important, at this stage, I do feel that it lacks the strength to match quality expected from Nature communication. My assessments are based on following point-

1. Throughout the manuscript, authors describe about the multi-omics approach to elucidating the biosynthesis of low-abundance compounds (MAEBLC) but I do not this is new approach, but rather same as what has been used for many years. Authors used transcriptome and metabolome correlation analysis for a time series dataset, and used genome comparison to identify genes specific to their target plant. I think this is a common classical practice and not enough to emphasize so much in this article. In fact, One of the reason for me to review this article was after reading this article but was disappointment after completing this article.
2. The first two result section of this manuscript is genome assembly and characterization of Tripterygium wilfordii genome. While I agree that this is an important resource, the quality of final genome assembly for me is not impressive and probably the only week point of this study. Authors assembled this genome with scaffold and contig N50 as 1.3Mb and 28Kb, respectively. This genome seems highly fragmented, which is little disappointing since even Mb levels contigs are becoming a norm these days. Total number of contigs and scaffolds are 90681and 73038, respectively. I strongly feel that authors could do much better job on this. I recommend to probably add more long read

sequencing datasets and test different parameters to get final assembly. Since their primary assembly is fragmented, they could not get a high quality genome by using 10x data. Wheat genome for instance have very similar sequencing strategy as this plant but were able to achieve an amazing genome assembly at the end.
3. I not agree with authors snteny analysis and interpretation, given such a fragmented genome for this species. Authors in line no-135 states, "Intergenomic
co-linearity analysis was consistent with both the γ-event and another, more specific WGD event for T. wilfordii, as indicated by a 1:2 syntenic relationship between T. wilfordii and Vitis vinifera (Supplementary Fig. 7, Supplementary Data 1).". How authors can say this, one can see multiple syntenic regions from V. vinifera getting connected at multiple sites for their species. Further, I am assuming that authors simply selected scaffolds of certain size to o this analysis and not entire genome. If this is the case, authors need to mention that.
4. Authors description about integrated transcriptome and metabolome analysis is impressive and data is good for sure. I think this is good enough to predict functional genes for characterization. Indeed, the 10 genes identified by authors were induced by JA treatment and highly expressed in root bark. So, genome analysis although does support this, but really not essential. That's one of the reason why I expected high genome assembly quality as this allows to look at many different aspects of evolution and gene clusters that could serve as a resource for future discoveries.
5. Authors described whole genome duplication for their species, and did described on genes related to terpenoid biosynthesis. The conclusion was that duplication of involved genes in this plant species occurred post whole genome duplication. Since the genome quality is not good, unfortunately, authors were not able to make statement if these genes showed locational duplication? This is important aspect for the evolution of especially metabolites and a big gap that authors missed to address here.
6. I am curious about association between metabolome and transcriptome association. I understand that authors used correlation to identify genes co-expressed with their metabolite of interest. But as we know, JA treatment causes induction of genes and the response, which is change in metabolite levels, takes more time to appear. Is this the same case in their study? What are the early and late responsive genes in response to JA treatment? How about transcription factors? Is it possible that transcription factors that are induced at the early time point could regulate genes expressed at late time point, some of which involved in triptolides biosynthesis. There are many such interesting questions that authors if try to address will improve this manuscript significantly.
7. I am also curious about levels of the newly identified four metabolites in response to CYP728B70, these metabolites were not identified or induced in response to JA treatment even post 240 hrs post treatment?

Response to reviewers

Reviewer #1

Reviewer 1 Comment 1
Figure 1a has an obvious typo: P. richocarpa should be P. trichocarpa. More distressingly, they claim peaks are seen at 0.36 and 0.51 in their *T. wilfordii* genome.

Response: We thank the reviewer for catching these mistakes, which have been corrected. (line 138)

Reviewer 1 Comment 2
Phylogenetic analysis of 2335 CYP genes from multiple species revealed 16 genes that satisfied their uniqueness criteria. The expression levels for six of these genes were significantly increased by MeJA induction, especially in root bark, where triptolide and/or triptophenolide formation was most abundant. By some unknown criteria that needs to be explained, they then expanded their candidates list from 6 to 10 CYPs.

Response: We apologize for the lack of detail. A more complete description of our criteria for screening of CYPs has been added.

Reviewer 1 Comment 3
Contrary to the claims in the introduction that some important enzymes might be expressed at levels too low to detect, this does not appear to be the case for CYP728B70. Nevertheless, it is a member of a very large and complex gene family, and catalytic function cannot be characterized purely by homology. The fact they succeeded is an achievement worth reporting.

Response: We thank the reviewer for the kind suggestion. We have revised the Introduction section in an attempt to emphasize the difficulties of CYP characterization and embody the achievement in our work.

Reviewer 1 Comment 4

Transcription factors (TFs) that regulate triptolide biosynthesis were identified by correlation analysis of gene expression changes induced by MeJA. Not much in the way of novel insight was offered, in part because transcription regulation is, in general, poorly understood. As an aside, Figure 5 was cited before Figure 4, and this should be corrected.

Response: Thank you for your kind advice. We have added more analysis related to transcription factors, such as the relative time of induction of each TF (Supplementary Data set 10). Also, the figures have been extensively revised and the numbering should now be in the correct order.

Reviewer 1 Comment 5

It is fair to say the authors have done a lot of work, providing the first genome sequence for a plant of the order Celastrales, and characterizing the catalytic function for a cytochrome P450 (CYP728B70) that is clearly important for triptolide biosynthesis. However, they fell notably short of the ultimate goal, to reconstitute the biosynthetic pathway in a heterologous organism. On the other hand, they did engineer yeast to make dehydroabietic acid, arguably an important intermediate goal.

Response: We thank the reviewer for the positive comments about our work, which we believe lays a solid foundation for elucidation of the biosynthetic pathway of triptolide.

Reviewer 1 Comment 6

Lastly, their figures are much too complex; especially Figure 3, where the details are so small they are basically illegible. Given the inevitable word limits, it would be better if the authors would decide what is essential, devote more wording in their captions to explain what is depicted, and relegate any omitted materials to the online supplements.

Response: We thank the reviewer for this comment. Figure 3 was indeed too complex and we have attempted to simplify this, as well as all the figures in the revised manuscript.

Reviewer #2

Reviewer 2 Comment 1

At this point, the multi-omics-guided workflow for studying plant secondary metabolism highlighted in this paper has become well established in the field. The fact that the whole approach only identified one gene potentially involved in triptolide biosynthesis suggests such approach is less effective than what the authors claimed.

Response: We agree that we may have overstated the novelty of the employed approach and have extensively revised the manuscript to more simply note that we applied a multi-omics approach towards elucidation of triptolide biosynthesis.

Reviewer 2 Comment 2

The genome sequencing effort did not seem to help gene discovery for triptolide biosynthesis. Folding it into this paper renders this potentially valuable data set not adequately explored.

Response: While we agree that the focus here on triptolide biosynthesis may overshadow the utility of the generated genome sequence, we have used this to analyze genome evolution. In addition, the improved genome sequence now reported in the revised manuscript led to discovery of the pairing of *TwCPS1* and *TwMS*.

Reviewer 2 Comment 3

The genome-wide analysis of transcription factors without functional testing is also not relevant to the story.

Response: We agree that this is not a focus of this report, and have revised the manuscript to reduce the emphasis on these. Nevertheless, given that we have the data in-hand, we have analyzed the induction pattern for the identified transcription factors and reported it here (Supplemental Data set 10), as this provides valuable information for future studies.

Reviewer 2 Comment 4

When the authors extracted metabolites from MJ-treated cells, "60 mg aliquots were soaked in 1.5 mL of 80% (v/v) methanol overnight at room temperature and then dissolved in an ultrasonic water bath for 60 min". It is not clear why the authors decided to do overnight extraction. Unstable intermediates might not be preserved under such protocol.

Response: Actually, in order to detect and quantify all of compounds including the unstable intermediates, we compared three extraction methods using 80% (v/v) methanol at the beginning of the study (Su *et al.*, 2018; Hansen *et al.*, 2017): **a)** 60 mg aliquots were extracted with 1.5 mL of 80% (v/v) methanol at room temperature for 60 min in an ultrasonic water bath; **b)** 60 mg aliquots were extracted three times with 1.5 mL of 80% (v/v) methanol at room temperature for 60 min in an ultrasonic water bath; **c)** 60 mg aliquots were soaked in 1.5 mL of 80% (v/v) methanol overnight at room temperature and then dissolved in an ultrasonic water bath for 60 min. The OPLS-DA analysis was performed to determine the difference in metabolites of the three methods. An ANOVA with a significance level of P <0.05 and max-fold > 2 was subsequently performed on the doubly filtered peaks to identify the difference in metabolites (Table 1). The results indicated that **method c** was better than **method b** and **method a**, both for the numbers and detection levels of the targeted compounds (Table 1 and 2), including several potential intermediates (e.g. triptophenolide, triptinin B, and triptoquinonide)(Su *et al.*, 2018), as shown in Supplementary Figure 13 and 14, leading to selection of this methodology.

Table 1 The OPLS-DA analysis of three methods

Groups	Types of compounds (295-400m/z)	
**a** vs. **b**	**a>b**	1
	**b>a**	3
**a** vs. **c**	**a>c**	3
	**c>a**	10
**b** vs. **c**	**b>c**	1
	**c>b**	6

Table 2 Extraction results of terpenoids by three methods

Methods	Triptolide (ug/g)	Triptophenolide (ug/g)	Celastrol (ug/g)
**a**	78.9	405.4	1020.8
**b**	78.9	407.2	1042.4
**c**	83.2	426.2	1073.0

Reference

1. Su P, *et al*. Identification and functional characterization of diterpene synthases for triptolide biosynthesis from *Tripterygium wilfordii*. *Plant J* **93**, 50-65 (2018).

2. Hansen NL, *et al*. The terpene synthase gene family in *Tripterygium wilfordii*

harbors a labdane-type diterpene synthase among the monoterpene synthase TPS-b subfamily. *Plant J* **89**, 429-441 (2017).

Reviewer 2 Comment 5

It is noted that, when the authors characterized TwCYP728B70 in yeast, a different extraction and detection method were employed (in this case GC-MS). Would this suggest the original 80% methanol extraction coupled with the UPLC/Q-TOF MS analysis might miss the intermediates derived from miltiradiene? If this were the case, the "gene-to-metabolite" analysis would be greatly limited.

Response: The difference in extraction protocol is due to the simpler metabolite profile of the recombinant yeast relative to plant material, which enabled use of a more rapid method that still allowed for detection of the relevant compounds, as previously employed (Su *et al*., 2018).

Reference

1. Su P, *et al*. Identification and functional characterization of diterpene synthases for triptolide biosynthesis from *Tripterygium wilfordii*. *Plant J* **93**, 50-65 (2018).

Reviewer 2 Comment 6

It is obvious, even without any experiments, that CYPs will be involved. Had the authors just performed RNA-seq analysis of the suspension cells and searched for *T. wilfordii* specific CYPs, TwCYP728B70 would nevertheless stand out. The point here is that the power of the multi-omics approach might be greatly limited due to the experimental design/analytic capacity.

Response: While we agree that, in principle, the reported work might not have been required to identify CYP728B70 as playing a role in triptolide biosynthesis, we note that our study provides an extensive foundation for further investigations, imparting substantial additional impact.

Reviewer 2 Comment 7

In the overexpression experiment described in Fig. 3f, it is perplexing that the level of triptolide suddenly increased at day 9, given that the transcription level of TwCYP728B70 remained constant from day 5 to day 9. Also, the increased expression of TwCYP728B70 was only observed in day 3 and that quickly dropped to the basal level for the rest of the experiment (all the way to day 9). These observations should be discussed.

Response: As requested, we have discussed these observations in the revised manuscript, as easily found in the document with the changes highlighted (line 293).

Reviewer 2 Comment 8

The authors tested the functions of the other 9 candidate CYPs by in vitro microsome assay. The microsome assay can be complicated. Some control experiments should be included to show that active CYPs are present.

Response: While we further investigated the other 9 candidate CYPs by whole-cell feeding studies, we were still were unable to detect activity. Regardless, although we would agree that it is unclear if the other CYP candidates are functional in the utilized microsome assays, our ability to carry out characterization of CYP728B70 demonstrates that we have at least identified the expected activity for initial transformation of the olefin intermediate miltiradiene in triptolide biosynthesis. Moreover, while the additional CYPs that seem to be involved in triptolide biosynthesis from our RNAi studies will be pursued in future studies, we would argue that our results reported here with CYP728B70 are sufficient to indicate the utility of the foundational gene-to-metabolite data sets, and resulting map, reported here.

Reviewer 2 Comment 9

The substrates tested do not seem to be water soluble. Did the authors prepare the stock solution in organic solution and then add to the assays? If that were the case, would the addition of organic solvent affect the enzyme activity?

Response: We prepared the substrate stock solutions using methanol solution and then add to the assays (methanol level is under 1%). The effect on enzyme activity seems to be negligible with such limited addition of methanol solvent, as we have previously shown (Guo *et al*., 2013, Guo *et al*., 2016).

Reference
1. Guo J, *et al*. CYP76AH1 catalyzes turnover of miltiradiene in tanshinones biosynthesis and enables heterologous production of ferruginol in yeasts. *Proc Natl Acad Sci USA* **110**, 12108-12113 (2013).
2. Guo J, *et al*. Cytochrome P450 promiscuity leads to a bifurcating biosynthetic pathway for tanshinones. *New Phytol* **210**, 525-534 (2016).

Reviewer 2 Comment 10
“Did the substrate remain dissolved?”

Response: Here we tested the solubility of these substrates in the assay (100 mM Tris-HCl, pH 7.5) compared with the equivalent substrates in methanol, as shown below. The results confirmed that all the substrates (dehydroabietic acid, triptinin B, triptophenolide and triptoquinonide) remain solvated to some extent in the assay.

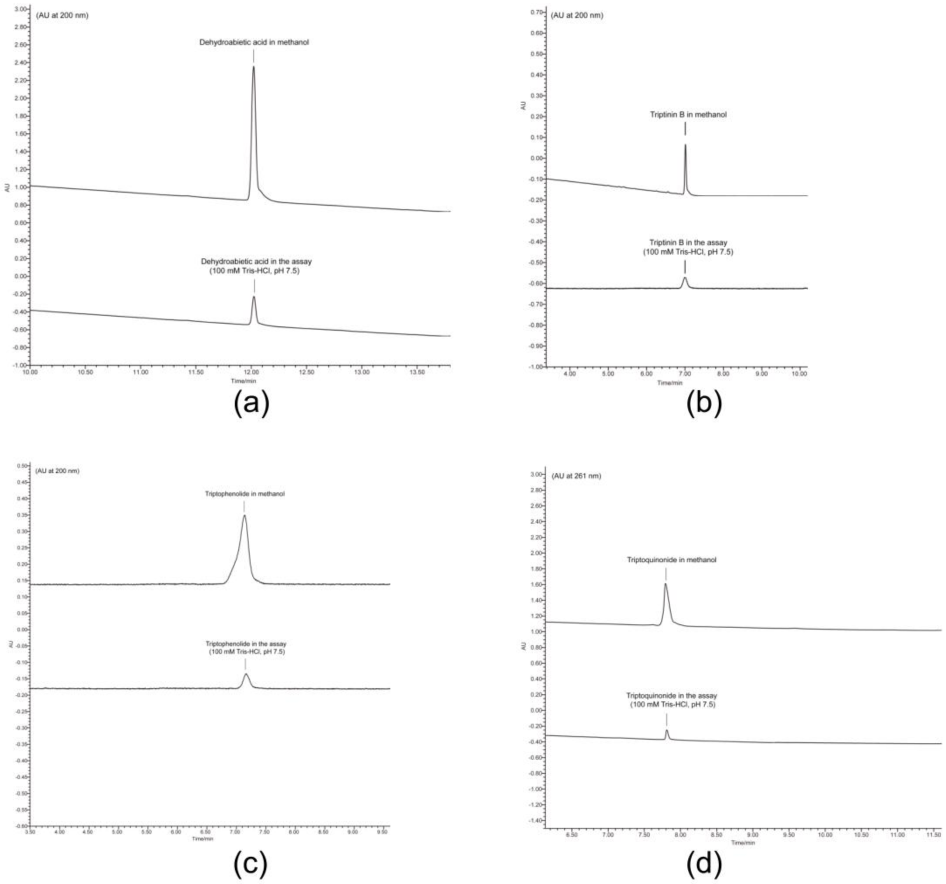

Measurement of the solubility of the utilized substrates. **a**, dehydroabietic acid. **b**, triptinin B. **c**, triptophenolide. **d**, triptoquinonide.

Reviewer 2 Comment 11
Details of extraction and purification procedure of compound 1 to 4 should be included.

Response: As requested, we added details of extraction and purification procedure of compound 1 to 4 in the revised manuscript, and the results are now showed in Supplementary Figure 23.

Reviewer #3

Reviewer 3 Comment 1
Most notably, the authors present the approach taken here as a novel strategy (termed MAEBLC). While clearly cutting edge, correlation of combined omics approaches for gene-metabolite network analysis has been used in prior studies and does not per se present a newly thought-out strategy. The authors should consider rephrasing the corresponding statements. Along the same lines, the authors state that this strategy now enables the discovery of pathways that are 'currently intractable' (line 333), which is an overstatement given the increasing number of studies demonstrating different successful approaches for identifying species-specific secondary metabolite pathways. Likewise, the authors describe triptolide as having 'remarkable antitumor activities' and 'a new blockbuster' drug'. Again, while triptolide clearly has important therapeutic potential as indicated by (pre-)clinical trials, it is far from being an approved drug. The relevance of the target metabolite should be stated in this context.

Response: We agree that we may have overstated the novelty of the employed approach and have extensively revised the manuscript to more simply note that we applied a multi-omics approach towards elucidation of triptolide biosynthesis. In addition, we also deleted 'currently intractable' from the Discussion section and, to reflect the fact that triptolide is not yet an approved drug, we revised the Introduction section as well.

Reviewer 3 Comment 2
The authors identify 10 P450 candidates based their gene-metabolite network analysis and characterize one as a terpenoid-metabolic P450 of the CYP728 family. Did the other candidates belong to the same family or to P450 families previously shown to be involved in terpenoid-metabolism? A more detailed description of the identified candidates would provide a more comprehensive picture of the divergence of P450s in Tripterygium and the spectrum of P450 families with possible roles in this pathways.

Response: Thank you for the kind suggestion. Here, we provided family information for 10 candidates in Supplementary Table 20. The characterized CYP728B70 is the only CYP728 family member. The other CYPs belong to CYP716C, CYP81AM, CYP76A, CYP76Y, CYP82J, CYP82AS CYP712K, CYP94C (sub)families, of which the CYP82 family is not well-known to participate in terpenoid metabolism.

As requested, we added a neighbor-joining tree which provide a more

comprehensive picture of the divergence of P450s in *T. wilfordii* (Supplementary Figure 34). Based on this, we hope to investigate the roles of these CYPs in the CYP728 and terpenoid-metabolism P450 families in future work.

Reviewer 3 Comment 3

The description of identified transcription factor networks is somewhat superficial and reads like a late addition to the study. While knowledge of the regulatory elements controlling secondary metabolite pathways is of great importance, a deeper analysis would be valuable here. For example, a genomic and/or phylogenetic analysis of select TF candidates with those identified in other plant species would already provide a deeper insight into possible candidates for future studies.

Response: We agree that this is not a focus of this report, and have revised the manuscript to reduce the emphasis on these. Nevertheless, given that we have the data in-hand, we have analyzed the induction pattern for the identified transcription factors and reported it here (Supplemental Data set 10), as this provides valuable information for future studies.

Reviewer 3 Comment 4

Figure 3a: The figure is very hard to read and does not provide much insight beyond highlighting the complexity of the network. A clearer presentation, for example, buy highlighting relevant correlation would be helpful.

Response: We thank the reviewer for this comment. Figure 3 was indeed too complex and we have attempted to simplify this, as well as all the figures in the revised manuscript.

Reviewer 3 Comment 5

- A minor suggestion for the title: ‘foundation for the investigation of’ or ‘foundation for investigating’
- Line 57: the contaminating impact of Tripterygium pollen on honey should be described in more detail.
- Line 57: ‘at’ a large scale
- Line 79: Original works on the identification of triptolide-metabolic diterpene synthases should cited (Su et al. 2018 Plant Journal 93:50-65; Hansen et al. 2017 Plant Journal 89:429-41).
- Line 97: It would be helpful to describe the novel de novo assembler in more

detail here. What is the advantage over previously established approaches?
- Line 148: GGPPS instead of GGPS?
- Line 187: Tripterygium should be in italics
- Line 240: ‘intermediates’ instead of ‘intermeidates’

Response: We thank the Reviewer for these careful observations, and we have revised them according to your kind suggestion.

- The title has been modified to “Multi-omic analysis of *Tripterygium wilfordii* provides a foundation for investigating triptolide biosynthesis”.
- We have added the descriptions of the contaminating impact of *Tripterygium* pollen on honey.
- ‘on’ a large scale has been revised to ‘at’ a large scale.
- We have cited the original works on the identification of triptolide-metabolic diterpene synthases.
- We have re-assembled the genome of *Tripterygium wilfordii* by PacBio sequencing technologies (~207X), and finally contig N50 was increased from 28.6 Kb to 4.36 Mb, which was written in the section of “Methods”.
- ‘GGPS’ has been revised to ‘GGPPS’.
- ‘Tripterygium’ has been revised to ‘*Tripterygium*’.
- ‘intermeidates’ has been revised to ‘intermediates’.

Reviewer #4

Reviewer 4 Comment 1

Throughout the manuscript, authors describe about the multi-omics approach to elucidating the biosynthesis of low-abundance compounds (MAEBLC) but I do not this is new approach, but rather same as what has been used for many years. Authors used transcriptome and metabolome correlation analysis for a time series dataset, and used genome comparison to identify genes specific to their target plant. I think this is a common classical practice and not enough to emphasize so much in this article. In fact, one of the reason for me to review this article was after reading this article but was disappointment after completing this article.

Response: We agree that we may have overstated the novelty of the employed approach and have extensively revised the manuscript to more simply note that we applied a multi-omics approach towards elucidation of triptolide biosynthesis.

Reviewer 4 Comment 2

While I agree that this is an important resource, the quality of final genome assembly for me is not impressive and probably the only week point of this study. Authors assembled this genome with scaffold and contig N50 as 1.3Mb and 28Kb, respectively. This genome seems highly fragmented, which is little disappointing since even Mb levels contigs are becoming a norm these days. Total number of contigs and scaffolds are 90681 and 73038, respectively. I strongly feel that authors could do much better job on this. I recommend to probably add more long read sequencing datasets and test different parameters to get final assembly. Since their primary assembly is fragmented, they could not get a high quality genome by using 10x data. Wheat genome for instance have very similar sequencing strategy as this plant but were able to achieve an amazing genome assembly at the end.

Response: We agree with the reviewer's suggestion that improving genome quality would significantly add to this study, and have expended considerable effort towards this. In particular, 75.79 Gb Pacbio long reads (~207.10× coverage of the genome), 119.75 Gb 10X Genomics sequencing data (~327.23× coverage), and 54.77Gb Hi-C sequencing data were generated, and all of these reads were assembled into 348.53 Mb with 467 contigs and 321 scaffolds with a contig N50 of 4.36 Mb and a scaffold N50 of 13.52 Mb. Approximately 94.40% of the Illumina reads could be mapped to the assembly using BWA, which covered 98.86% of the genome assembly. And more than 97.06% of RNA unigenes could be identified in the genome assembly using BLAT. In addition, the CEGMA results confirmed the homologs for 96.77% of the core eukaryotic genes in the assembly. BUSCO analysis revealed that 95.10% of the genes in the assembly were conserved. This new work is described in the "Sequencing, assembly and annotation" section of the revised manuscript and Supplementary Information.

Reviewer 4 Comment 3

I not agree with authors synteny analysis and interpretation, given such a fragmented genome for this species. Authors in line no-135 states, “Intergenomic co-linearity analysis was consistent with both the γ-event and another, more specific WGD event for T. wilfordii, as indicated by a 1:2 syntenic relationship between T. wilfordii and Vitis vinifera (Supplementary Fig. 7, Supplementary Data 1).” How authors can say this, one can see multiple syntenic regions from V. vinifera getting connected at multiple sites for their species. Further, I am assuming that authors simply selected scaffolds of certain size to o this analysis and not entire genome. If this is the case, authors need to mention that.

Response: We now report *de novo* assembly of a higher-quality genome

consisting of 23 chromosomes, with a contig N50 of 4.36 Mb. Intergenomic co-linearity were re-analyzed, which indicates 1:3 syntenic relationship between *T. wilfordii* and *Vitis vinifera*. This is described in detail in the "Genome evolution contributed to formation of triptolide" section of the revised manuscript and "Whole genome triplication (WGT)" section of the Supplementary Information document.

Reviewer 4 Comment 4

Indeed, the 10 genes identified by authors were induced by JA treatment and highly expressed in root bark. So, genome analysis although does support this, but really not essential. That's one of the reason why I expected high genome assembly quality as this allows to look at many different aspects of evolution and gene clusters that could serve as a resource for future discoveries.

Response: Thank you for your kind suggestion. As described in our revised manuscript, based on the higher quality genome for *T. wilfordii* that we have now generated, we discovered paring of the *TwCPS1* and *TwMS* that encode the cyclases that initiate triptolide biosynthesis (in chromosome 21; Supplementary Figure 10). Although this region on chromosome 21 also contains a *CYP* (TW023804.1) that exhibits a potentially relevant expression pattern, our results indicate that this may not be involved in triptolide biosynthesis. There also are several transcription factors, whose relevance is similarly unknown, but will be investigated in future studies as well.

Reviewer 4 Comment 5

Authors described whole genome duplication for their species, and did described on genes related to terpenoid biosynthesis. The conclusion was that duplication of involved genes in this plant species occurred post whole genome duplication. Since the genome quality is not good, unfortunately, authors were not able to make statement if these genes showed locational duplication? This is important aspect for the evolution of especially metabolites and a big gap that authors missed to address here.

Response: Thank you for the suggestion. Based on our now higher quality genome sequence we are now able to present data regarding gene location (Supplementary Data set 2).

Reviewer 4 Comment 6

I am curious about association between metabolome and transcriptome association. I understand that authors used correlation to identify genes co-expressed with their metabolite of interest. But as we know, JA treatment causes induction of genes and the response, which is change in metabolite levels, takes more time to appear. Is this the same case in their study? What are the early and late responsive genes in response to JA treatment? How about transcription factors? “But as we know, JA treatment causes induction of genes and the response, which is change in metabolite levels, takes more time to appear. Is this the same case in their study?”

Response: Thank you for your kind suggestion. A similar pattern, with transcription levels induced well before changes are seen in metabolite levels, is in fact observed here for triptolide biosynthesis. Specifically, most of the genes encoding enzymes involved in the triptolide biosynthesis had induced increases in transcription levels at 4 h, 12 h, and 24 h, but metabolite levels were only significantly increased after 36 h. We describe this in detail in the “Metabolite and transcript profiling of the triptolide biosynthetic pathway” section of the Supplementary Information, as well as in Figure 2 and Supplementary Figure 11.

Reviewer 4 Comment 7

“What are the early and late responsive genes in response to JA treatment? How about transcription factors?” Is it possible that transcription factors that are induced at the early time point could regulate genes expressed at late time point, some of which involved in triptolide biosynthesis. There are many such interesting questions that authors if try to address will improve this manuscript significantly.

Response: Thank you for your kind advice. We have analyzed the relative time of induction of each transcription factor (TF) and found most of TFs showed changes in transcription levels at 4 h and 12 h, of which 235 were up-regulated at 4 h and 162 were up-regulated at 12 h. These results are reported in Supplementary Data sets 10-12 and described in the “Multi-level regulation of triptolide” section of the Supplementary Information. Investigation of the TFs relevant to triptolide biosynthesis will be carried out in future studies.

Reviewer 4 Comment 8

I am also curious about levels of the newly identified four metabolites in response to CYP728B70, these metabolites were not identified or induced in response to JA treatment even post 240 hrs post treatment?

Response: We have detected the newly identified four metabolites by UPLC/Q-TOF MS, but we didn't detect them in the suspension cells even post 240 hrs. We assume that these metabolites are rapidly converted to downstream products the subsequently acting enzymes in planta.

REVIEWERS' COMMENTS:

Reviewer #1 (Remarks to the Author):

The authors have made a good faith effort to address all my concerns, as well as those of the other 3 reviewers (at least to my eyes) going so far as to generate a much improved genome sequence at undoubtedly greater cost. I have no further comments and hereby approve the paper for publication.

Reviewer #2 (Remarks to the Author):

The authors fixed some of the minor comments in my last review (not fully), but essentially left my main criticisms unaddressed. I reiterate these main criticisms below:

1) The whole multi-omics approach did not effectively yield much advance towards elucidation of the triptolide biosynthesis. Only one P450 (CYP728B70) was identified and characterized, which was also not definitely confirmed to be involved in the triptolide biosynthetic pathway. The entire pathway is essentially left unresolved.

2) The narrative of the story needs a major overhaul. The way that the genome sequence data were folded into this triptolide biosynthetic narrative does not do the justice to this dataset.

3) As also mentioned by other reviewers, the multi-omics approach, which was a main highlight of the study, is no longer that novel measured by today's standards. This further reduces the impact of this paper in light of its other major shortcomings.

Reviewer #3 (Remarks to the Author):

After review of this revised version of the manuscript I conclude that the authors have overall addressed my earlier concerns and comments.

A few minor comments are as follows:

1. The authors have much improved the manuscript by removing the hype and frequent mention of novelty. In this context, I recommend to also replace ‘remarkable’ in sentence one of the abstract with more toned down and scientific terms such ‘substantial’, ‘promising’ or potential’.

2. Figure 3 is much improved for clarity and readability. However, the authors may consider moving part (A) into the supplements, since the network diagram and individual color themes are still very hard to differentiate.

3. In the discussion, the authors may want to consider to more strongly highlight that the combination of coexpression patterns, tissue-specificity, and inducibility (rather than these factors individually) are critical to efficiently identify candidate genes in non-model systems with complex natural product pathways.

4. Again in the discussion, the authors could consider to comment on the fact that the step-wise carboxylation reaction observed for CYP728B70 (as the first function demonstrated for the CYP728 family) is catalytically highly similar to the CYP720B family in conifers that even forms some common products, thus showcasing an intriguing case of independent evolution of these functions in distant plant species.

Reviewer #4 (Remarks to the Author):

Thank you so much for such a nice work. I am so impressed that the authors managed to address all my comments and I can see a significant improvement in the manuscript. The genome assembly, its interpretation, and the biological meaning out of this study will help to advance this field.

I favor this study to be published in Nature communication. I do believe that authors need to improve writing for the main text and supplementary method sections, but I am sure that it will be taken care of at the proofreading stage.

Response to reviewers

Reviewer #1

Reviewer 1 Comment 1
The authors have made a good faith effort to address all my concerns, as well as those of the other 3 reviewers (at least to my eyes) going so far as to generate a much improved genome sequence at undoubtedly greater cost. I have no further comments and hereby approve the paper for publication.

Response: We thank the reviewer for the positive comments about our work. Also, thank you very much for your valuable suggestions on *CYP* gene analysis, transcription factor analysis, figure modification, and manuscript writing to improve the quality of our manuscript.

Reviewer #2

Reviewer 2 Comment 1
The whole multi-omics approach did not effectively yield much advance towards elucidation of the triptolide biosynthesis. Only one P450 (CYP728B70) was identified and characterized, which was also not definitely confirmed to be involved in the triptolide biosynthetic pathway. The entire pathway is essentially left unresolved.

Response: In this work, we integrated multi-omics data to map a gene-to-metabolite network, providing us candidate *CYP* genes. Then we identified and characterized one P450 (CYP728B70) that can catalyze oxidation of a methyl to the acid moiety of dehydroabietic acid, and the RNAi knock-down and overexpression experiments indicated this P450 was involved in the triptolide biosynthetic pathway. Furthermore, we used CYP728B70 to engineer yeast for the production of dehydroabietic acid, which will contribute to the identification of candidate genes encoding subsequently acting enzymes. We think this work will accelerate the process to resolve entire pathway of triptolide.

Reviewer 2 Comment 2
The narrative of the story needs a major overhaul. The way that the genome sequence data were folded into this triptolide biosynthetic narrative does not do the justice to this dataset.

Response: As requested, we have revised the narrative of the story in the revised

manuscript, and we thank the reviewer for this valuable comment.

Reviewer 2 Comment 3
As also mentioned by other reviewers, the multi-omics approach, which was a main highlight of the study, is no longer that novel measured by today's standards. This further reduces the impact of this paper in light of its other major shortcomings.

Response: We thank the reviewer for this comment. In the revised manuscript, We have modified the related statements about the multi-omics approach.

In this work, we report a reference-grade genome of *T. wilfordii* and show that copy numbers of triptolide biosynthetic pathway genes are impacted by a recent whole genome triplication event. We integrate genomic, transcriptomic, and metabolomic data to map a gene-to-metabolite network which leads to the identification of a cytochrome P450 (CYP728B70) that can catalyze oxidation of a methyl to the acid moiety of dehydroabietic acid in triptolide biosynthesis. We think the genomic resource and the candidate genes reported here will help to fully reveal triptolide biosynthesis pathway.

Reviewer #3

Reviewer 3 Comment 1
The authors have much improved the manuscript by removing the hype and frequent mention of novelty. In this context, I recommend to also replace 'remarkable' in sentence one of the abstract with more toned down and scientific terms such 'substantial', 'promising' or potential'.

Response: We thank the reviewer for this comment and we have revised the Abstract section.

Reviewer 3 Comment 2
Figure 3 is much improved for clarity and readability. However, the authors may consider moving part (A) into the supplements, since the network diagram and individual color themes are still very hard to differentiate.

Response: Thank you for the kind suggestion and we have moved part (A) in Figure 3 into Supplementary information.

Reviewer 3 Comment 3
In the discussion, the authors may want to consider to more strongly highlight that the combination of coexpression patterns, tissue-specificity, and inducibility (rather than these factors individually) are critical to efficiently identify candidate genes in non-model systems with complex natural product pathways.

Response: We thank the reviewer for this comment, we have discussed these factors in the Discussion section, as easily found in the revised manuscript with the changes tracked.

Reviewer 3 Comment 4
Again in the discussion, the authors could consider to comment on the fact that the step-wise carboxylation reaction observed for CYP728B70 (as the first function demonstrated for the CYP728 family) is catalytically highly similar to the CYP720B family in conifers that even forms some common products, thus showcasing an intriguing case of independent evolution of these functions in distant plant species.

Response: Thank you for the valuable suggestion, and we have added this comment in the Discussion section.

Reviewer #4

Reviewer 4 Comment 1
Thank you so much for such a nice work. I am so impressed that the authors managed to address all my comments and I can see a significant improvement in the manuscript. The genome assembly, its interpretation, and the biological meaning out of this study will help to advance this field.<br><br>I favor this study to be published in Nature communication. I do believe that authors need to improve writing for the main text and supplementary method sections, but I am sure that it will be taken care of at the proofreading stage.

Response: We thank the reviewer for the positive comments about our work. Also, thank you very much for your valuable suggestions on the genome sequencing, genome analysis, transcription factor analysis, and manuscript writing to improve the quality of our manuscript. And we have asked a colleague

to review our manuscript to improve writing for the main text and supplementary method sections.
